# A clinically compatible drug‐screening platform based on organotypic cultures identifies vulnerabilities to prevent and treat brain metastasis

**DOI:** 10.15252/emmm.202114552

**Published:** 2022-02-17

**Authors:** Lucía Zhu, Diana Retana, Pedro García‐Gómez, Laura Álvaro‐Espinosa, Neibla Priego, Mariam Masmudi‐Martín, Natalia Yebra, Lauritz Miarka, Elena Hernández‐Encinas, Carmen Blanco‐Aparicio, Sonia Martínez, Cecilia Sobrino, Nuria Ajenjo, Maria‐Jesus Artiga, Eva Ortega‐Paino, Raúl Torres‐Ruiz, Sandra Rodríguez‐Perales, RENACER, Adolfo de la Lama‐Zaragoza, Lourdes Calero‐Felix, Concepcion Fiaño‐Valverde, Pedro David Delgado‐López, Antonio Montalvo‐Afonso, Mar Pascual‐Llorente, Ángela Díaz‐Piqueras, SH Nam‐Cha, Cristina Barrena López, Gerard Plans Ahicart, Elena Martínez‐Saez, Santiago Ramón y Cajal, Pilar Nicolás, Riccardo Soffietti, Luca Bertero, Paola Cassoni, Tobias Weiss, Javier Muñoz, Juan Manuel Sepúlveda, Pedro González‐León, Luis Jiménez‐Roldán, Luis Miguel Moreno, Olga Esteban, Ángel Pérez‐Núñez, Aurelio Hernández‐Laín, Oscar Toldos, Yolanda Ruano, Lucía Alcázar, Guillermo Blasco, José Fernández‐Alén, Eduardo Caleiras, Miguel Lafarga, Diego Megías, Osvaldo Graña‐Castro, Carolina Nör, Michael D Taylor, Leonie S Young, Damir Varešlija, Nicola Cosgrove, Fergus J Couch, Lorena Cussó, Manuel Desco, Silvana Mouron, Miguel Quintela‐Fandino, Michael Weller, Joaquín Pastor, Manuel Valiente

**Affiliations:** ^1^ Brain Metastasis Group CNIO Madrid Spain; ^2^ Experimental Therapeutics Programme CNIO Madrid Spain; ^3^ Biobank CNIO Madrid Spain; ^4^ Molecular Cytogenetics Unit CNIO Madrid Spain; ^5^ Division of Hematopoietic Innovative Therapies Centro de Investigaciones Energeticas Medioambientales y Tecnologicas (CIEMAT) Madrid Spain; ^6^ Department of Neuro‐Oncology University and City of Health and Science Hospital Turin Italy; ^7^ Department of Medical Sciences University of Turin Turin Italy; ^8^ Department of Neurology Clinical Neuroscience Center University Hospital Zurich and University of Zurich Zurich Switzerland; ^9^ Proteomics Unit ProteoRedISCIII CNIO Madrid Spain; ^10^ Neuro‐Oncology Unit Hospital Universitario 12 de Octubre Madrid Spain; ^11^ Neurosurgery Unit Hospital Universitario 12 de Octubre Madrid Spain; ^12^ Department of Surgery Universidad Complutense de Madrid Madrid Spain; ^13^ Neuropathology Unit Instituto i+12, Hospital Universitario 12 de Octubre Madrid Spain; ^14^ Neuro‐Oncology Group Research Institute Hospital 12 de Octubre (i+12) Madrid Spain; ^15^ Pathology Department Instituto i+12, Hospital Universitario 12 de Octubre Madrid Spain; ^16^ Universidad Francisco de Vitoria Madrid Spain; ^17^ Neurosurgery Department Hospital Universitario de La Princesa Madrid Spain; ^18^ Histopathology Unit CNIO Madrid Spain; ^19^ Department of Anatomy and Cell Biology and Centro de Investigación Biomédica en Red sobre Enfermedades Neurodegenerativas (CIBERNED) University of Cantabria‐IDIVAL Santander Spain; ^20^ Confocal Microscopy Unit CNIO Madrid Spain; ^21^ Bioinformatics Unit CNIO Madrid Spain; ^22^ Developmental and Stem Cell Biology Program and The Arthur and Sonia Labatt Brain Tumour Research Centre The Hospital for Sick Children Toronto ON Canada; ^23^ Endocrine Oncology Research Group Department of Surgery RCSI University of Medicine and Health Sciences Dublin Ireland; ^24^ Department of Laboratory Medicine and Pathology Mayo Clinic Rochester MN USA; ^25^ Departamento de Bioingeniería e Ingeniería Aeroespacial Universidad Carlos III de Madrid Madrid Spain; ^26^ Instituto de Investigación Sanitaria Gregorio Marañón Madrid Spain; ^27^ Centro de Investigación Biomédica en Red de Salud Mental (CIBERSAM) Madrid Spain; ^28^ Unidad de Imagen Avanzada Centro Nacional de Investigaciones Cardiovasculares (CNIC) Madrid Spain; ^29^ Breast Cancer Clinical Research Unit CNIO Madrid Spain; ^30^ Present address: Cell Signaling and Clinical Proteomics Group Biocruces Bizkaia Health Research Institute Barakaldo Spain; ^31^ Present address: Ikerbasque Basque Foundation for Science Bilbao Spain

**Keywords:** drug‐screen, metastasis, organotypic cultures, patient‐derived, resistance, Cancer, Pharmacology & Drug Discovery

## Abstract

We report a medium‐throughput drug‐screening platform (METPlatform) based on organotypic cultures that allows to evaluate inhibitors against metastases growing *in situ*. By applying this approach to the unmet clinical need of brain metastasis, we identified several vulnerabilities. Among them, a blood–brain barrier permeable HSP90 inhibitor showed high potency against mouse and human brain metastases at clinically relevant stages of the disease, including a novel model of local relapse after neurosurgery. Furthermore, *in situ* proteomic analysis applied to metastases treated with the chaperone inhibitor uncovered a novel molecular program in brain metastasis, which includes biomarkers of poor prognosis and actionable mechanisms of resistance. Our work validates METPlatform as a potent resource for metastasis research integrating drug‐screening and unbiased omic approaches that is compatible with human samples. Thus, this clinically relevant strategy is aimed to personalize the management of metastatic disease in the brain and elsewhere.

## Graphical abstract

A novel drug‐screening platform compatible with patient‐derived samples identifies effective therapies to prevent brain metastasis.

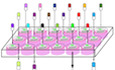

**The paper explained**

**Problem**

Brain metastasis is an unmet clinical need that currently affects up to 25% of cancer patients. A major issue remains the lack of knowledge on the vulnerabilities that, if properly exploited, could generate therapeutic opportunities.

**Result**

We report a novel drug‐screening platform to study vulnerabilities of metastasis during their growth in the organ being colonized. This platform, based on organotypic cultures, effectively identified compounds that were later validated *in vivo*, is compatible with unbiased omics approaches, and is fully applicable to human samples.

**Impact**

Our results offer a novel therapeutic strategy that could be applicable to prevent brain metastasis in a clinically relevant context. Furthermore, we demonstrate that METPlatform should be considered as a potential approach to facilitate the management of metastatic disease in the context of personalized cancer care.

## Introduction

The incidence of brain metastasis continues to increase, yet current therapies available for patients with disseminated cancer cells in their central nervous system (CNS) have a limited efficacy and fail to improve survival (Valiente *et al*, 2018; Moravan *et al*, 2020; Suh *et al*, 2020).

Consequently, during the past years, there have been recurrent efforts to improve clinical trial design and management specifically concerning this patient population (Lin *et al*, 2013a, 2013b, 2015; Le Rhun *et al*, 2021). However, the inclusion of patients with active CNS disease has been limited in the trials of the past, and this represents an unsolved issue (Arvold *et al*, 2016). As a result, information regarding CNS clinical efficacy of most anti‐cancer agents that are FDA‐approved or in clinical trials is limited. Thus, exploring therapeutic vulnerabilities and corresponding pharmacological agents with high CNS activity in preclinical models are crucial to promote urgently needed prospective clinical trials that include patients with brain metastases (Camidge *et al*, 2018).

*In vivo* drug‐screening using mouse models that faithfully recapitulate the clinical phenotype imposes high demand of economic costs, time, and resources (Gao *et al*, 2015) that are unaffordable by most academical research institutions. On the other hand, cell‐based assays lack the contribution of the tumor‐associated microenvironment, which has gained relevance in the context of response to therapy during recent years (Hirata & Sahai, 2017). In this regard, the brain microenvironment is a key aspect in the biology of CNS metastasis (Boire *et al*, 2020) that has been demonstrated to limit therapeutic benefits of systemic therapy (Chen *et al*, 2016).

To overcome limitations of both *in vivo* and *in vitro* approaches, we report an *ex vivo* organotypic culture‐based drug‐screening system: METPlatform. We use this strategy to evaluate the impact of different therapeutic agents on metastases growing *in situ* (i.e., the brain), thus identifying biologically relevant drug candidates in a rapid and cost‐effective manner.

Brain organotypic cultures have been used in cancer research due to their ability to mimic the progression of metastatic disease locally (Zhu & Valiente, 2021). They resemble both early (Valiente *et al*, 2014; Er *et al*, 2018) and advanced stages of the disease (Priego *et al*, 2018). Their versatility allows exploring diverse functional and mechanistic insights of brain metastasis, including the interaction between cancer cells and different components of the microenvironment using genetic or pharmacologic approaches (Valiente *et al*, 2014; Er *et al*, 2018; Priego *et al*, 2018). However, to the best of our knowledge, their use for drug‐screening has not been reported. We describe here the use of brain organotypic cultures for performing a medium‐throughput screening using an in‐house library of anti‐cancer agents, FDA‐approved, or under clinical development (Bejarano *et al*, 2019), with unknown or limited information regarding their activity in the CNS.

In addition to other hits, METPlatform identified inhibitors of heat shock protein 90 (HSP90) as a potential target to increase the vulnerability of brain metastasis. HSP90 is a molecular chaperone required for correct protein folding, intracellular disposition, and proteolytic turnover of its client proteins, and therefore essential for cellular proteostasis (Schopf *et al*, 2017). It is heavily exploited by cancer cells not only to maintain numerous pro‐survival oncoproteins and transcription factors, but also to buffer proteotoxic stress induced during oncogenic transformation and progression (Whitesell & Lindquist, 2005) as well as to regulate mechanisms of immune evasion (Fionda *et al*, 2009; Kawabe *et al*, 2009). High HSP90 expression levels have been correlated with poor prognosis in all subtypes of breast cancer patients (Pick *et al*, 2007; Dimas *et al*, 2018), several independent cohorts of non‐small cell lung cancer (NSCLC) patients (Gallegos Ruiz *et al*, 2008), and in colorectal cancer (Kim *et al*, 2019).

Following METPlatform identification of HSP90 as a potential target, we show the potent anti‐metastatic activity of a second‐generation HSP90 inhibitor, DEBIO‐0932, in experimental and human metastases. Furthermore, we use METPlatform to dissect the underlying biology downstream of HSP90 inhibition using unbiased proteomics to identify novel mediators of brain metastasis, biomarkers of the disease, and combination strategies to overcome resistance.

As a final proof‐of‐concept, we show that METPlatform could be additionally exploited as a clinically compatible “avatar” to predict the therapeutic response of patients with brain tumors.

## Results

### A chemical library applied to METPlatform identifies potential vulnerabilities of brain metastasis

**Figure 1:**
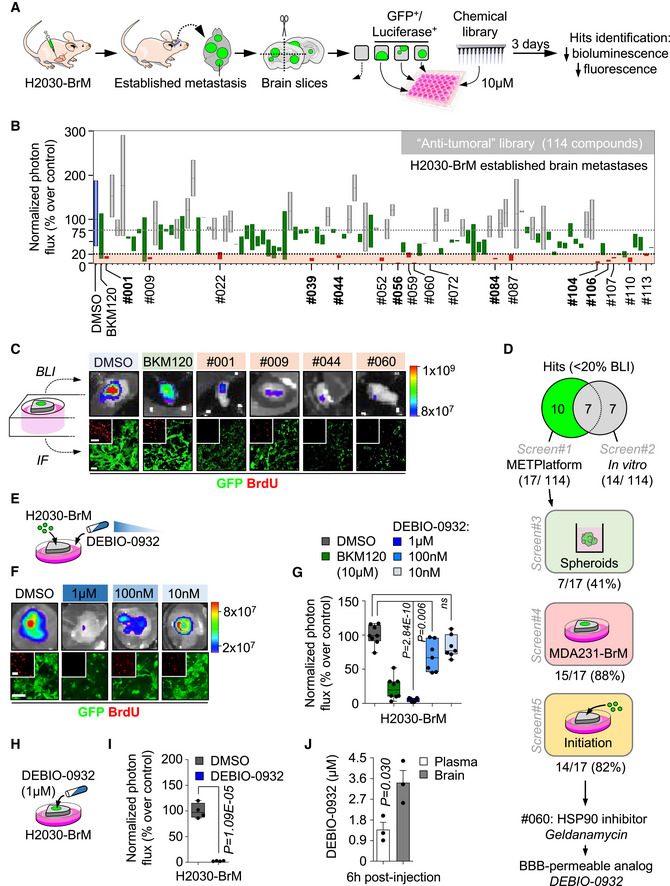
A chemical library applied to METPlatform identifies potential vulnerabilities of brain metastasis Schema of the experimental design.Quantification of the bioluminescence signal emitted by established H2030‐BrM brain metastases in each organotypic culture at day 3 normalized by their initial value at day 0 (before the addition of DMSO or any compound). The final value in the graph is normalized to the organotypic cultures treated with DMSO. Blue: DMSO‐treated organotypic cultures; red: hits, compounds with normalized BLI ≤ 20%; green: BKM120 and compounds with similar efficacy to BKM120; gray: compounds that do not reduce BLI values. Values are shown in box‐and‐whisker plots where the line in the box corresponds to the mean. Boxes extend from the minimum to the maximum value (*n* = 28 DMSO; *n* = 21 BKM120‐treated organotypic cultures; each experimental compound of the library was assayed by duplicate, 8 independent experiments). Hits highlighted in bold are common to those obtained in the *in vitro* screening (Fig EV1A). Gray dashed line indicates the minimum decrease in BLI (25%) that we considered as a positive phenotype. The black dashed line represents 80% decrease in BLI, which identifies top hits.Representative images of bioluminescence (BLI) and histology of organotypic cultures with established brain metastases from H2030‐BrM treated with DMSO, BKM120 or the indicated hits. Cancer cells are in green (GFP) and proliferative cells are in red (BrdU). Scale bar: 75 µm.Venn diagram showing the number of hits *ex vivo* (17) and *in vitro* (14) and common to both approaches (7). Compounds tested in additional screens (screen#3: H2030‐BrM spheroids; screen#4: established MDA231‐BrM breast cancer brain metastasis; and screen#5: metastasis initiation H2030‐BrM) only include those considered as hits *ex vivo* in panel B. Number of hits in each screen are indicated over the total number of hits obtained in screen#1 (B).Schema of the experimental design. Organotypic cultures with H2030‐BrM cells mimicking the early steps of colonization were used to perform dose‐response optimization with DEBIO‐0932.Representative BLI and histology of organotypic cultures with H2030‐BrM cancer cells treated with DMSO or decreasing concentrations of DEBIO‐0932. Scale bar: 100 µm; high magnification: 50 µm.Quantification of the bioluminescence signal emitted by each condition shown in (F) at Day 3 normalized by the initial value obtained at Day 0 and normalized to the organotypic cultures treated with DMSO. Day 0 is considered 12–16 h after the addition of cancer cells and treatment or DMSO. Values are shown in box‐and‐whisker plots where each dot is an organotypic culture and the line in the box corresponds to the median. The boxes go from the upper to the lower quartiles and the whiskers go from the minimum to the maximum value (*n* = 8 DMSO, *n* = 8 BKM120 and *n* = 7 per concentration of DEBIO‐0932‐treated organotypic cultures, 2 independent experiments). *P* value was calculated using two‐tailed *t*‐test.Schema of the experimental design. Organotypic cultures with H2030‐BrM established metastases were used to test the efficacy of DEBIO‐0932.Quantification of the bioluminescence signal emitted by H2030‐BrM established metastases in organotypic cultures at Day 3 normalized by the initial value obtained at Day 0 and normalized to the organotypic cultures treated with DMSO. Day 0 is considered right before addition of the treatment or DMSO. Values are shown in box‐and‐whisker plots where each dot is an organotypic culture and the line in the box corresponds to the median. The boxes go from the upper to the lower quartiles and the whiskers go from the minimum to the maximum value (*n* = 4 organotypic cultures per experimental condition, 2 independent experiments). *P* value was calculated using two‐tailed *t*‐test.Quantification of the concentration of DEBIO‐0932 reached in animals harboring H2030‐BrM established brain metastases 6 h after oral administration of DEBIO‐0932 at 160 mg/kg. The concentration was measured in both the plasma and the brain for each mouse. Values are shown as mean + s.e.m. (*n* = 3 mice per experimental condition). *P* value was calculated using two‐tailed *t*‐test.

Given our interest in targeting clinically relevant stages of brain metastasis, we used METPlatform to study vulnerabilities of macrometastases. The human lung adenocarcinoma brain metastatic (BrM) cell line H2030‐BrM (Nguyen *et al*, 2009) was injected intracardially into athymic nude mice to obtain fully established brain metastases at clinical endpoint of the animals. Brains were processed into organotypic cultures, and the efficacy of the anti‐tumoral library (Table EV1) was evaluated at a concentration of 10 µM (Fig 1A). Of note, established methods to assess the viability of this preparation such as LDH detection from dead cells showed a slight increase during the initial stages of culture preparation, which could be associated with sample processing since it gets stabilized during culture (Appendix Fig S1A). Given the expression of luciferase and GFP in the H2030‐BrM model (Nguyen *et al*, 2009), the impact of specific inhibitors on the viability of brain metastases in organotypic cultures was assessed by bioluminescence imaging (BLI) and immunofluorescence against GFP in comparison with DMSO‐treated cultures. We used a PI3K inhibitor, BKM120, as an internal positive control in our experiments due to the known involvement of this signaling pathway and therapeutic benefit in brain metastasis (Nanni *et al*, 2012; Brastianos *et al*, 2015; Pistilli *et al*, 2018). In addition to reproduce the efficacy of BKM120, METPlatform identified additional compounds that are superior in their ability to compromise the viability of established brain metastasis (Fig 1B and C). Top hits were selected by reducing in 80% or more the bioluminescence values that correspond to controls treated with DMSO (Fig 1B). This threshold was confirmed to be a good correlate of compromised viability based on a complementary histological analysis (Fig 1C). The analysis of the drug‐screen provided us with 17 hits: carfilzomib (#1), dovitinib (#9), trametinib (#22), mitomycin C (#39), GSK2126458 (#44), AT7519 (#52), CNIO‐DUAL (#56), sorafenib (#59), geldanamycin (#60), SN‐38 (#72), bortezomib (#84), KU‐57788 (#87), CNIO‐TRIPLE (#104), crizotinib (#106), CNIO‐ATR (#107), pazopanib (#110), and linifanib (#113) out of 114 compounds tested (Fig 1B and C, Table EV1).

**Figure EV1:**
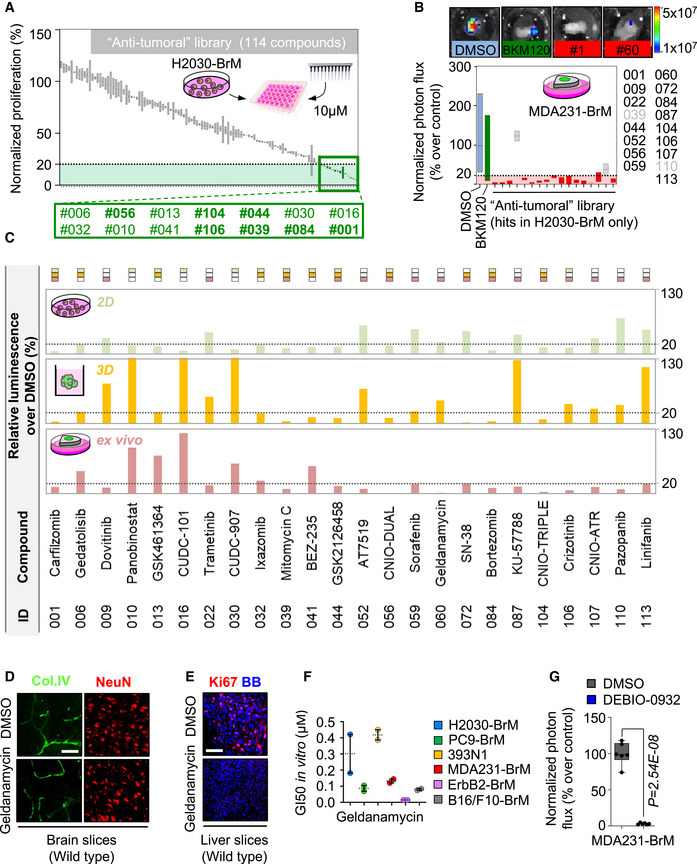
A chemical library applied to METPlatform identifies potential vulnerabilities of brain metastasis Quantification of the proliferation of H2030‐BrM cells at day 3 normalized to the cells treated with DMSO measured with CellTiter‐Glo^®^. Green: hits, compounds with ≤ 20% proliferation; gray: compounds with > 20% proliferation. Values are shown in box‐and‐whisker plots where the line in the box corresponds to the mean. Each experimental compound of the library was assayed by duplicate. Hits highlighted in bold were common to the *ex vivo* screening (Fig 1B).Quantification of the bioluminescence signal from MDA231‐BrM established brain metastases in organotypic culture after 3 days in culture. Values were normalized by the level of bioluminescence at Day 0 for each culture (before the addition of DMSO or any compound). Final data is shown in percentage respect to reference, the organotypic cultures treated with DMSO. Blue: DMSO‐treated organotypic cultures; red: hits, compounds with normalized BLI ≤ 20% (dashed line); green: BKM120; gray: compounds with normalized BLI > 20%. Values are shown in box‐and‐whisker plots where the line in the box corresponds to the mean. Boxes extend from the minimum to the maximum value (*n* = 14 DMSO; *n* = 13 BKM120‐treated organotypic cultures; each experimental compound was assayed by duplicate, 4 independent experiments).Detailed representation of the data shown in Figs 1B, EV1A and Table EV1 indicating relative viability using bioluminescence generated by H2030‐BrM cells *ex vivo* (established brain metastases, light red), *in vitro* 2D (green) and *in vitro* 3D (spheroids, yellow) treated with compounds of the anti‐tumoral library (compounds were assayed by duplicate in each assay). All hits for any condition are shown. The rectangles of the top indicate whether a given compound was effective (< 20% luminescence respect to control) *ex vivo* (light red rectangle), *in vitro* 2D (green rectangle), *in vitro* 3D (yellow rectangle).Representative wild‐type brain slices treated with DMSO or the HSP90 inhibitor geldanamycin stained with anti‐Col.IV (endothelial cells) and anti‐NeuN (neurons). Scale bar: 50 µm.Representative wild‐type liver slices treated with DMSO or the HSP90 inhibitor geldanamycin and stained with anti‐Ki67 to score proliferation. BB: bisbenzamide. Scale bar: 50 µm.Quantification of GI_50_ values of geldanamycin in a panel of BrM cell lines *in vitro* from various primary origins and oncogenomic profiles. Nine serial concentrations of geldanamycin were assayed by duplicate and GI_50_ was calculated from a viability curve normalized to DMSO‐treated cells of the corresponding cell line. Values are shown as mean + s.e.m. (each concentration was assayed by technical duplicates for each cell line and the experiment was performed twice).Quantification of the bioluminescence signal emitted by MDA231‐BrM established metastases in organotypic cultures incubated in the presence of DEBIO‐0932 (1 µM) during 3 days. Bioluminescence at Day 3 is normalized by the initial value obtained at day D and quantified relative to the organotypic cultures treated with DMSO. Day 0 is considered right before addition of the treatment or DMSO. Values are shown in box‐and‐whisker plots where each dot is an organotypic culture and the line in the box corresponds to the median. The boxes go from the upper to the lower quartiles and the whiskers go from the minimum to the maximum value (*n* = 6 organotypic cultures per experimental condition, 1 experiment). *P* value was calculated using two‐tailed *t*‐test.

To compare METPlatform with a traditional cell‐based assay as a drug‐screening platform, we applied the same chemical library to H2030‐BrM cells cultured *in vitro* (Fig EV1A). Interestingly, after applying the same criteria based on luminescence, only 7 out of 14 hits obtained *in vitro* were part of the 17 hits obtained with METPlatform (Figs 1D and EV1C, Table EV1). Even if these hits were applied to H2030‐BrM spheroids, only 7 out of 17 also scored (Figs 1D and EV1C, Table EV1). Thus, METPlatform selected hits that would not have been considered as such with other established approaches.

We extended our *ex vivo* drug‐screen to a triple‐negative breast cancer brain metastasis model, MDA231‐BrM (Bos *et al*, 2009), to identify vulnerabilities regardless the primary tumor origin. Out of the 17 hits tested, 15 of them decreased the viability of cancer cells in 80% or more as measured by BLI (Figs 1D and EV1B, Table EV1). In addition, we used METPlatform to analyze whether any hit also scored not only against advanced stages of the disease when metastases are fully established (Fig 1B), but also against the initial steps of organ colonization, which could be mimicked *ex vivo* by plating cancer cells on top of tumor‐free organotypic brain cultures (Valiente *et al*, 2014). Interestingly, 14 out of 17 hits inhibited both early and advanced stages of brain metastasis (Fig 1D, Table EV1), which suggests that these compounds may not only be effective treating, but also preventing metastasis outgrowth by acting on the initiation of organ colonization. On the other hand, reported differences in the biology of initial and established brain metastases (Valiente *et al*, 2014; Priego *et al*, 2018) could be exploited therapeutically by interrogating those hits only scoring in one or another stage of colonization (dovitinib (#9), pazopanib (#110), and linifanib (#113)) (Table EV1).

Finally, METPlatform also allows simultaneous evaluation of the potential toxicity derived from selected compounds on non‐cancer cell types and in different organs. For instance, the use of specific markers for various brain cell types, such as neurons and endothelial cells, allowed us to discard a major unselective cytotoxicity in this organ (Fig EV1D). In contrast, evaluation of reported sensitive organs confirmed the ability of the drug‐screening platform to reproduce clinical toxicity (i.e., hepatotoxicity) (Fig EV1E; Supko *et al*, 1995).

Altogether, our results support METPlatform as a comprehensive and more informative drug‐screening platform in the context of metastasis compared to conventional cell‐based assays (Fig 1D, Table EV1).

In order to select compounds for further validation, we focused on those targeting not only established metastasis from different cancer types but also initial stages of organ colonization (Fig 1D, Table EV1). Out of this selection, we then focused on those that, although with inhibitory activity 2D and 3D *in vitro* (Fig EV1F), did not score as hits in this condition (Fig EV1A and C). With this selection criterion, we wanted to evaluate the potential of METPlatform to select hits working *in vivo*. Six hits fulfilled the criteria: trametinib (#22), AT7519 (#52), sorafenib (#59), geldanamycin (#60), KU‐57788 (#87), and CNIO‐ATR (#107). Unfortunately, METPlatform has no capacity to score blood–brain barrier (BBB)/blood–tumor barrier (BTB) permeability, and indeed, we failed to recognize this property among these compounds, suggesting that, when METPlatform is applied to metastasis in the brain, a previous step to prioritize BBB/ BTB‐permeable compounds should be incorporated to design the library (Saxena *et al*, 2019). Given the improved efficacy of brain permeable compounds to target metastasis in this organ (Osswald *et al*, 2016), we looked for alternative inhibitors focused on the targets identified. DEBIO‐0932, a second‐generation HSP90 inhibitor, has an improved toxicity profile in comparison with geldanamycin, increased bioavailability and, more importantly, a remarkable ability to cross the BBB (Supko *et al*, 1995; Bao *et al*, 2009). As geldanamycin, DEBIO‐0932 blunted the viability of initial and established brain metastases from lung (H2030‐BrM) and breast (MDA231‐BrM) cancer models in *ex vivo* assays (Figs 1 E–I and Fig EV1G, Table EV10). Furthermore, the concentration reached by DEBIO‐0932 in a brain affected by metastases (Fig 1J) is above the therapeutic levels as determined *ex vivo* (Fig 1E–I).

**Figure 2:**
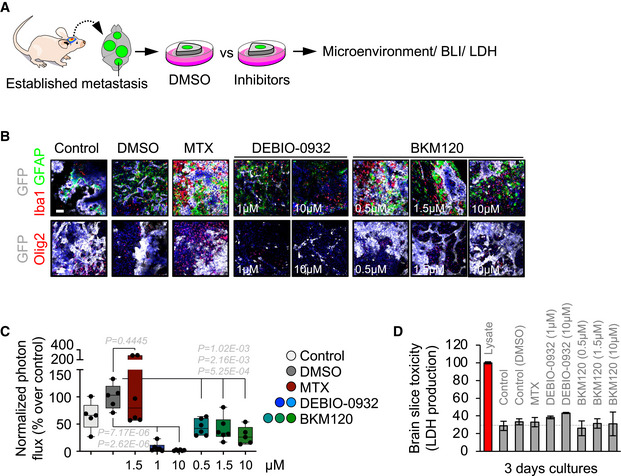
METPlatform is compatible with the evaluation of the metastasis‐associated microenvironment Schema of the experimental design.Representative images of organotypic cultures with established metastases with various glial components of the microenvironment labeled. Scale bar: 75 µm. Each individual condition was evaluated in several organotypic cultures (3–6 slices).Quantification of the bioluminescence signal emitted by established H2030‐BrM brain metastases in each organotypic culture at Day 3 normalized by their initial value at Day 0 (before the addition of DMSO or any compound). The final value in the graph is normalized to the organotypic cultures treated with DMSO. Values are shown in box‐and‐whisker plots where the line in the box corresponds to the mean. The boxes go from the upper to the lower quartiles and the whiskers go from the minimum to the maximum value (*n* = 5–6 organotypic cultures, 1 independent experiment). *P* value was calculated using two‐tailed *t*‐test.Quantification of LDH levels in the conditioned media of organotypic slices cultured during 3 days relative to a lysate of the same preparation. Values are shown as mean + s.e.m. (*n* = 3 organotypic cultures per experimental condition, 1 independent experiment).

Given the importance of the metastasis‐associated microenvironment for local disease progression (Boire *et al*, 2020), we evaluated in more detail this aspect in METPlatform (Fig 2A). First, we determine that the vehicle used was not influencing the brain microenvironment at the concentration used (Appendix Fig S1B). Second, we introduced inhibitors previously reported to influence glial cells such as methotrexate (MTX) (Gibson *et al*, 2019) and BKM120 (Blazquez *et al*, 2018). In comparison with DEBIO‐0932, MTX massively induced tumor‐associated microglia/macrophages and reactive astrocytes (Fig 2B) markers, although this was not translated into a compromise of metastasis viability as assessed by histology and bioluminescence (Fig 2B and C). Finally, although established methods for assessing major toxicity effects (i.e., LDH) did not reflect any major impact from any compound (Fig 2D), high concentrations of BKM120 and DEBIO‐0932 showed incipient signs of their impact on the tumor‐associated microenvironment (Fig 2B). Given that low concentrations used for DEBIO‐0932 had a major effect on the viability of metastatic cells (Figs 1F–I and 2C), we conclude that METPlatform not only identified potential vulnerabilities but it also allows to evaluate the differential sensitivity of cancer cells versus tumor‐associated microenvironment to a given drug. Given the limited efforts to test drugs currently available or under clinical trials in patients with brain metastasis, METPlatform provides an additional strategy to generate initial data on this potential application. As such, we identified DEBIO‐0932 as a potent inhibitor of brain metastases viability *ex vivo* that is able to accumulate in the brain at therapeutic concentrations.

### Brain metastases are positive for HSP90

**Figure 3:**
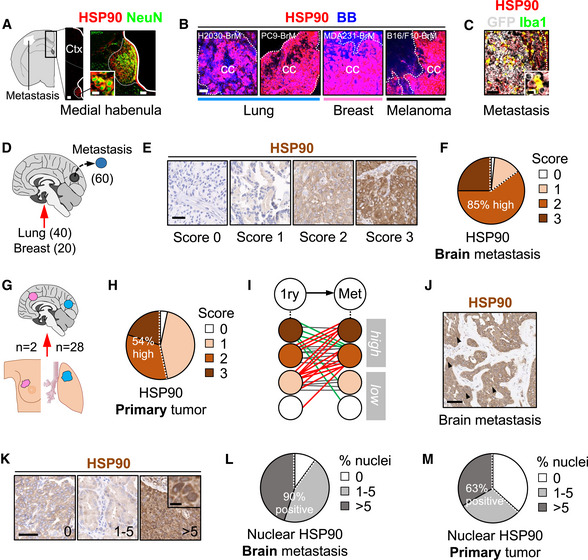
Brain metastases are positive for HSP90 A–CImmunofluorescence against HSP90 in mouse brains with established metastases. (A) HSP90 positive structures in areas not affected by the metastasis includes the medial habenula, where neurons co‐localize with the chaperone. Scale bars: 100 µm (low magnification), 50 µm (medial habenula nucleus), 12 µm (high magnification neurons). (B) Established metastases from different primary origins and oncogenomic profiles stained with HSP90. Dotted lines delineate the metastasis (cc: cancer cells). Scale bars: 75 µm. (C) Iba1 colocalizes with HSP90 within areas affected by metastases. BB: bisbenzamide. Scale bar: 75 µm (low magnification), 12 µm (high magnification).DImmunohistochemistry against HSP90 was performed in human brain metastases (*n* = 60) from lung (40 cases) and breast cancer (20 cases).ERepresentative human brain metastases showing different intensities or scores for HSP90. Scale bar: 50 µm.FQuantification of HSP90 in human brain metastases. 59 out of 60 (98%) showed positive staining of HSP90 in the tumor, 15 (25%) scored with 3 (strong), 36 (60%) with 2 (moderate), and 8 (13%) with 1 (weak) according to the signal intensity of HSP90 in the cytoplasm of cancer cells.GHuman brain metastases (*n* = 30) and their matched primary tumors (*n* = 28 lung and *n* = 2 breast) were evaluated and compared for HSP90 expression by immunohistochemistry.HQuantification of HSP90 in human primary tumors. 29 out of 30 (97%) showed positive staining of HSP90 in the tumor, 6 (20%) scored with 3 (strong), 10 (34%) with 2 (moderate), and 13 (43%) with 1 (weak) according to the signal intensity of HSP90 in the cytoplasm of cancer cells.ISchema showing HSP90 scores in matched pairs of primary tumor and brain metastasis. Red: increase of HSP90 score from primary to brain metastasis; green: decrease of HSP90 score; gray: no changes in HSP90 score.J, KRepresentative human brain metastases showing different percentages of nuclear HSP90. Scale bars: (J) 50 µm; (K) low magnification: 100 µm; high magnification: 10 µm. Black arrows point to cancer cells positive for HSP90 in the nucleus.LQuantification of nuclear HSP90 in human brain metastases. 54 out of 60 samples (90%) showed positive nuclear HSP90 in the tumor. 27 (45%) showed 1–5% (moderate) and 27 (45%) showed > 5% (high) of nuclear HSP90.MQuantification of nuclear HSP90 in human primary tumors. 19 out of 30 (63%) showed positive nuclear HSP90 in the tumor. 9 (30%) showed 1–5% (moderate) and 10 (33%) showed > 5% (high) of nuclear HSP90.

Before testing the potential benefits of DEBIO‐0932 *in vivo*, we evaluated the presence of its target in brain metastases. To evaluate HSP90 levels *in situ*, we performed tissue immunofluorescence in four experimental brain metastasis models from both human and mouse origin, characterized by different oncogenic drivers and derived from breast cancer, lung cancer, and melanoma, which are the most frequent sources of brain metastasis (Valiente *et al*, 2020). Established brain metastases obtained at experimental endpoint showed high HSP90 levels in cancer cells (Fig 3B). In sharp contrast, the unaffected brain did not show any positivity with the exception of specific neuronal nuclei, such as the medial habenula (Fig 3A). Of interest, metastasis‐associated Iba1+ microglia/macrophages showed high intensity of HSP90; however, they were outnumbered by HSP90^high^ cancer cells (Fig 3C). Thus, we focus our efforts on the characterization of the drug target in metastatic cells.

60 paraffin‐embedded human brain metastases from NSCLC (40 samples) and breast adenocarcinoma (20 samples) were stained with anti‐HSP90 by immunohistochemistry and blindly evaluated and scored by a pathologist (Fig 3D, Table EV2). 98% of brain metastases were positive for HSP90, with 85% of them showing moderate or strong staining of the protein (score ≥ 2, HSP90^high^) (Fig 3E and F), which is a value higher than previous reports on primary tumors (Pick *et al*, 2007; Gallegos Ruiz *et al*, 2008; Kim *et al*, 2019). To investigate this possibility, we scored 30 matched primary tumors (Fig 3G) and confirmed a lower percentage (54%) of samples scoring as HSP90^high^ in comparison to brain metastases (Fig 3H). When comparing matched pairs of a primary tumor and a brain metastasis, 13/30 (43%) brain metastases had increased HSP90 levels compared to the primary tumor, from which 10/13 (77%) switched from HSP90^low^ (score ≤ 1) to HSP90^high^ (score ≥ 2). 12/30 (40%) matched pairs showed equal HSP90 levels; however, 8/12 (67%) cases were HSP90^high^ in the primary tumor to start with. Out of the 5/30 (17%) brain metastases with lower HSP90 than the corresponding primary tumor, 3/5 (60%) cases still remained within the HSP90^high^ category and only 2/5 (40%) switched from HSP90^high^ to HSP90^low^ (Fig 3I).

Although HSP90 is primarily a cytoplasmic protein, several studies have described its role in nuclear events such as transcriptional processes, chromatin remodeling, and DNA damage (Trepel *et al*, 2010; Antonova *et al*, 2019). Moreover, increased nuclear HSP90 correlated positively with poor survival and distant metastasis in NSCLC patients (Su *et al*, 2016). Interestingly, we found nuclear staining of HSP90 in 90% of brain metastasis samples (Fig 3J–L), with 45% of them scoring as HSP90^high^ (> 5% of positive nuclei out of total tumor) according to a previously described criteria (Su *et al*, 2016) (Fig 3L). Similar to the previous analysis, we found fewer primary tumors (63%) positive for nuclear HSP90, with 33% of them scoring HSP90^high^ (Fig 3M). Nevertheless, due to the prevalent low percentage of positive nuclei observed in most samples (Fig 3J), we were not able to accurately assess a potential enrichment of nuclear HSP90 in brain metastases compared to their paired primary tumor.

Taken together, our results demonstrate that high levels of HSP90 in cancer cells are a frequent finding among human brain metastasis independently of the primary tumor. Indeed, a clear tendency to maintain or further increase the levels of this protein is evident when compared to matched primary tumors. Overall, these results support potential functional implications of HSP90 in human brain metastasis.

### Inhibition of HSP90 is effective to treat established brain metastasis

We used DEBIO‐0932 in preclinical models to study whether the results obtained with METPlatform could be translated *in vivo*.

**Figure 4:**
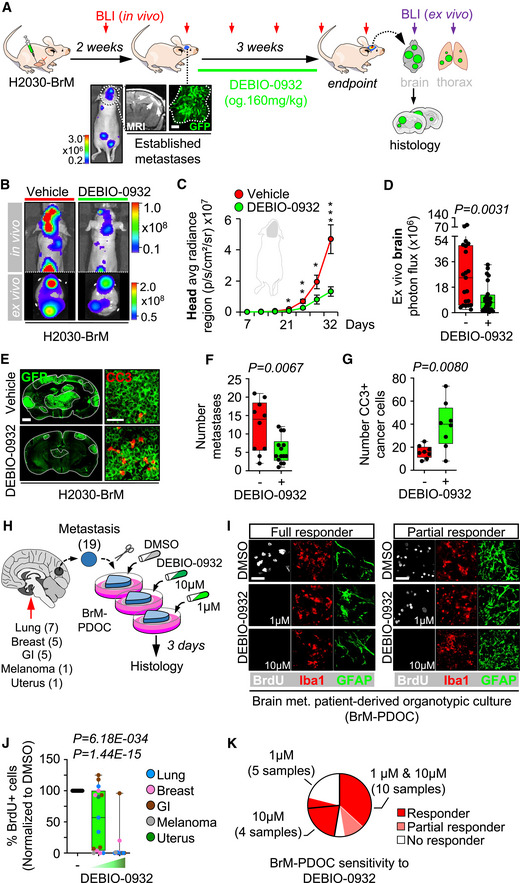
Inhibition of HSP90 is effective to treat established brain metastasis Schema of the experimental design. H2030‐BrM cells were inoculated intracardially into nude mice and established brain metastases were detected 2 weeks after by BLI, MRI (arrows) and histology (GFP^+^ cancer cells). DEBIO‐0932 was administered orally at 160 mg/kg for 3 weeks (daily during the first week and every 48 h during the two following weeks) and *ex vivo* BLI of brains and thoracic regions were analyzed. Brains were processed for histological analysis. Scale bar: 100 µm.Representative *in vivo* and *ex vivo* images of vehicle and DEBIO‐0932‐treated mice 5 weeks (experimental endpoint) after intracardiac inoculation of H2030‐BrM cells.Quantification of metastatic progression as measured by *in vivo* BLI of head of animals. Values are shown as mean ± s.e.m. (*n* = 23 vehicle and *n* = 25 DEBIO‐0932‐treated mice, 3 independent experiments). *P* value was calculated using two‐tailed *t*‐test (*P* values: *P < 0.05, **P < 0.01, ***P < 0.001).Quantification of *ex vivo* BLI of brains at the endpoint of the experiment. Values are shown in box‐and‐whisker plots where every dot represents a different animal and the line in the box corresponds to the median. The boxes go from the upper to the lower quartiles and the whiskers go from the minimum to the maximum value (*n* = 21 vehicle and *n* = 24 DEBIO‐0932‐treated mice, three independent experiments). *P* value was calculated using two‐tailed *t*‐test.Representative sections of brains from vehicle and DEBIO‐0932‐treated mice in (B–D). The dotted lines surround the metastases (GFP^+^). Representative field of view of metastasis stained with GFP and cleaved caspase 3. Scale bars: slices, 1 mm; cleaved caspase 3, 50 µm.Quantification of established metastases found in vehicle and DEBIO‐0932‐treated brains from panel (E). Values are shown in box‐and‐whisker plots where every dot represents a different brain and the line in the box corresponds to the median. The boxes go from the upper to the lower quartiles and the whiskers go from the minimum to the maximum value (vehicle: *n* = 10 brains; DEBIO‐0932: *n* = 14 brains). *P* value was calculated using two‐tailed *t*‐test.Quantification of number of cleaved caspase 3 (CC3^+^) in cancer cells found in vehicle and DEBIO‐0932‐treated brains from panel (E). Values are shown in box‐and‐whisker plots where every dot is a metastatic lesion and the line in the box corresponds to the median. The boxes go from the upper to the lower quartiles, and the whiskers go from the minimum to the maximum value (*n* = 8 metastatic lesions from 4 brains per condition). *P* value was calculated using two‐tailed *t*‐test.Schema of the experimental design. Fresh surgically resected human brain metastases (*n* = 19) from various primary origins were used to perform patient‐derived organotypic cultures (BrM‐PDOC) and treated with DEBIO‐0932 at 10 µM and 1 µM for 3 days.Representative BrM‐PDOC stained with proliferation markers (BrdU) and markers of the microenvironment (GFAP for astrocytes, Iba1 for microglia/ macrophages). Scale bar: 50 µm.Quantification of the relative number of BrdU^+^ cancer cells found in DMSO DEBIO‐0932‐treated BrM‐PDOC respect to the corresponding PDOC treated with DMSO. Values are shown in box‐and‐whisker plots where every dot represents a patient (mean value obtained from all PDOC from the same condition and patient) and the line in the box corresponds to the median. The boxes go from the upper to the lower quartiles, and the whiskers go from the minimum to the maximum value (*n* = 19 patients with DMSO‐treated PDOC, *n* = 14 DEBIO‐0932 10 µM and *n* = 15 DEBIO‐0932 1 µM, each patient is an independent experiment). *P* value was calculated using two‐tailed *t*‐test. Dots are colored according to the primary source of the metastasis.Pie chart showing all BrM‐PDOC in (J) classified according to the specific dose tested and the type of response observed. Partial responder means that the response was different depending on the dose of DEBIO‐0932, with PDOC not responding at 1 µM.

**Figure EV2:**
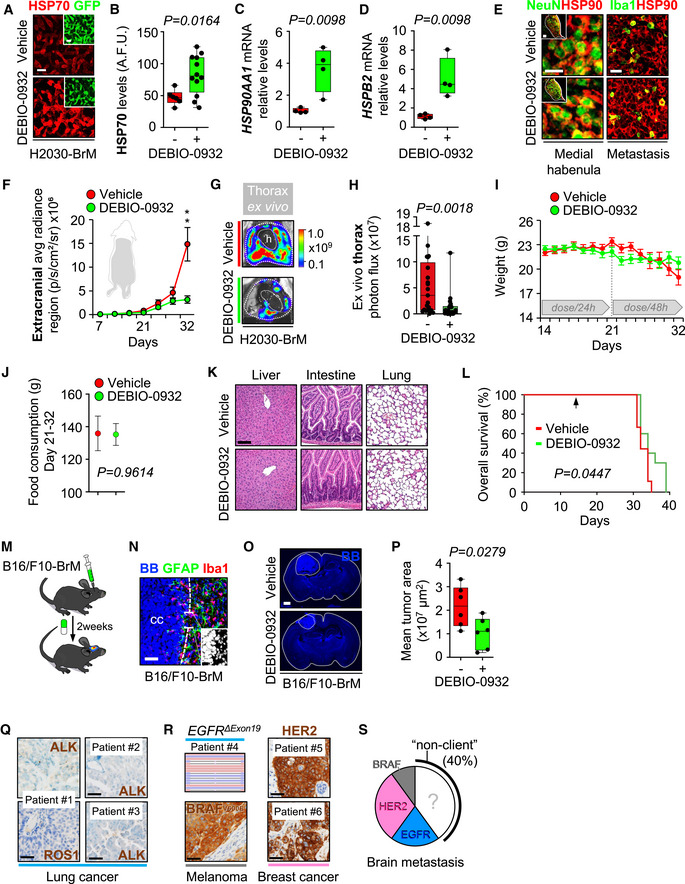
Inhibition of HSP90 is effective to treat established brain metastasis ARepresentative images showing HSP70 levels in brain metastases (generated by intracardiac inoculation of H2030‐BrM) found at endpoint of vehicle and DEBIO‐0932‐treated animals. Scale bar: 75 µm.BQuantification of HSP70 levels shown in (A) in arbitrary fluorescent units (A.F.U.). Values are shown in box‐and‐whisker plots where each dot is a metastatic lesion and the line in the box corresponds to the median. The boxes go from the upper to the lower quartiles, and the whiskers go from the minimum to the maximum value (*n* = 6–12 metastatic lesions from 3 to 6 brains per condition). P value was calculated using two‐tailed *t*‐test.C, D
*HSP90AA1* (C) and *HSPB2* (D) expression levels obtained by qRT–PCR of H2030‐BrM brain metastases obtained at endpoint of vehicle and DEBIO‐0932‐treated animals. Values are shown in box‐and‐whisker plots where every dot represents a different animal and the line in the box corresponds to the median. The boxes go from the upper to the lower quartiles, and the whiskers go from the minimum to the maximum value (*n* = 4 mice per experimental condition). *P* value was calculated using two‐tailed *t*‐test.ERepresentative images of HSP90^+^ non‐cancer cell compartments including the medial habenula and the Iba1^+^ microglia/macrophages in the metastasis‐associated microenvironment from vehicle and DEBIO‐0932‐treated brains at the endpoint of the experiment (Fig 4A). Scale bars: Medial habenula low magnification (nucleus): 50 µm; Medial habenula high magnification (cells): 12.5 µm; Metastasis: 32 µm.FQuantification of metastatic progression as measured by *in vivo* BLI of extracranial region of animals. Values are shown as mean ± s.e.m. (*n* = 23 vehicle and *n* = 25 DEBIO‐0932‐treated mice, 3 independent experiments). *P* value was calculated using two‐tailed *t*‐test (*P* values: **P < 0.01).GRepresentative images of thorax from vehicle and DEBIO‐0932‐treated mice at the endpoint of the experiment.HQuantification of *ex vivo* BLI of thoracic regions at the endpoint of the experiment. Values are shown in box‐and‐whisker plots where every dot represents a different animal and the line in the box corresponds to the median. The boxes go from the upper to the lower quartiles, and the whiskers go from the minimum to the maximum value. (*n* = 21 vehicle and *n* = 24 DEBIO‐0932‐treated mice, three independent experiments). *P* value was calculated using two‐tailed *t*‐test.IAnimal weight from vehicle and DEBIO‐0932‐treated mice during the treatment period. DEBIO‐0932 treatment started 2 weeks (day 14) after inoculation of cancer cells and was maintained for 3 weeks, once every 24 h during the first week and once every 48 h during the two following weeks. Values are shown as mean ± s.e.m. (*n* = 9 vehicle and *n* = 10 DEBIO‐0932‐treated mice).JQuantification of mean food consumption during the interval of time between 21 and 32 days in both vehicle and DEBIO‐0932‐treated mice. Values are shown as mean ± s.e.m. (*n* = 6 mice per experimental condition. Mice were divided in two individual cages per experimental condition with 3 mice each). *P* value was calculated using two‐tailed *t*‐test.KHematoxylin eosin staining of three organs from vehicle and DEBIO‐0932‐treated mice at experimental endpoint. (*n* = 3 mice per experimental condition were evaluated for each organ). Scale bar: 50 µm.LKaplan‐Meier curve comparing overall survival of vehicle and DEBIO‐0932‐treated mice following the schedule depicted in Fig 4A. (*n* = 9 mice treated with vehicle and *n* = 10 mice treated with DEBIO‐0932). *P* value was calculated using log‐rank (Mantel‐Cox) test. The arrow indicates when the treatment was initiated.MSchema of experimental design. The brain metastatic melanoma cell line B16/F10‐BrM was intracranially injected to generate an established tumor so the treatment could start 3 days post‐injection.NRepresentative image of an established tumor 3 days post‐injection. The interface between the metastasis and the associated microenvironment is well‐defined. Scale bar: 50 µm (low magnification); 25 µm (high magnification).ORepresentative images of slices with the brain tumor at the end of the experiment. BB: Bisbenzamide. Scale bar: 1 mm.PQuantification of the tumor area at experimental endpoint. Values are shown in box‐and‐whisker plots where every dot represents a different brain and the line in the box corresponds to the median. The boxes go from the upper to the lower quartiles and the whiskers go from the minimum to the maximum value (*n* = 6 mice per experimental condition). *P* value was calculated using two‐tailed *t*‐test.QRepresentative images of human brain metastases from which BrM‐PDOC were generated and evaluated as responders (Fig 4H) that showed no correlation with HSP90‐dependent oncogenic drivers ALK and ROS1. Scale bar: 50 µm.RRepresentative images of human brain metastases from which BrM‐PDOC were generated and evaluated as responders (Fig 4H) that showed positive correlation with HSP90‐dependent oncogenic drivers HER2 and BRAF. Scale bar: 50 µm. Targeting sequencing of the *EGFR* locus of a lung cancer brain metastasis patient showing a deletion in exon 19 is also shown.SPie chart showing the distribution of the ten BrM‐PDOCs with oncogenic drivers sensitive to HSP90 inhibition (Non‐HSP90 client: *n* = 4; *EGFR* mutant lung cancer: *n* = 2; HER2^+^ breast cancer: *n* = 3; *BRAF* mutant melanoma: *n* = 1).

Brain metastases were induced by intracardiac inoculation of H2030‐BrM cells (Nguyen *et al*, 2009). Two weeks after injection, we confirmed the presence of established metastases in the brain using BLI, histology, and magnetic resonance imaging (MRI) (Fig 4A). DEBIO‐0932 administration at 160 mg/kg during the following 3 weeks significantly impaired the growth of both brain metastases and extracranial lesions (Figs 4B–G and EV2F–H) by targeting HSP90 in cancer cells (Fig EV2A–D; Bagatell *et al*, 2000). We did not observe similar effects of DEBIO‐0932 in the microenvironment (Fig EV2E). These results were confirmed by brain and thorax *ex vivo* BLI (Figs 4B and EV2G and H) as well as histological quantification of dissected brains at the endpoint of the experiment, 5 weeks after cancer cell inoculation, including a reduction of metastases (Fig 4E and F) with an increased in cancer cell death (Fig 4E and G). Of note, we did not observe significant weight loss (Fig EV2I), food intake (Fig EV2J), or any other sign of toxicity after detailed multi‐organ histological analysis by an expert pathologist (Fig EV2K) in treated animals compared to the control group, ruling out major toxicities of DEBIO‐0932. Indeed, DEBIO‐0932 monotherapy increased survival of treated mice (Fig EV2L). However, rather than overinterpreting this significant but limited survival benefit, we use it as an added value reinforcing the need for further characterization of this therapeutic strategy derived from METPlatform. In this sense, treatment of established melanoma brain metastases (Fig EV2N) in an immunocompetent background (Priego *et al*, 2018) with DEBIO‐0932 confirmed the anti‐metastatic phenotype (Fig EV2M–P).

Among the many advantages of METPlatform, the possibility of adapting it to patient‐derived organotypic cultures (PDOC) using fresh surgically resected human tissue is invaluable for translational purposes.

Brain metastasis PDOC (BrM‐PDOC) were established from neurosurgical resections performed on nineteen patients diagnosed with five different types of primary tumor (Fig 4H) and a variety of oncogenic profiles (Fig EV2Q–S). Although all but one BrM‐PDOC responded to high dose of DEBIO‐0932 (Fig 4I and J), decreasing the dose to levels compatible to those detected in mouse brains with metastases (Fig 1J) correlated with the emergence of heterogeneity (Fig 4J). The origin of such heterogeneous therapeutic response did not correlate with similar changes in the microenvironment (Fig 4I) or any particular tumor origin (Fig 4J). Indeed, although 9/19 BrM‐PDOC were not compatible with testing two doses due to limited sample availability, only 20% of those receiving them showed divergent responses between high and low DEBIO‐0932 concentrations (Fig 4K), suggesting that inter‐patient differences are more likely to explain this phenotype. Overall, 74% of BrM‐PDOC are sensitive to DEBIO‐0932. In order to get to know the underlying molecular biomarker of HSP90 sensitivity and given that clinical response to HSP90 inhibitors has been attributed to “addiction” of tumors to particular oncogenes, such as *HER2*, *ALK*, *ROS1*, *EGFR,* and *BRAF*, which are sensitive HSP90 client proteins (Neckers & Workman, 2012), we had access to such information for a limited number of samples (10/19 samples). Among five brain metastases from NSCLC, two of them harbored a mutation/deletion in *EGFR* as detected by targeted sequencing (Fig EV2R); however, no molecular alterations in *EGFR*, *ALK,* and *ROS1* were found in the other three patients using standard methodologies approved in clinical practice for these biomarkers (Fig EV2Q). Additional molecular classifiers included three brain metastases derived from HER2^+^ breast adenocarcinomas, one from a melanoma with the activating mutation BRAF V600E (Fig EV2R), and one from a gastro‐esophageal cancer without known oncogenic drivers sensitive to HSP90 inhibition (Fig EV2S).

These results validate METPlatform as an effective *ex vivo* drug‐screening strategy for the identification of brain metastasis vulnerabilities, such as HSP90, that could be translated to *in vivo* metastasis assays. Our results also show that METPlatform could be used to validate experimental therapeutic strategies in human samples, where DEBIO‐0932 impairs the viability of the majority of BrM‐PDOC although with different response rates and independently of their primary tumor origin and established HSP90‐dependent oncogenes routinely scored in the clinical practice.

### Inhibition of HSP90 prevents brain metastasis initiation as well as local relapse post‐surgery

Approximately 20–40% of patients with brain metastasis receive neurosurgery. However, local relapse occurs in 60% of patients within one year after surgery and limits the benefits of an otherwise successful local therapy (Nahed *et al*, 2019; Dankner *et al*, 2021). To investigate whether DEBIO‐0932 is able to prevent this clinically relevant situation for which there is no established standard of care, we developed a first‐in‐class preclinical model of local relapse after brain metastasis neurosurgery.

**Figure 5:**
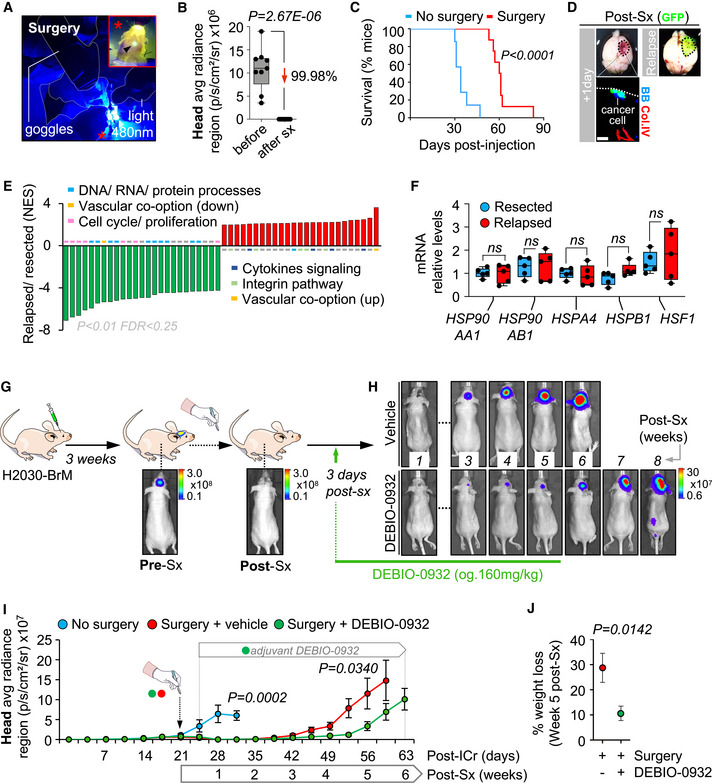
Inhibition of HSP90 prevents brain metastasis initiation as well as local relapse post‐surgery Detailed image of the neurosurgery procedure that visualizes the GFP+ brain tumor (high magnification) with a 480 nm light source and goggles equipped with emission filters. The asterisk in the low magnification labels the field of view for the surgeon, which is amplified in the high magnification through the emission filter equipped in the goggles. The arrow in the high magnification points to the GFP^+^ tumor as seen by the surgeon.Quantification of BLI values before and one day after neurosurgery. Values are shown in box‐and‐whisker plots where every dot represents a different animal and the line in the box corresponds to the median. The boxes go from the upper to the lower quartiles, and the whiskers go from the minimum to the maximum value (*n* = 9 mice before and after surgery). *P* value was calculated using two‐tailed *t*‐test.Kaplan‐Meier curve showing survival proportions of mice without (blue line, *n* = 7) and with surgery (red line, *n* = 8). *P* value was calculated using log‐rank (Mantel‐Cox) test.Representative images of brains one day after neurosurgery and at the endpoint of local relapse. Remaining cancer cells (GFP^+^) were found under the microscope in the surgical bed. GFP fluorescence of fully relapsed tumor at the experimental endpoint could be observed macroscopically. BB: bisbenzamide. Col.IV: collagen IV. Scale bar: 25 µm.GSEA of top 25 up‐ (red) and downregulated (green) signatures comparing matched relapsed and resected brain metastases from animals receiving neurosurgery.qRT–PCR of H2030‐BrM brain metastases obtained from animals during neurosurgery compared to relapsed metastases from the corresponding animals. A panel of five genes related to HSP90 pathway is evaluated. Values are shown in box‐and‐whisker plots where every dot represents a different animal and the line in the box corresponds to the median. The boxes go from the upper to the lower quartiles, and the whiskers go from the minimum to the maximum value (*n* = 5 mice per experimental condition). *P* value was calculated using two‐tailed *t*‐test.Schema of experimental design. H2030‐BrM cells were implanted intracranially into nude mice and established brain metastases were surgically resected. DEBIO‐0932 was administered orally at 160 mg/kg 3 days later and during 5–6 weeks following an individualized regimen. Sx: surgery.Representative images of vehicle and DEBIO‐0932‐treated mice after neurosurgery until experimental endpoint at 6 and 8 weeks for vehicle and DEBIO‐0932‐treated mice, respectively.Quantification of brain tumor progression as measured by *in vivo* BLI of head region in animals without surgery, with surgery and vehicle or DEBIO‐0932. DEBIO‐0932 treatment was initiated 3 days after surgery, which was applied 3 weeks post‐injection of BrM cells, and maintained for 5–6 weeks after local treatment. Values are shown as mean ± s.e.m. (*n* = 7 without surgery, *n* = 8 surgery + vehicle and *n* = 11 surgery + DEBIO‐0932‐treated mice, 2 independent experiments). *P* value was calculated using two‐tailed *t*‐test (No surgery *versus* surgery + vehicle (day 32), *P* = 0.0002; surgery + vehicle *versus* surgery + DEBIO‐0932 (day 56), *P* = 0.0340).Quantification of the percentage of weight loss at advanced stages of local relapse (week 5 post‐surgery). Values were obtained relative to the mean weight for each group at day 19, which corresponds to the highest weight value before any decrease could be detected. Values are shown as mean ± s.e.m. (*n* = 4 surgery + vehicle and *n* = 6 surgery + DEBIO‐0932‐treated mice, 1 experiment). *P* value was calculated using two‐tailed *t*‐test.

We modelled single brain macrometastasis by intracranial implantation of H2030‐BrM cells. This strategy facilitates the surgical approach avoiding non‐operable brains with multiple secondary tumors or surgically non‐accessible locations of metastasis (Valiente *et al*, 2020). Microsurgical resection of the metastasis guided by GFP was performed when BLI values reached 10^7^ photons/s/cm²/steradian (Fig 5A, B, G and I). Successful resection of the bulk tumor was confirmed in real time by the absence of macroscopically detectable GFP^+^ cancer cells and almost complete postsurgical reduction of BLI *in vivo* (Fig 5A, B and D), which doubled the survival time compared to those animals without local treatment (Fig 5C). However, the presence of single cancer cells left behind was also evident by microscopic analysis of the borders of the surgical bed one day after completing the local treatment (Fig 5D). These cancer cells are presumably responsible for the local relapse affecting all treated mice as tumors always reappeared within the same area where mass debulking was initially applied (Fig 5D and H). Full development of relapsed tumors occurred 5–6 weeks after surgery (Fig 5H and I).

We addressed differences between resected and relapsed tumors using transcriptomic profiling by RNAseq. Gene Set Enrichment Analysis of the transcriptomes from relapsed versus matched resected tumors showed downregulated signatures related to cell cycle and proliferation and enrichment in those related to vascular co‐option, a key mechanism during the early stages of organ colonization (Valiente *et al*, 2014; Er *et al*, 2018), and cytokine and integrin signaling (Fig 5E, Table EV3, Dataset EV1). In contrast, we validated that HSP90 coding genes and members of the heat shock response pathway were unaltered in relapsed tumors (Fig 5F, Table EV10).

**Figure EV3:**
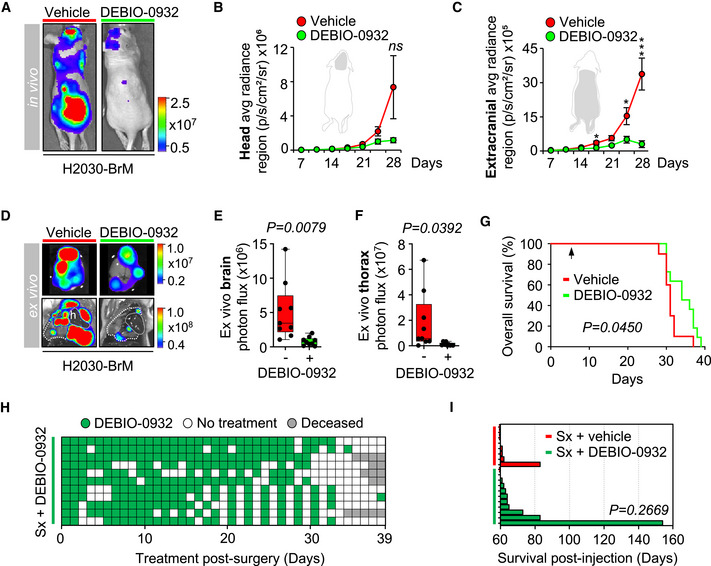
Inhibition of HSP90 prevents brain metastasis initiation as well as local relapse post‐surgery ARepresentative images of mice treated with DEBIO‐0932 (160 mg/kg, o.g.) starting at 7 days after intracardiac inoculation of H2030‐BrM cells. Treatment was given daily during the first week and every 48 h during the two following weeks.B, CQuantification of metastatic progression as measured by *in vivo* BLI of head (B) and extracranial region (C) of animals. Values are shown as mean ± s.e.m. (*n* = 9 vehicle and *n* = 9 DEBIO‐0932‐treated mice, 2 independent experiments). *P* value was calculated using two‐tailed *t*‐test (*P* values: *P < 0.05, ***P < 0.001).DRepresentative images of brains and thorax from vehicle and DEBIO‐0932‐treated mice at the endpoint of the experiment.E, FQuantification of *ex vivo* BLI of brains (E) and thoracic regions (F) at the endpoint of the experiment. Values are shown in box‐and‐whisker plots where every dot represents a different animal and the line in the box corresponds to the median. The boxes go from the upper to the lower quartiles, and the whiskers go from the minimum to the maximum value (*n* = 9 vehicle and *n* = 9 DEBIO‐0932‐treated mice, 2 independent experiments). *P* value was calculated using two‐tailed *t*‐test.GKaplan‐Meier curve comparing overall survival of vehicle and DEBIO‐0932‐treated mice starting 7 days post‐intracardiac injection (*n* = 10 mice treated with vehicle and *n* = 11 mice treated with DEBIO‐0932). *P* value was calculated using log‐rank (Mantel‐Cox) test. The arrow indicates when the treatment was initiated.HSchema showing the individualized therapy of mice receiving surgery + DEBIO‐0932 during the treatment period starting 3 days after surgery. Each row represents a mouse receiving DEBIO‐0932 (green) o not (white) (*n* = 11 surgery + DEBIO‐0932‐treated mice, 2 independent experiments). Gray squares indicate decease of the corresponding animal.IGraph showing survival of mice treated with surgery + vehicle or surgery + DEBIO‐0932. The graph represents each mouse with a bar only if the survival is above the median of the group receiving surgery + vehicle (60.5 days) (*n* = 8 surgery + vehicle and *n* = 11 surgery + DEBIO‐0932‐treated mice, 2 independent experiments). *P* value was calculated using two‐tailed *t*‐test.

Tumor reinitiation after surgery may involve similar mechanisms to those processes necessary during the early stages of brain colonization. Based on our data proving that DEBIO‐0932 effectively targets the early stages of metastasis *ex vivo* (Fig 1E–G) and that HSP90‐related genes are equally represented in relapsed metastases (Fig 5F), we hypothesized that DEBIO‐0932 could be used to prevent relapse. First, we validated the efficacy of DEBIO‐0932 to prevent metastasis initiation *in vivo* (Fig EV3A–G, Table EV10) using H2030‐BrM as a model following an angio‐co‐optive growth pattern during metastasis initiation (Valiente *et al*, 2014). Subsequently, we used the HSP90 inhibitor in an adjuvant setting after neurosurgery. Although DEBIO‐0932 administration at 160 mg/kg starting 3 days after surgery debuted with an initial impact limiting weight gain in mice, probably reflecting a more vulnerable health state post‐surgery, individualized systemic therapy (Fig EV3H) stabilized treated mice and delayed local relapse (Fig 5G–I). Remarkably, the percentage of mice surviving above the median increased to 82% with three animals showing at least a 20% extension in overall survival, one of them with stable disease over 18 weeks (Fig EV3I). However, survival benefits experienced by this arm did not reach statistical significance (Fig EV3I). Nonetheless, mice treated with adjuvant anti‐HSP90 therapy experienced a less aggressive relapse as measured by the reduced systemic impact (i.e., weight loss) of uncontrolled tumor growth in the brain (Fig 5J).

Our findings suggest that inhibition of HSP90 could be a novel strategy to prevent brain metastasis, including a clinically relevant situation of local relapse after neurosurgery.

### *In situ* proteomics uncovers HSP90‐dependent brain metastasis mediators

**Figure 6:**
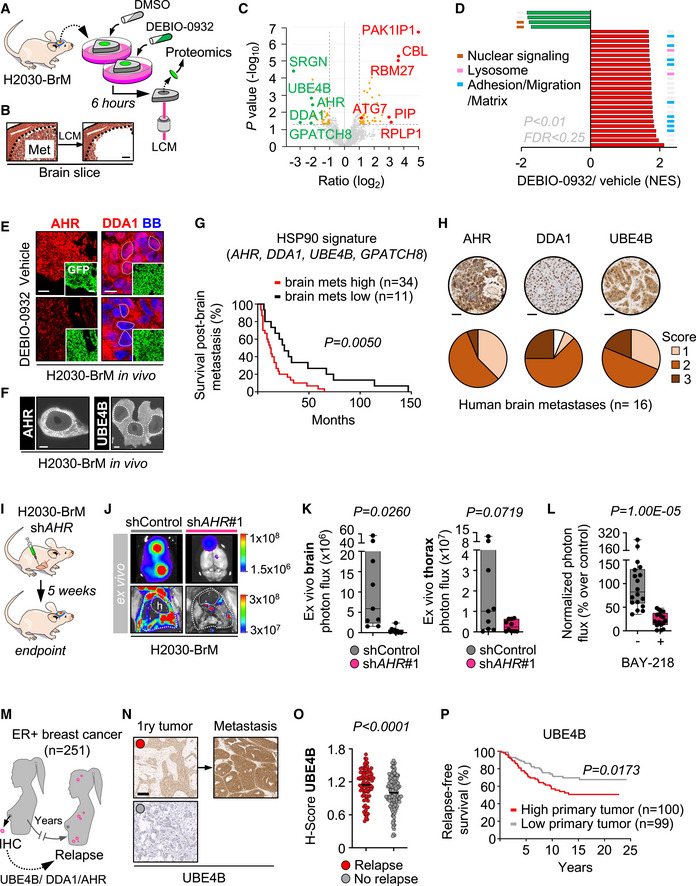
*In situ* proteomics uncovers HSP90‐dependent brain metastasis mediators Schema of experimental design. Organotypic cultures with established brain metastases from H2030‐BrM cells were treated with DEBIO‐0932 at 1 µM for 6 h and subjected to laser capture microdissection (LCM) and proteomic profiling.Representative image of a fully established brain metastasis from H2030‐BrM before and after laser capture microdissection (LCM). The dotted line delimits the metastasis. Scale bar: 100 µm.Volcano plot with deregulated proteins (red: upregulated; green: downregulated) found in brain metastases treated with DEBIO‐0932 compared to DMSO (*n* = 3 biological replicates (mice) per condition, *n* ≥ 12 brain metastases per mouse were pooled together). Proteins with a *P* < 0.05 and a log_2_ ratio > 1 or < −1 were defined as deregulated. Gray dotted lines indicate *P* value and log_2_ ratio cut offs. The names of the top deregulated proteins are shown.GSEA of top 25 upregulated (red) and downregulated (green; only four fulfill the filter) pathways upon DEBIO‐0932 treatment. Those biological processes represented with more than one signature are labeled with colored lines.Representative images showing AHR and DDA1 levels in brain metastases (generated by intracardiac inoculation of H2030‐BrM) found at endpoint of vehicle and DEBIO‐0932‐treated animals. This result was reproduced in 2 independent staining with different brains. BB: bisbenzamide. Scale bars: low magnification (HSP90 and GFP), 50 µm; high magnification (DDA1), 6 µm (dotted lines).Representative images of squash preparations showing nuclear AHR and UBE4B in established brain metastases from H2030‐BrM generated by intracardiac inoculation. Scale bar: 5 µm. The dashed line surrounds the nucleus.Kaplan–Meier curves showing significant correlation between worse survival post‐brain metastasis and high expression levels of the HSP90 signature (*AHR*, *DDA1*, *UBE4B*, *GPATCH8*) in a cohort of 45 breast cancer brain metastasis patients.Representative images (selected cases obtained from Fig EV6M) and histological score of AHR, DDA1 and UBE4B in human brain metastases (*n* = 16) according to the signal intensity of the corresponding protein in cancer cells.Schema of the experimental design. H2030‐BrM cells carrying the corresponding shRNA against *AHR* or the non‐targeting control were inoculated intracardially into nude mice. *Ex vivo* BLI of brains and thoracic regions were analyzed 5 weeks after injection of cancer cells. Brains were processed for histological analysis.Representative images of brains and thorax from shControl and sh*AHR*#1 mice at the endpoint of the experiment.Quantification of *ex vivo* BLI of brains and thoracic regions at the endpoint of the experiment. Values are shown in box‐and‐whisker plots where every dot represents a different animal and the line in the box corresponds to the median. The boxes go from the upper to the lower quartiles, and the whiskers go from the minimum to the maximum value (*n* = 9 shControl mice and *n* = 10 sh*AHR*#1 mice). *P* value was calculated using two‐tailed *t*‐test.Quantification of the bioluminescence signal emitted by H2030‐BrM established metastases in organotypic cultures at Day 7 normalized by the initial value obtained at Day 0 and normalized to the organotypic cultures treated with DMSO. Day 0 is considered right before addition of the treatment or DMSO. Values are shown in box‐and‐whisker plots where each dot is an organotypic culture and the line in the box corresponds to the median. The boxes go from the upper to the lower quartiles and the whiskers go from the minimum to the maximum value (*n* = 17 organotypic cultures treated with DMSO; *n* = 18 organotypic cultures treated with BAY‐218, 2 independent experiments). *P* value was calculated using two‐tailed *t*‐test.Schema depicting the evaluation of a clinical cohort composed of 251 ER^+^ breast cancer primary tumors with follow‐up to determine the correlation of UBE4B, DDA1 or AHR with relapse.Representative images of primary tumors with high (red dot) or low (gray dot) UBE4B levels. A few cases of matched primary metastases allowed to evaluate the HSP90‐dependent protein. Scale bar: 100 µm.H‐score analysis of UBE4B in primary tumors with (red) or without (gray) associated relapse. Values are shown in a scattered plot where each dot is a primary tumor and the line corresponds to the median (*n* = 100 primary tumors with relapse; *n* = 147 primary tumors without relapse). *P* value was calculated using two‐tailed *t*‐test.Kaplan–Meier curve comparing relapse‐free survival of primary tumors with high and low values of UBE4B. *P* value was calculated using log‐rank (Mantel‐Cox) test.

Our data support HSP90 as a therapeutic target in brain metastasis. Therefore, we wanted to investigate whether METPlatform could additionally contribute to characterize downstream mechanisms following target inactivation using unbiased approaches. To identify acute biological responses following HSP90 inhibition, we treated organotypic cultures containing established H2030‐BrM brain metastases with DEBIO‐0932 at 1 µM for 6 h. Laser capture microdissection of paraffin‐embedded metastatic lesions was followed by peptides identification by mass spectrometry (Fig 6A and B). Short time treatment with DEBIO‐0932 showed modest but statistically significant reduction of brain metastases as measured by BLI (Appendix Fig S2A), allowing us to assess early changes after HSP90 inhibition in cancer cells.

**Figure EV4:**
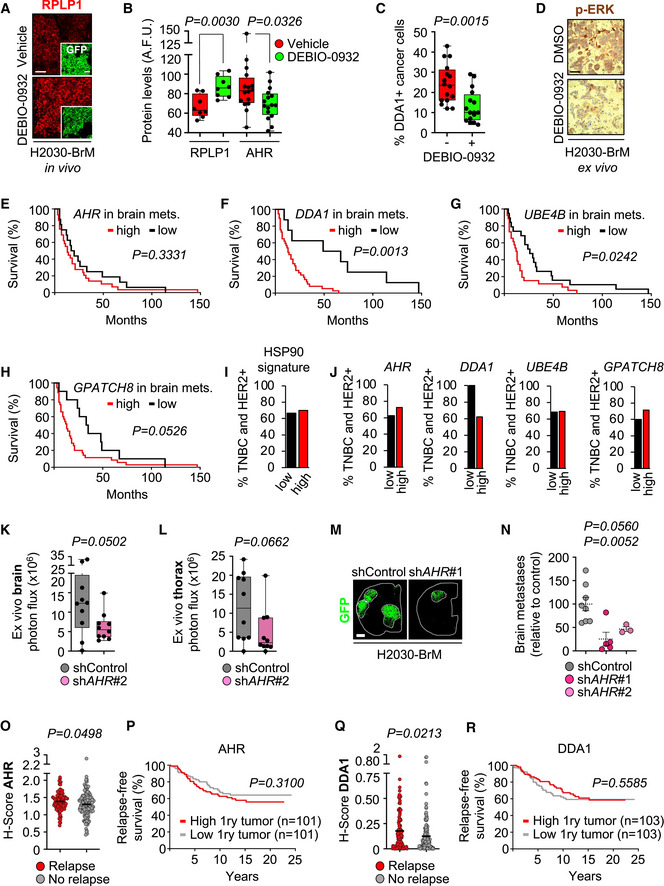
*In situ* proteomics uncovers HSP90‐dependent brain metastasis mediators ARepresentative images showing RPLP1 levels in brain metastases (generated by intracardiac inoculation of H2030‐BrM) found at endpoint of vehicle and DEBIO‐0932‐treated animals. This result was reproduced in two independent staining with different brains. Scale bars: 50 µm.BQuantification of RPLP1 and AHR levels shown in (Figs 6E and EV4A) in arbitrary fluorescent units (A.F.U.). Values are shown in box‐and‐whisker plots where each dot is a metastatic lesion and the line in the box corresponds to the median. The boxes go from the upper to the lower quartiles, and the whiskers go from the minimum to the maximum value (*n* = 8–16 metastatic lesions from 2 to 4 brains per condition, two independent staining with different brains were performed). *P* value was calculated using two‐tailed *t*‐test.CQuantification of percentage of nuclear DDA1^+^ BB^+^ cells shown in (Fig 6E). Values are shown in box‐and‐whisker plots where each dot is a metastatic lesion, and the line in the box corresponds to the median. The boxes go from the upper to the lower quartiles, and the whiskers go from the minimum to the maximum value (*n* = 16 metastatic lesions from 4 brains per condition, 2 independent staining with different brains were performed). *P* value was calculated using two‐tailed *t*‐test.DRepresentative images showing p‐ERK levels in organotypic cultures from (Fig 6A). This result was reproduced in three independent staining with organotypic cultures from different mice. Scale bar: 20 µm.E–HKaplan‐Meier curves showing significant correlation between worse survival post‐brain metastasis and high gene expression levels of *AHR* (E), *DDA1* (F), *UBE4B* (G), and *GPATCH8* (H) in a cohort of 45 breast cancer brain metastasis patients.I, JDistribution of poor prognosis breast cancer subtypes HER2^+^ and TNBC within the low and high gene expression level cohorts considering the signature (I) or individual genes (J).K, LQuantification of *ex vivo* BLI of brains (K) and thoracic regions (L) of mice inoculated with H2030‐BrM cells carrying shControl or sh*AHR*#2 at the endpoint of the experiment (5 weeks after injection of cancer cells). Values are shown in box‐and‐whisker plots where every dot represents a different animal and the line in the box corresponds to the median. The boxes go from the upper to the lower quartiles and the whiskers go from the minimum to the maximum value (*n* = 10 shControl mice and *n* = 10 sh*AHR*#2 mice). *P* value was calculated using two‐tailed *t*‐test.MRepresentative sections of brains from shControl and sh*AHR*#1 mice 5 weeks (experimental endpoint) after intracardiac inoculation of cancer cells. The dotted lines surround the metastases (GFP^+^). Scale bar: 1 mm.NQuantification of metastases found in brains inoculated with H2030‐BrM cells with sh*AHR*. Relative metastatic load was normalized to the respective control. Values are shown in dot plots where every dot represents a different brain and the dotted line corresponds to the mean ± s.e.m. (*n* = 8 shControl; *n* = 5 sh*AHR*#1; *n* = 3 sh*AHR*#2 mice). *P* value was calculated using two‐tailed *t*‐test.O, QH‐score analysis of AHR (O) and DDA1 (R) in primary tumors with (red) or without (gray) associated relapse. Values are shown in a scattered plot where each dot is a primary tumor and the line corresponds to the median (*n* = 100/103 primary tumors with relapse; *n* = 101/103 primary tumors without relapse, respectively). *P* value was calculated using two‐tailed *t*‐test.P, RKaplan‐Meier curve comparing relapse‐free survival of primary tumors with high and low values of AHR (P) and DDA1 (R). *P* value was calculated using log‐rank (Mantel‐Cox) test.

We identified 83 significantly deregulated proteins upon treatment with DEBIO‐0932, from which 44 were upregulated and 39 were downregulated (Fig 6C). We validated this analysis with immunofluorescence applied to brains from mice treated with DEBIO‐0932 *in vivo* to score top deregulated proteins (Figs 6E and EV4A–C).

Downregulated proteins upon DEBIO‐0932 treatment are potential HSP90‐dependent mediators of brain metastasis (Fig 6C). Interestingly, 50% of top downregulated signatures upon HSP90 inhibition belong to nuclear signaling pathways that include DNA‐binding proteins and transcription factor (Fig 6D, Table EV4, Appendix Fig S2B), and four out of five top downregulated proteins (AHR (aryl hydrocarbon receptor), DDA1 (DET1 and DDB1 associated 1), UBE4B (ubiquitination factor E4B), and GPATCH8 (G‐patch domain containing 8)) (Fig 6C) have been shown to be able to translocate into the nucleus (Fig 6E and F) (Murray *et al*, 2014; Du *et al*, 2016; Cheng *et al*, 2017). Within the nuclear compartment, the association of AHR and UBE4B with euchromatin reinforces the possibility of a functional role at this subcellular location (Fig 6F). In addition to previous findings (Fig 3J–L), our results suggest a prominent role for HSP90 or HSP90‐dependent downstream program in the nucleus of secondary brain tumors. Nonetheless, we do not rule out the impact of DEBIO‐0932 on cytoplasmic HSP90 clients, including cancer‐related kinases, in brain metastasis. In fact, a reduction in phosphorylated ERK1/2 (Thr202/Tyr204) was detected in organotypic cultures with established brain metastases treated with DEBIO‐0932 (Fig EV4D) in line with previously reported studies (Bao *et al*, 2009).

In patients, high versus low HSP90‐dependent four gene signature score (*AHR*, *DDA1*, *UBE4B,* and *GPATCH8*) in brain metastatic tumors associate with worse patient prognosis and aggressive clinical disease (Figs 6G and EV4E–H) in an extended cohort (GSE184869) of a previously published dataset of breast cancer patients (Varešlija *et al*, 2019) and independently of the cancer subtype (Fig EV4I and J). Additionally, AHR, DDA1, and UBE4B protein were detected in all tissue samples analyzed from an additional cohort of brain metastases independently of primary tumor source and the presence of clinically validated HSP90‐dependent oncogenes (Fig 6H, Appendix Fig S2C, Table EV5).

In order to evaluate the functional contribution of the HSP90‐dependent signature, we performed functional assays *in vivo*. Genetic knockdown identified *AHR* as functionally relevant in lung adenocarcinoma brain metastases (Figs 6I–K and EV4K–N). Similar to the pharmacological inhibition of HSP90 (Figs 4B–D and EV2F–H), loss of function of AHR also reduced extracranial metastases (Figs 6K and EV4L). A complementary pharmacologic approach (Fig 6L) confirmed that this novel brain metastasis mediator, part of the HSP90‐dependent signature of poor prognosis, should be also considered as a potential therapeutic target.

Although monogenic loss of function of *UBE4B* or *DDA1* did not allow us to conclude about their involvement in brain metastasis, we cannot discard that the trends observed *in vivo* (Appendix Fig S2D and E) would require an alternative approach targeting these candidates simultaneously. Indeed, the evaluation of an ER+ breast cancer cohort with a 10‐year follow‐up (Fig 6M, Table EV6), identified increased AHR, DDA1, and UBE4B levels in the primary tumors that relapsed (Figs 6O, EV4O and Q, Table EV7). The strong association seen with UBE4B was also predictive of relapse‐free survival (Fig 6P), while DDA1 and AHR failed to score in this analysis (Figs EV6P and Fig EV6R). Of note, within the 251 cases in this cohort only 3 relapses correspond to metastases in the brain (Table EV7), which reinforces the association of the HSP90‐dependent signature with multi‐organ metastases. Indeed, in a reduced number of eight matched samples including primaries that relapsed later on and their corresponding metastases (Table EV8) we observed that mean H score showed a trend to be higher in metastases than in the primary tumor (Table EV8).

Consequently, METPlatform could be coupled with unbiased omic approaches to provide a detailed molecular map of drug response *in situ*, which could facilitate the discovery of potential clinically relevant biomarkers.

### METPlatform facilitates unbiased identification of synergistic drug combinations against brain metastasis

**Figure EV5:**
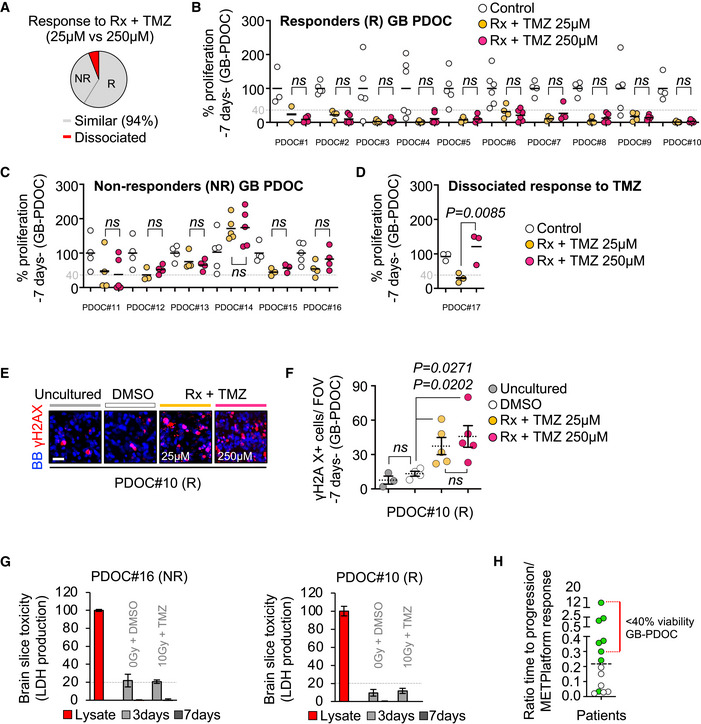
METPlatform as a clinically compatible “avatar” APie chart categorizing the 17 GB‐PDOC treated with radiation (Rx) and temozolomide (TMZ) into responders (R) and non‐responders (NR) independently of the dose of TMZ given (gray). Red area is labeling the single PDOC with a non‐homogeneous response to the two different doses of TMZ (D).B–DQuantification of the impact of Rx + TMZ on the proliferation of cancer cells from GB‐PDOC. Values are normalized to the respective control without treatment. Each dot represents an organotypic culture from each GB‐PDOC. GB‐PDOC was classified as responder when the mean (line in each dot cluster) is below 40%. *P* value was calculated using two‐tailed *t*‐test.ERepresentative images of a responder GB‐PDOC stained with a DNA‐damage marker, γ‐H2AX (red). BB: bisbenzamide. Scale bar: 25 µm.FQuantification of the number of γ‐H2AX^+^ cancer cells in the GB‐PDOC shown in (E). Each dot represents and individual organotypic culture where the mean number of cells positive for the DNA‐damage marker was measured. The dotted line represents the mean ± s.e.m. (*n* = 3, uncultured organotypic slices from the tumor; *n* = 4 GB‐PDOC treated with DMSO (1%); *n* = 5 GB‐PDOC treated with Rx + TMZ (25 μM); *n* = 5 GB‐PDOC treated with Rx + TMZ (250 μM)). *P* value was calculated using two‐tailed *t*‐test.GQuantification of LDH levels in the conditioned media of organotypic slices cultured during 3 and 7 days relative to a lysate of the same GB‐PDOC. Values are shown as mean + s.e.m. (*n* = 3 organotypic cultures per experimental condition, each graph correspond to an individual GB‐PDOC). LDH levels at 7 days measure the accumulation of the enzyme in the media from day 3 on.H
*Post hoc* quantification of the ratio between the number of months with stable disease in each patient (at cut off September 2021) and the corresponding % of proliferation after GB‐PDOC treatment in METPlatform. The dotted line indicates the median. Each dot corresponds to a patient. Green dots: patient with stable disease; white dots: patients with progressive disease. The red line labels those patients with a reduction above 40% in cancer cell proliferation in the corresponding GB‐PDOC.

Despite the encouraging pharmacological results obtained with DEBIO‐0932 *in vivo* (Figs 4B–G, EV2M–P, 5G–I, EV3A–F), control of metastatic disease could still be improved (Figs EV2L, EV2O, EV3G, and EV3I). Additionally, synergistic drug combinations are aimed to maximize sensitivity over tumor cells while minimizing toxicity in normal cells, which has been a limiting factor for the use of HSP90 inhibitors in patients (Neckers & Workman, 2012).

**Figure 7:**
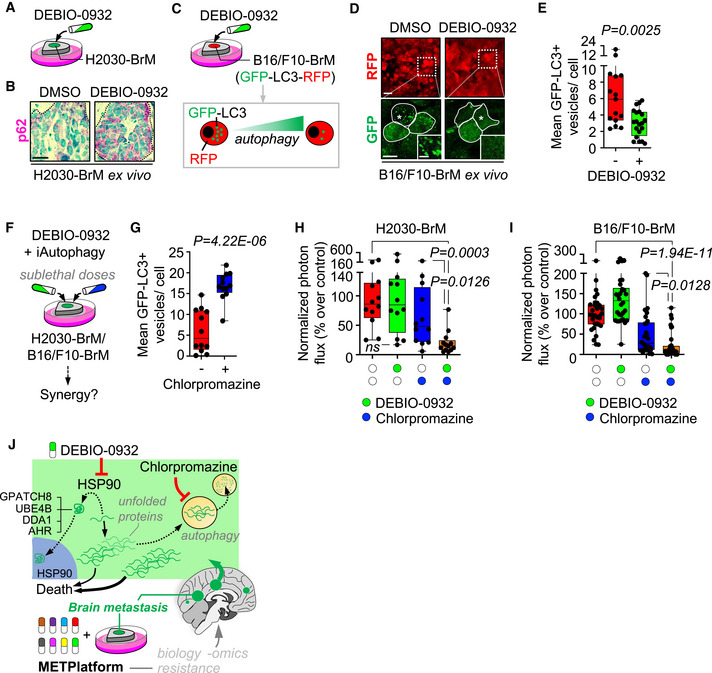
METPlatform facilitates unbiased identification of synergistic drug combinations against brain metastasis Schema of experimental design. Organotypic cultures with established brain metastases from H2030‐BrM cells were treated with DEBIO‐0932 and evaluated for p62 levels.Representative images showing p62 levels. This result was reproduced in three independent staining with organotypic cultures from different mice. Dotted lines delimit the metastasis. Scale bar: 10 µm.Schema of experimental design. Organotypic cultures with brain metastases from B16/F10‐BrM‐GFP‐LC3‐RFP cells were treated with DEBIO‐0932 and monitored for autophagic flux by GFP‐LC3^+^ puncta (vesicles).Representative organotypic cultures from the experiment in panel (C). RFP is an internal control probe labeling cancer cells independent of autophagy flux and GFP indicate GFP‐LC3^+^ puncta. The dotted line in the upper panel delimits a high magnification area shown in the lower panel respect to the GFP signal derived from GFP‐LC3 accumulation. Dotted lines in lower panel surround individual cancer cells. Asterisk labels the area in the cell magnified in the high magnification panel showing the GFP‐LC3^+^ puncta. Scale bar: low magnification, 25 µm; high magnification (cells), 10 µm; high magnification (puncta), 2.5 µm.Quantification of GFP‐LC3^+^ vesicles per cell of the experiment in panel (C). Values are shown in box‐and‐whisker plots where every dot represents a field of view of an organotypic culture and the line in the box corresponds to the median. The boxes go from the upper to the lower quartiles, and the whiskers go from the minimum to the maximum value (DMSO: *n* = 15 fields of view, 2,232 cancer cells from 3 organotypic cultures; DEBIO‐0932: *n* = 20 fields of view, 3,260 cancer cells from 4 organotypic cultures). *P* value was calculated using two‐tailed *t*‐test.Schema of experimental design. Organotypic cultures with established brain metastases were treated with DEBIO‐0932 and autophagy inhibitors at sublethal doses.Quantification of GFP‐LC3^+^ vesicles per cell in organotypic cultures with brain metastases from B16/F10‐BrM‐GFP‐LC3‐RFP cells treated with chlorpromazine (20 µM) and monitored for autophagic flux by GFP‐LC3^+^ puncta (vesicles). Values are shown in box‐and‐whisker plots where every dot represents a field of view of an organotypic culture and the line in the box corresponds to the median. The boxes go from the upper to the lower quartiles and the whiskers go from the minimum to the maximum value (DMSO: *n* = 12 fields of view, 1,919 cancer cells from 3 organotypic cultures; chlorpromazine: *n* = 12 fields of view, 1,759 cancer cells from 3 organotypic cultures). *P* value was calculated using two‐tailed *t*‐test.Quantification of the bioluminescence signal emitted by H2030‐BrM cells in each organotypic culture with established brain metastases at Day 3 normalized by the initial value at Day 0 (before the addition of any treatment; DEBIO‐0932 was added at 100 nM and chlorpromazine at 20 µM) and normalized to the organotypic cultures treated with DMSO. Values are shown in box‐and‐whisker plots where every dot represents an organotypic culture and the line in the box corresponds to the median. The boxes go from the upper to the lower quartiles and the whiskers go from the minimum to the maximum value (*n* = 12–13 organotypic cultures per experimental condition, 3 independent experiments). *P* value was calculated using two‐tailed *t*‐test.Quantification of the bioluminescence signal emitted by B16/F10‐BrM cells in each condition (DEBIO‐0932 was added at 100 nM and chlorpromazine at 15 µM) at Day 3 normalized by the initial value obtained at Day 0 and normalized to the organotypic cultures treated with DMSO. Day 0 is considered 12–16 h after the addition of B16/F10‐BrM cancer cells and treatment or DMSO. Values are shown in box‐and‐whisker plots where each dot is an organotypic culture and the line in the box corresponds to the median. The boxes go from the upper to the lower quartiles, and the whiskers go from the minimum to the maximum value (*n* = 30–33 organotypic cultures per experimental condition, 4 independent experiments). *P* value was calculated using two‐tailed *t*‐test.Graphical summary. METPlatform is a valuable tool for metastasis research that integrates drug‐screening and omic approaches to study pharmacological and biological vulnerabilities. We demonstrate that one vulnerability corresponds to the dependency on HSP90. The BBB‐permeable HSP90 inhibitor DEBIO‐0932 is an effective therapeutic strategy against established brain metastasis and the analysis of such phenotype with *in situ* proteomics revealed potential novel mediators of brain metastasis downstream HSP90. At the same time, autophagy appears as an actionable mechanism of resistance upon HSP90 inhibition, allowing design of rationale combinations using autophagy inhibitors and DEBIO‐0932 to target brain metastasis more effectively if appropriate drugs could be combined *in vivo*.

Our proteomic analysis on DEBIO‐0932 treatment identified the upregulation of multiple signatures representing adhesion, migration, and interaction with the matrix as well as increased lysosome activity (Fig 6D, Appendix Fig S2, Table EV4), all of which are known mechanisms involved in therapeutic resistance (Sui *et al*, 2013; Orgaz *et al*, 2020). Given that lysosome activity is tightly linked to autophagy (Sui *et al*, 2013) and previous studies reported the induction of autophagy by HSP90 inhibitors in cancer (Liu *et al*, 2012; Samarasinghe *et al*, 2014; Mori *et al*, 2015; He *et al*, 2016; Zhao *et al*, 2019), we decided to explore this process as a potential actionable resistance mechanism to HSP90 inhibition in brain metastasis. In addition to the upregulation of the autophagy‐related protein ATG7 (Levy *et al*, 2017) (Fig 6C), we noticed that the early response of cancer cells to HSP90 inhibition induced the accumulation of the adaptor protein p62 or sequestosome‐1 (Fig 7A and B). As an additional evidence of the molecular crosstalk between HSP90 and autophagy in brain metastasis, we used a probe that labels the flux of lysosomal degradation based on GFP‐tagged LC3 (Kaizuka *et al*, 2016). Given the unavailability of H2030‐BrM or MDA231‐BrM cell lines lacking the GFP reporter (Bos *et al*, 2009; Nguyen *et al*, 2009; Valiente *et al*, 2014; Chen *et al*, 2016; Valiente, 2020) and that DEBIO‐0932 efficacy on brain metastasis is independent of the primary source (Figs 1D, EV1B, EV1G, Table EV1, Figs 4J, EV2 O and P), we used the reporter‐free melanoma brain metastatic cell line B16/F10‐BrM (Priego *et al*, 2018) for this purpose (Fig 7C). Treatment of B16/F10‐BrM organotypic brain cultures with DEBIO‐0932 decreased the amount of GFP‐LC3^+^ vesicles, which indicates enhanced autophagic flux (Fig 7D and E). Of note, the same probe also encodes an autophagy‐independent RFP reporter, which does not change in the presence of DEBIO‐0932 (Fig 7D). Based on the above findings indicating increased autophagy upon DEBIO‐0932 treatment, we combined it with the broadly used autophagy inhibitor bafilomycin A1 (Mauvezin *et al*, 2015). Combined therapy with both inhibitors in established lung adenocarcinoma H2030‐BrM brain metastases *ex vivo* showed synergistic effects compared to sublethal concentration of DEBIO‐0932 (Appendix Fig S3). However, bafilomycin A1 did not progress to clinical development due to its poor toxicity profile *in vivo* causing disturbances in locomotor control and convulsions (Keeling *et al*, 1997; DeVorkin & Lum, 2014). Therefore, we looked for alternative compounds able to block autophagy and superior ability to cross the BBB. The FDA‐approved anti‐psychotic drug chlorpromazine fulfills these two requirements (Fig 7G) (Nadanaciva *et al*, 2011). As predicted based on our findings, the combination of sublethal concentration of DEBIO‐0932 with the CNS‐related drug chlorpromazine (Fig 7F) was effective against H2030‐BrM (Fig 7H) as well as B16/F10‐BrM (Fig 7I) brain metastases *ex vivo*. However, translation of this combination therapy *in vivo* has not been successful (Appendix Fig S3G) potentially derived from the secondary effects (i.e., long‐term drowsiness accompanied with weight loss) (Appendix Fig S3C and D) of chlorpromazine at the dose required to detect brain levels (Appendix Fig S3I) and its negative impact, even at lower concentration used (5 mg/kg) on decreasing the accumulation of DEBIO‐0932 in the brain (Appendix Fig S3H). In an effort to evaluate an alternative member of the same class of autophagy inhibitors, trifluoperazine was used (Xia *et al*, 2021). However, similar *in vivo* findings emerged including the need to limit its dose because of toxic effects when combined (Appendix Fig S3E and F) as well as a similar dramatic reduction of DEBIO‐0932 accumulation in the brain (Appendix Fig S3H). In this case, even though the dose of trifluoperazine had to be reduced early on (Appendix Fig S3E and F), the compound was still detected in the brain (Appendix Fig S3J).

Thus, the use of METPlatform to identify combination strategies that could be more effective against metastasis also involves its limitation to predict crucial pharmacokinetic aspects at the organismal level. Consequently, METPlatform must be conceived as a strategy to facilitate the initial testing of novel concepts for drug repurposing (i.e., the use of an anti‐psychotic drug to compromise brain metastasis) (Fig 7J), favoring the reduction of the use of animal models rather than the replacement of *in vivo* validation, a crucial step to define the viability of a therapeutic strategy.

### METPlatform as a clinically compatible “avatar”

**Figure 8:**
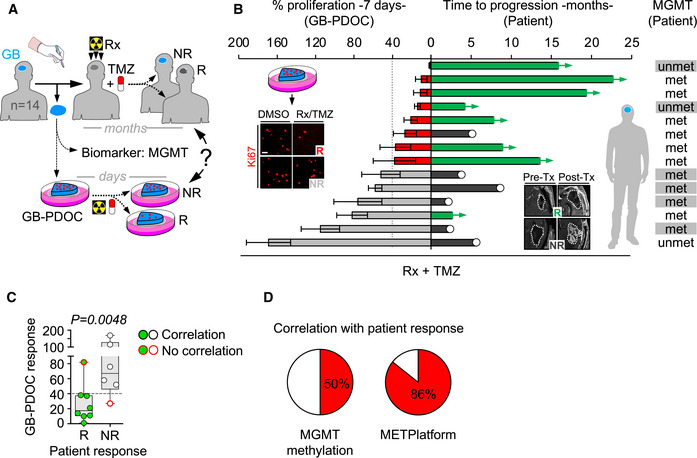
METPlatform as a clinically compatible “avatar” Schema of experimental design using GB‐PDOC.Correlation between response in GB‐PDOC (left side of the graph) and its respective patient (right side of the graph). Response in GB‐PDOCs was obtained by quantification of number of proliferative cancer cells found in Rx + TMZ treated normalized to DMSO‐treated (100%) PDOCs from the same patient. Representative GB‐PDOC responding (R) or not (NR) to the standard of care that was provided *ex vivo* (Radiation (Rx): 2 × 10 Gy + temozolomide (TMZ) 250 µM) are shown. Scale bar: 50 µm. Values are shown as mean ± s.e.m. (*n* = 3–6 PDOCs per experimental condition per patient, each patient is represented in an individual bar. Fourteen patients included; each patient is an independent experiment). Time to progression of the patients after neurosurgery is represented in months. Response to Rx + TMZ was evaluated by volumetric measurement of the lesion (dashed line) based on MRI before and after the treatment. Representative patients responding (R) or not (NR) to the standard of care are shown. A white circle indicates progressive disease. Patients with ongoing response to Rx + TMZ (stable disease) are indicated with green bars. MGMT promoter methylation status is shown for each patient (met: methylated; unmet: unmethylated). N/A: not available.Correlation between GB‐PDOC and patient responses. GB‐PDOC response is indicated by the mean value in percentage of proliferation post‐treatment, where 40% represents the threshold. Patients are classified as responder (R) (green dots, 8 patients) or non‐responder (NR) (white dots, 6 patients) according to the MRI evaluation. Values are shown in box‐and‐whisker plots where each dot is a patient and the corresponding GB‐PDOC and the line in the box corresponds to the median. The boxes go from the upper to the lower quartiles and the whiskers go from the minimum to the maximum value. *P* value was calculated using two‐tailed *t*‐test.Pie charts representing the percentage of patients where MGMT methylation status correlates with the expected therapeutic response in the patient and the same respect to the response of the GB‐PCOC in METPlatform.

A major benefit of METPlatform would derive from its use as a strategy for personalized medicine, for instance by providing a fast readout on the efficacy of postsurgical adjuvant treatments. To evaluate PDOC as *ex vivo* “avatars” of cancer patients, we performed a proof‐of‐concept substantiation with glioblastoma (GB) diagnosed *de novo*. In contrast to the lack of standard of care after neurosurgery in patients with brain metastasis, those with GB invariably receive radiotherapy plus temozolomide (Fig 8A) (Stupp *et al*, 2005, 2009). We initially tested the combination of radiation and temozolomide in 17 glioblastoma patient‐derived organotypic cultures (GB‐PDOC) to demonstrate the existence of responders (R) (Fig EV5B) and non‐responders (NR) (Fig EV5C) to the standard of care, as well as the consistency of such category (R vs. NR) in a broad concentration range of the alkylating agent (Fig EV5A–D, Table EV10). Additionally, we confirmed that the decrease in proliferation (Fig EV5B) matched the sustained induction of DNA damage upon treatment (Fig EV5E and F), thus confirming this readout to asses therapeutic response to the combination therapy, as previously reported (Oldrini *et al*, 2020). Finally, we evaluated that GB‐PDOC did not experience a different degree of compromised viability compared to our previous results (Fig 2D, Appendix Fig S1) with this unspecific assay (Fig EV5G).

In order to evaluate whether GB‐PDOC recapitulate patient response to radiotherapy and temozolomide, we compared the response of fourteen GB‐PDOC treated with the standard of care during seven days with the clinical response of matched patients at initial MRI post‐therapy. In addition, we evaluated the MGMT methylation status, an established predictive biomarker of response to this therapy (Hegi *et al*, 2005; Stupp *et al*, 2005, 2009) (Fig 8A and B, Table EV6). GB‐PDOC showing cancer cell proliferation under 40% as compared to the corresponding untreated PDOC from the same patient were considered responders (Fig 8B). At data cutoff in September 2021, all patients had been evaluated for response to the standard of care and were classified as progressive (non‐responder, NR) or stable disease (responder, R), with the last ones showing ongoing response at time of cutoff (Fig 8B, Table EV9). Remarkably, GB‐PDOC predicted clinical response of their respective patient in 86% of the cases (Fig 8B–D, Table EV9). In contrast, MGMT methylation status correlated with patient response in 50% of the cases (Fig 8B and D, Table EV9). *Post hoc* data analysis at cutoff defined that a ratio above 0.2 between the time of sustained patient response and the percentage of proliferation in treated GB‐PDOC defined the category of stable disease in 87% of the patients (7/8 patients with stable disease) (Fig EV5H, Table EV9). Interestingly, 86% (6/7) of the patients with a ratio above 0.2 have less than 40% of proliferative cancer cells in their matched GB‐PDOC (Fig EV5H, Table EV9), confirming its relevance to define response or lack of thereof in this cohort of patients.

Consequently, this preliminary finding suggests that METPlatform might have potential predictive value as a clinically compatible patient “avatar”. However, in order to conclude about METPlatform as a realistic strategy to improve personalized cancer care the current limited data must be significantly expanded to additional clinical cohorts.

## Discussion

The novel drug‐screening platform we report here (METPlatform) allows identification of pharmacological vulnerabilities of metastasis *in situ* using an *ex vivo* setting. Organotypic cultures is a well‐established technique that could be applied to many different organs (Shamir & Ewald, 2014; Humpel, 2015) and has been reported by us and others to maintain the specific tissue architecture and cellular composition when applied to metastasis (Valiente *et al*, 2014; Er *et al*, 2018; Priego *et al*, 2018; Zhu & Valiente, 2021). Although organotypic cultures do have important limitations as an experimental drug‐screening platform for metastasis since they do not mimic the whole metastatic cascade, do not have the ability to score whether a compound crosses a vascular barrier such as the BBB or blood–tumor barrier (BTB) in the brain, and do not provide PK/PD information besides the organ under evaluation, this strategy is still highly versatile compared to other methods. For instance, METPlatform is able to resemble different stages of organ colonization and allows evaluating the impact of drugs on the non‐cancer compartment, and it is compatible with omic approaches, therefore providing a unique tool for basic and translational metastasis research.

The exclusion of patients with brain metastasis from clinical trials together with the limited information on the ability of drugs to cross the BBB or BTB constitute a vicious cycle that decelerates the development of new therapeutic opportunities for patients with brain metastases. METPlatform might be a valid strategy to challenge this situation by providing a relevant tissue context where drugs never used for brain metastases could be easily tested and, if appropriate, prioritized for further *in vivo* validation.

The unique ability of METPlatform to identify hits scoring *ex vivo* but not *in vitro* is of particular interest since their targets (HSP90, MEK1/2, CDKs, RAF1, BRAF, VEGFR, PDGFR, FGFR, DNA‐PK, PI3K, ATR, c‐Kit) might be key during organ colonization. Adaptation of cancer cells to a new organ involves molecular changes in the transcriptome, metabolome, or proteome (Park *et al*, 2011; Sevenich *et al*, 2014; Basnet *et al*, 2019). These changes occurring *in situ* (i.e., metastatic cells colonizing the brain) could underlie the induction of a drug target or the activation of a resistance mechanism (Chen *et al*, 2016). Even more, METPlatform allows to uncover specific molecular vulnerabilities of the different compartments within the biology of organ colonization. For instance, inhibitors that are only effective at advanced stages of the disease might reflect the remodeling of the naïve microenvironment into a protumoral niche (Sevenich *et al*, 2014; Priego *et al*, 2018) where drug targets could be also present within the non‐cancer compartment (i.e., pazopanib, #110) (Gril *et al*, 2013).

To validate the vulnerabilities identified by METPlatform, we proved the dependency of brain metastasis on HSP90 signaling *in vivo*. Although the association of high HSP90 levels with systemic disease in cancer has been broadly reported (Pick *et al*, 2007; Gallegos Ruiz *et al*, 2008; McCarthy *et al*, 2008; Su *et al*, 2016; Dimas *et al*, 2018), whether this association involves any specific step of the metastatic cascade had not been addressed. We demonstrate that the last step of metastasis, organ colonization, is sensitive to HSP90 inhibition, either during the initial stages as well as once metastases are established. Interestingly, we identified HSP90‐dependent proteins AHR, DDA1 UBE4B, and GPATCH8, which share the ability to translocate to the nucleus (Murray *et al*, 2014; Du *et al*, 2016; Cheng *et al*, 2017), as functionally relevant in brain metastasis and/or present in patient samples from a variety of primary tumors. Given the evident heterogeneity among BrM‐PDOC regarding their response to DEBIO‐0932 at low concentrations and the lack of a clear correlation with HSP90‐dependent oncogenes, it remains to be determined whether the HSP90 signature could predict the response rate to the drug. All these findings strongly suggest the discovery of a novel molecular program in organ colonization during metastasis, which can be explored therapeutically beyond HSP90 inhibitors (i.e., AHR inhibitor). Although we focused our efforts on brain metastasis, the dependency on HSP90 signaling does not support an organ‐specific model as suggested by functional experiments and association with extracranial relapse in patients. In this sense, the identification of Iba1^+^/HSP90^+^ tumor‐associated microglia/macrophages deserve further research to define whether or not they contribute to metastasis progression and if so whether they do in an organ‐dependent manner.

DEBIO‐0932, a second‐generation inhibitor of HSP90 with the ability to penetrate the BBB (Bao *et al*, 2009), is currently under evaluation in a Phase I/II study for patients with advanced NSCLC (NCT01714037). Reports on selected patients enrolled in this trial suggest potential benefit in the metastatic setting (Cedrés *et al*, 2018). To the best of our knowledge, none of the clinical trials using HSP90 inhibitors includes patients with brain metastases. Therefore, our work provides the rationale and proof‐of‐principle to include patients with CNS disease in current clinical trials with BBB‐permeable HSP90 inhibitors and/or to design a specific one for this patient population in the adjuvant setting after neurosurgery. Of note, although our extensive evaluation of DEBIO‐0932 regarding its potential toxic effects did not report any major finding, we encourage that specific preclinical analysis taking into account the expression of HSP90 in specific brain neuronal nuclei will take place before any further clinical consideration.

The model of local relapse after neurosurgery that we report here represents not only a clear opportunity to translate our findings into a clinically compatible preventive strategy, but also the opportunity to investigate the biology of this process for the first time. Indeed, the molecular characterization performed suggests the provoking hypothesis that cancer cells left behind after a debulking neurosurgery reinitiate metastasis using vascular co‐option, a key mechanism during the early times of organ colonization (García‐Gómez & Valiente, 2020). However, in contrast to the initial colonization of the brain, local relapse post‐surgery will require the additional ability of metastasis‐initiating cells to cope with a severely damaged microenvironment to regenerate the tumor.

The highest potential of METPlatform stands on its clinical compatibility. Patient “avatars” have been exploited using patient‐derived xenografts (PDX), patient‐derived organoids (PDO) and patient‐derived primary cultures (PDC) to test candidate drugs prior to the treatment of the patient, therefore helping to select empirical therapies for the study (Garralda *et al*, 2014; Gao *et al*, 2015; Lee *et al*, 2018; Vlachogiannis *et al*, 2018; Jiang *et al*, 2020). However, these models frequently dissociate from the limited time available in the clinic, involve a significant source of variability and technical requirements to be established, and lack the microenvironment, all of which become important limitations to their translation into a clinical scenario. In this regard, we show the potential of METPlatform to outperform established clinical biomarkers to predict therapeutic response in a clinically compatible time frame when applied to a difficult‐to‐treat cancer (i.e., glioblastoma).

Predicting patient response to a specific treatment is highly desirable in the current context of personalized medicine. METPlatform could be explored to test experimental therapies *ex vivo*, as monotherapies or in combination with the standard of care, in preparation for the potential emergence of resistance in patients. In addition, PDOC that do not respond to the standard of care (*ex vivo* and in the clinic) but are sensitive to a given experimental therapy could be fully characterized with multiomic profiling in the search for novel biomarkers of the identified vulnerabilities. We are confident that METPlatform could contribute to achieve optimal patient stratification toward improved clinical trial design, thus favoring the impact of personalized medicine in Oncology. In particular, we propose to further characterize it on patients at high risk of developing resistance or when no reliable biomarker‐driven therapies exist, which are both aspects of especial relevance in metastasis.

## Materials and Methods

### Chemicals and reagents

An in‐house chemical library composed of 114 FDA‐approved or in clinical trials anti‐tumoral drugs (Bejarano *et al*, 2019) solved in DMSO (Sigma‐Aldrich) was used for *ex vivo* and *in vitro* screening. For *in vivo* treatment, DEBIO‐0932 (MedChemExpress) was formulated in 30% captisol (Ligand), chlorpromazine hydrochloride (Abcr) was solved in 0.9% saline solution and trifluoperazine hydrochloride (Merck) was formulated in 12.5% cremophor, 2.5% DMSO, and 85% saline. For *ex vivo* and *in vitro* treatments, DEBIO‐0932, methotrexate (MedChemExpress), bafilomycin A1 (Selleckchem), chlorpromazine (Sigma‐Aldrich), and BAY‐218 (Selleckchem) were solved in DMSO. CyQUANT™ LDH Cytotoxicity Assay (C20301, Thermo Fisher) was used following manufacturer’s instructions. CyQUANT™ LDH Cytotoxicity Assay (C20301, Thermo Fisher) was used following manufacturer’s instructions.

### Animal studies

All animal experiments were performed in accordance with a protocol approved by the CNIO (IACUC.030‐2015), Instituto de Salud Carlos III (CBA35_2015‐v2), and Comunidad de Madrid Institutional Animal Care and Use Committee (PROEX250/15 and PROEX135/19). Females athymic nu/nu (Harlan) or equal proportions of C57BL/6 males and females mice (for the B16/F10‐BrM model) 4–10 weeks of age were used. Housing and husbandry conditions are in accredited by AAALAC. Mice are SPF with microbiological and environmental parameters constantly monitored. Brain colonization assays were performed by injecting 100 μl PBS into the left ventricle containing 100,000 or 40,000 cancer cells or 2 μl RPMI1640 intracranially (the right frontal cortex, approximately 1.5 mm lateral and 1 mm caudal flow bregma, and to a depth of 1 mm) containing 100,000 cancer cells by using a gas‐tight Hamilton syringe and a stereotactic apparatus. Brain colonization was analyzed *in vivo* and *ex vivo* by BLI. Anesthetized mice (isoflurane) were injected retro‐orbitally with D‐luciferin (150 mg/kg; Syd Labs) and imaged with an IVIS machine (Perkin Elmer). Bioluminescence analysis was performed using Living Image software, version 4.5. Brain tumor resection was performed by adapting previously described procedures (Morrissy *et al*, 2016). In brief, after exposing the skull, a craniotomy is performed surrounding the tumor area, which is visualized by GFP, using an excitation light source at 460–495 nm (FS/ULS‐02 B2, BLS Ltd) and goggles carrying emission filters (FHS/EF‐3GY1, BLS Ltd). The skull and the dura are lifted with micro‐dissecting forceps, and the bulk of the tumor is then removed using a microcurette guided by GFP. When hemostasis is obtained, the surgical wound is sutured using interrupted stitching with absorbable sutures. Animals receive meloxicam at 5 mg/kg once per day during 72 h and dexamethasone at 13 mg/kg once per day during 48 h to contain brain edema. DEBIO‐0932 was administered by oral gavage (160 mg/kg) for 3 weeks, daily during the first week and once every 48 h during the two following weeks, starting 7 or 14 days after intracardiac inoculation of H2030‐BrM for preventive or interventive therapy, respectively. For preventive therapy of relapse after neurosurgery, DEBIO‐0932 was administered by oral gavage (160 mg/kg) for 5–6 weeks, starting 3 days after neurosurgery. Treatment was given in an individualized regimen according to clinical symptoms of toxicity, including mouse weight, diarrhea, and activity of the animal. For combination therapy of DEBIO‐0932 with autophagy inhibitors, DEBIO‐0932 was administered following the interventive setting, and chlorpromazine at 5 mg/kg or trifluoperazine at 10 mg/kg was administered daily intraperitoneally for 3 weeks, starting at 14 days after intracardiac inoculation of H2030‐BrM cells. For interventive therapy of DEBIO‐0932 (160 mg/kg) in B16/F10‐BrM tumors, treatment was given daily for 10 days starting at 3 days after intracranial inoculation of cancer cells.

### Organotypic cultures

Organotypic cultures from adult mouse brain and liver were prepared as previously described (Valiente *et al*, 2014). Brains with established metastases (5–7 weeks after intracardiac inoculation of cancer cells) or without metastases (wild‐type) and wild‐type livers were used. In brief, organs were dissected in HBSS supplemented with HEPES (pH 7.4, 2.5 mM), D‐glucose (30 mM), CaCl_2_ (1 mM), MgCl_2_ (1 mM), and NaHCO_3_ (4 mM) and embedded in 4% low‐melting agarose (Lonza) preheated at 42°C. The embedded organs were cut into 250‐μm slices using a vibratome (Leica). Brain slices were divided at the hemisphere into two pieces. Slices were placed with flat spatulas on top of 0.8‐μm pore membranes (Sigma‐Aldrich) floating on slice culture media (DMEM, supplemented HBSS, FBS 5%, l‐glutamine (1 mM), and 100 IU/ml penicillin/streptomycin). Brain slices were imaged to confirm the presence of established metastases using BLI (day 0) and were cultured in the presence of the anti‐tumoral library at 10 µM of each compound. Brain slices were imaged 3 days after the addition of the inhibitors (day 3). For 7 days cultures, treatments were replaced at day 3 in fresh media and slices were imaged at day 7. If slices were obtained from wild‐type brains, 30,000 cancer cells suspended in 2 μl of slice culture media were placed on the surface of the slice and incubated in the presence of the inhibitors for 4 days. Brain slices were imaged 12–16 h after addition of cells (Day 0) and 3 days after the first BLI (Day 3). Growth rate was obtained by comparing the fold increases between Day 3/7 and Day 0, and normalized to values obtained from slices cultured with DMSO (100%). The BrdU pulse (0.2 mg/ml, Sigma‐Aldrich) was given by adding it in the media 2 h (H2030‐BrM) or 4 h (MDA231‐BrM) before fixation. Brain slices were fixed in 4% paraformaldehyde (PFA) overnight and then free‐floating immunofluorescence was performed. For proteomic analysis, organotypic cultures with established brain metastases from H2030‐BrM were treated with DEBIO‐0932 at 1 µM for 6 h followed by fixation with 4% PFA overnight at 4°C. For analysis of autophagic flux, 200,000 B16/F10‐BrM cells with stable expression the autophagy probe GFP‐LC3‐RFP were added on wild‐type brain slices and incubated for 24 h, followed by DEBIO‐0932 treatment at 10 µM for 12 h and fixation with 4% PFA overnight at 4°C. For evaluation of hepatotoxicity, wild‐type liver slices were cultured in the presence of the corresponding inhibitors for 3 days and fixed with 4% PFA overnight at 4°C followed by free‐floating immunofluorescence.

### Patient‐derived organotypic cultures (PDOC)

Surgically resected human brain metastases and newly diagnosed glioblastomas were collected in neurobasal media A supplemented with 1 × B27 (17504‐044, Gibco), 1 × N‐2 (17502‐048, Gibco), 25 ng/ml bFGF (13256029, Gibco), 100 ng/ml IGF1 (291‐G1, R&D Systems), 25 ng/ml EGF (E9644, Sigma‐Aldrich), 10 ng/ml NRG1‐β1/HRG‐β1 (396‐HB, R&D Systems), 100 IU/ml penicillin/streptomycin, and 1 µg/ml amphotericin B. Tissue was embedded in 4% low‐melting agarose, and 250‐µm slices were obtained using a vibratome. Slices from brain metastases were cultured in the presence of DEBIO‐0932 at 10 µM and 1 µM for 3 days. Slices from glioblastomas were cultured in the presence of temozolomide (Sigma‐Aldrich) at 250 µM for 8 days and received 20 Gy of radiation fractionated in 2 doses of 10 Gy at days 1 and 4. Temozolomide treatment was replaced at Day 4 in fresh media. A BrdU pulse (4 h) was given at the end of the experiment followed by fixation of slices with 4% PFA overnight at 4°C and free‐floating immunofluorescence. Informed consent was obtained from all subjects. The experiments are aligned to the principles set out in the WMA Declaration of Helsinki and the Department of Health and Human Services Belmont Report. All samples were in compliance with protocols approved by their respective Institutional Review Board (IRB) (CEI.PI.64_2016‐v3, CEI PI 25_2020‐v2, CEI PI 50_2021).

### Cell culture

Human and mouse BrM cell lines have been previously described and obtained from the same batches that were previously published (Bos *et al*, 2009; Nguyen *et al*, 2009; Valiente *et al*, 2014; Priego *et al*, 2018). Cell lines were validated by morphological analysis and their behavior *in vivo*. All cell lines were tested mycoplasma‐free. H2030‐BrM3 (abbreviated as H2030‐BrM) (Origin: Massagué lab, MSKCC) and PC9‐BrM3 (abbreviated as PC9‐BrM) (Origin: Massagué lab, MSKCC) were cultured in RPMI1640 media supplemented with 10% FBS, 2 mM l‐glutamine, 100 IU/ml penicillin/streptomycin, and 1 µg/ml amphotericin B. MDA231‐BrM2 (abbreviated as MDA231‐BrM) (Origin: Massagué lab, MSKCC), ErbB2‐BrM2 (abbreviated as ErbB2‐BrM) (Origin: Massagué lab, MSKCC), 393N1 (Origin: Massagué lab, MSKCC), and B16/F10‐BrM3 (abbreviated as B16/F10‐BrM) (Origin: Valiente Lab, CNIO) where cultured in DMEM media supplemented with 10% FBS, 2 mM l‐glutamine, 100 IU/ml penicillin/streptomycin, and 1 µg/ml amphotericin B. For retrovirus production, HEK293T cells were cultured in DMEM media supplemented with 10% FBS, 2 mM l‐glutamine, 100 IU/ml penicillin/streptomycin, and 1 µg/ml amphotericin B. For the *in vitro* screening with the anti‐tumoral library, H2030‐BrM cells were seeded in 96‐well microtiter plates at a density of 4,000 cells/well. Cells were incubated for 24 h before adding the compounds. Compounds were weighed out and solved in DMSO to a final concentration of 10 mM. From here, a “mother plate” with serial dilutions was prepared at 100× the final concentration in the culture. The final concentration of DMSO in the tissue culture media should not exceed 1%. 2 µl of the compounds were added automatically (Beckman FX 96 tip) to 200 µl media to make it up to the final concentration for each drug. Each concentration was assayed in duplicate. Cells were exposed to the compounds for 72 h and then processed for CellTiter‐Glo^®^ Luminescent Cell Viability Assay (Promega) readout according to manufacturer’s instructions and read on EndVision (Perkin Elmer). Proliferation rate (%) was calculated by normalizing luminescent values obtained for each compound to values obtained with DMSO (100%). For oncosphere generation, 1,000 H2030‐BrM cells were plated in low‐attachment plates in Humec medium (12753018, Gibco) supplemented with 1× B27, 10 ng/ml bFGF, 20 ng/ml EGF, and 5 µg/ml insulin solution from bovine pancreas (IGF1; I0516, Sigma‐Aldrich). Cells were grown for 14 days until oncospheres were formed, followed by treatment compounds from the anti‐tumoral library for 3 days. Growth rate was obtained by comparing the fold increases in BLI between Day 3 and Day 0, and normalized to values obtained from oncospheres cultured with DMSO (100%).

### Virus production

For lentivirus production, HEK293T cells at 70% confluence were transfected in Opti‐MEM with Lipofectamine 2000 (Invitrogen) and incubated at 37°C overnight with 8.75 µg of the following plasmids: pMDLg/pRRE (#12251, Addgene), pRSV‐Rev (#12253, Addgene), VSV.G (#14888, Addgene), and lentiviral vectors carrying the corresponding shRNA against human AHR (sh#1: clone ID TRC21258, ATTAAGTCGGTCTCTATGCCG; sh#2: clone ID TRC21254, AAGACATTATATTGTTGTGGG; Horizon Discovery), human UBE4B (sh#1: clone ID TRC7546, TTAAAGGCAGTGTTATATCGG; sh#2: clone ID TRC7548, TATTGTGGATTTGATCCCTGC; Horizon Discovery), human DDA1 (sh#1: clone ID TRC142338, TCACGATGATCTGTTCAGACG; Horizon Discovery), or non‐targeting control. For retrovirus production, HEK293T cells at 70% confluence were transfected with 10 µg of pMRX‐IP‐GFP‐LC3‐RFP (#84573, Addgene), 5 µg of VSV.G (#14888, Addgene), and 5 µg pCL‐Eco (#12371, Addgene) in Opti‐MEM with Lipofectamine 2000 (Invitrogen) and incubated at 37°C for 8 h. For both types of viruses, media was replaced with DMEM supplemented with 10% FBS and 2 mM l‐glutamine and virus production was maintained for 48 h. Viral supernatant was collected, passed through a 0.45‐µm syringe filter and added to the corresponding cells (lentivirus to H2030‐BrM and retrovirus to B16/F10‐BrM) at 50% confluence in RPMI1640 (H2030‐BrM) or DMEM (B16/F10‐BrM) supplemented with 10% FBS, 2 mM l‐glutamine, and polybrene (5 µg/ml, Sigma‐Aldrich). The following day, media was replaced with the respective culture media. Selection with puromycin (2 µg/ml, Sigma‐Aldrich) was started 48 h after and maintained until complete cell death was observed in the non‐infected cancer cells.

### Clinical samples and immunohistochemistry

Sixty brain metastases from lung cancer (40 cases) and breast cancer (20 cases) and thirty matched primary tumors (28 lung tumors and 2 breast tumors) were obtained from University Hospital of Turin to assess HSP90 levels. Twenty‐two brain metastases were obtained from Hospital Universitario 12 de Octubre to evaluate UBE4B, AHR, ALK, ROS1, HER2, and BRAF^V600E^ status. All samples followed protocols approved by the corresponding IRB (1‐18‐01/2016 and CEI.PI.64_2016‐v3) and the Biobank of the hospital, respectively. Immunohistochemistry against HSP90, DDA1, UBE4B, or AHR was performed at the CNIO Histopathology Core Facility using a standardized automated protocol (Ventana Discovery XT, Roche for HSP90; AS Link, Dako, Agilent for DDA1, UBE4B and AHR). All reagents, with exception of the primary antibodies, were purchased from Roche and Agilent. For HSP90, antigen retrieval was performed using cell conditioning solution (CC1 mild), followed by endogenous peroxides blocking with inhibitor CM. Antigen retrieval was performed with high pH buffer for AHR and low pH buffer for DDA1 and UBE4B, and endogenous peroxidase was blocked with peroxide hydrogen at 3%. Slides were incubated with the corresponding primary antibodies as follows: HSP90α/β (clone F‐8, 1:3,000; sc‐13119, Santa Cruz Biotechnology, 8 min), DDA1 (1:2,400; 14995‐1‐AP, ProteinTech), UBE4B (1:500; ab97697, Abcam) and AHR (1:400; 031714, USBiologicals Life Sciences). After the primary antibody, slides were incubated with the corresponding secondary antibodies and visualization systems (OmniMap for HSP90 and EnVision FLEX + Rabbit Linker for DDA1, UBE4B, and AHR) conjugated with horseradish peroxidase. Immunohistochemical reaction was developed using ChromoMap DAB kit. Nuclei were counterstained with hematoxylin. Finally, the slides were dehydrated, cleared, and mounted with a permanent mounting medium for microscopic evaluation. Positive control sections known to be primary antibody positive were included for each staining run. Immunostains were blindly evaluated and scored by a pathologist. Intensity of the staining was evaluated and a representative score (the score covering the largest tumor area) was assigned to each sample. Percentage of cancer cells positive for cytoplasmic HSP90 over total tumor area was quantified. Nuclear HSP90 was scored by quantifying percentage of cancer cells positive for nuclear HSP90 normalized to total tumor. Immunohistochemistry against ALK, ROS1, HER2, and BRAF^V600E^ was performed on 4‐µm‐thick sections of formalin‐fixed and paraffin‐embedded brain samples at Hospital 12 de Octubre. For immunostaining against ROS1 and BRAF^V600E^, a standardized automated protocol using the Leica Bond Polymer Refining Kit (Leica Bond‐III stainer, Leica Biosystems) was performed. Antigen retrieval was performed using 30´ EDTA, pH 9.0, followed by endogenous peroxides blocking with hydrogen peroxide. Slides were incubated with the corresponding primary antibodies (ROS1, 1:200; mAb3287, Cell Signaling; anti‐BRAF^V600E^ clone VE1, 1:50; E19294, Spring Bioscience). For immunostaining against ALK, a standardized automated protocol using the OptiView DAB IHC Detection Kit (BenchMark GT automated immunostainer, Ventana, Roche) was performed. Antigen retrieval was performed using CC1 92´, followed by endogenous peroxides blocking. Slides were incubated with the primary antibody (anti‐ALK clone D5F3, prediluted; 790–4794, Ventana). After the primary antibodies, slides were incubated with the corresponding secondary antibodies and visualization systems (OptiView DAB for ALK and Leica Bond Polymer Refining Kit for ROS1 and BRAF) conjugated with horseradish peroxidase. Immunohistochemical reaction was developed using DAB. Nuclei were counterstained with hematoxylin. Finally, the slides were dehydrated, cleared, and mounted with a permanent mounting medium for microscopic evaluation. For immunostaining against HER2, the Bond Oracle HER2 IHC System (Leica Biosystems) was used.

Samples used to evaluate the correlation of DDA1, UBE4B, or AHR with relapse belong to two patient cohorts analyzed. All cohorts were composed by women with a diagnosis of primary, non‐metastatic breast cancer with expression of estrogen and/or progestogen receptor > 1% and lack of HER2 amplification at Hospital 12 de Octubre. Cohort #1 aimed to analyze the impact of DDA1, UBE4B, or AHR protein levels in the relapse risk. Thus, it was constituted by all the hormone‐positive consecutive patients diagnosed in that hospital in 2001 (*N* = 251) in order to ensure long follow‐up. The second cohort (*N* = 11) of cases that showed distant relapse was collected to investigate potential up‐ or down‐regulation of DDA1, UBE4B, or AHR from the primary to the metastatic lesion; thus, pairs of primary tumor plus their metastases of hormone‐positive breast cancers, regardless of the status of traditional prognostic factors were gathered just on the basis of availability of biopsy from the metastatic setting. The study protocol was approved by the Institutional Review Board of Hospital 12 de Octubre (Ref: 11/137) and was conducted according to the principles expressed in the Declaration of Helsinki. Processing of these breast cancer tissues for routine histological analysis was fixed in 10% buffered formalin (Sigma‐Aldrich; St. Louis, MO, USA) and embedded in paraffin. Tissue microarrays were mounted with two 1‐mm cores per sample (Quick‐Ray Instruments, UNITMA). An expert pathologist examined a template H&E slide from each sample to select the areas for core selection. Immunohistochemical staining was performed on 2.5‐μm TMA sections. Immunohistochemistry was performed using an automated protocol developed for the Autostainer Link automated slide staining system (DAKO, Agilent) as described above. Corresponding TMA was acquired and digitalized using the AxioScan.Z1 system (Zeiss). Digitalized images were automatically analyzed with the ZEN 2.3 lite software (Zeiss). For staining quartile determination, H‐scores were calculated by formula: ((% of Area High Intensity × 3) + (% of Area Medium Intensity × 2) + (% of Area Low Intensity × 1))/100.

### Immunofluorescence and immunohistochemistry

Tissue for immunofluorescence was obtained after overnight fixation with 4% PFA at 4°C. Slicing of the brain was done by using a vibratome (Leica) or sliding microtome (Thermo Fisher Scientific). Both types of brain slices (250 μm and 80 μm, respectively) were blocked in 10% NGS, 2% BSA, and 0.25% Triton X‐100 in PBS for 2 h at room temperature (RT). Primary antibodies were incubated overnight at 4°C in the blocking solution and the following day for 30 min at RT. After extensive washing in PBS‐Triton 0.25%, the secondary antibody was added in the blocking solution and incubated for 2 h. After extensive washing in PBS‐Triton 0.25%, nuclei were stained with bisbenzamide (1 mg/ml; Sigma‐Aldrich) for 7 min at RT. Brain slices were pretreated with methanol for 20 min at −20°C before the blocking step for nuclear staining against DDA1 and p‐Histone H2A.X (Ser139). For staining against BrdU, mouse brain slices or PDOC were treated with HCI 2N 30 min at 37°C, followed by 0.1 M borate buffer (pH 8.5) incubation for 10 min at RT. After extensive washing in TBS, slices were blocked in 3% NGS in TBS‐Triton 0.25% for 1 h at RT and primary antibody was incubated for 72 h at 4°C. After extensive washing with TBS‐Triton 0.25%, the secondary antibody was incubated in blocking solution for 2 h at RT followed by extensive washing with TBS. Primary antibodies: GFP (1:1,000; GFP‐1020, Aves Labs), BrdU (1:500; ab6326, Abcam), Ki67 (1:500; ab15580, Abcam), Cleaved Caspase‐3 (Asp175) (1:500; #9661, Cell Signaling), HSP90α/β F‐8 (1:500; sc‐13119; Santa Cruz Biotechnology), HSP70/ HSC70 W27 (1:500; sc‐24; Santa Cruz Biotechnology), AHR (1:300; 31.714.200, US Biological), RPLP1 (1:100; HPA003368, Sigma‐Aldrich), DDA1 (1:100; 14995‐1‐AP; ProteinTech), NeuN (1:500; MAB377, Millipore), NeuN (1:500; ab177487, Abcam), collagen IV (1:1,000; AB756P, Millipore), GFAP (1:700; ab4674, Abcam), S100β EP1576Y (1:100; ab52642, Abcam), Iba1 (1:500; 019‐19741, Wako), Olig2 (1:500; AB9610, Millipore), and p‐Histone H2A.X (Ser139) (1:100; 05‐636, Millipore). Secondary antibodies: Alexa‐Fluor anti‐chicken 488, anti‐chicken 555, anti‐rabbit 488, anti‐rat 555, anti‐mouse 555, anti‐rabbit 555, anti‐rabbit 594, anti‐rabbit 633, anti‐mouse 647 (dilution 1:300; Invitrogen).

For squash preparations, mice with established brain metastases were sacrificed in a CO_2_ chamber and perfused with 4% PFA in 0.12 M phosphate buffer, pH 7.2‐7.4. Whole brains were dissected and postfixed in the same fixative for 2 h at RT. Coronal sections (400 µm) were obtained with a vibratome. Squash preparations of dissociated tumor cells were generated following a previously reported procedure (Pena *et al*, 2001). Briefly, small tissue fragments from the brain slices were dissected out under the control of a stereoscopic microscope. Each tissue fragment was transferred to a drop of PBS on a positively charged slide (SuperFrost^®^ Plus, Thermo Scientific). A coverslip was applied on top of the slide, and the tissue was squashed by percussion with a histologic needle to dissociate tumor cells. The preparation was frozen in dry ice and the coverslip removed. Most tumor cells remained adhered to the slide. Cell samples were processed in 96% ethanol at 4°C for 10 min, which increases the adhesion of cells to the slide, and rehydrated progressively in 70% ethanol and PBS. For immunofluorescence of squash preparations, cell samples were sequentially treated with 0.5% Triton X‐100 in PBS for 15 min, 0.1 M glycine in PBS containing 1% BSA for 30 min and 0.01% Tween‐20 in PBS for 5 min. Primary antibodies were incubated in 1% BSA overnight at 4°C and the following day for 30 min at RT (AHR, 1:300; 31.714.200, US Biological; UBE4B (UFD2), 1:100; sc‐377072, Santa Cruz Biotechnology). After extensive washing in 0.01% Tween‐20 in PBS, the corresponding secondary antibodies conjugated with Cy3 or Texas Red (Jackson Immunoresearch) were incubated for 1 h at RT followed by two washes in PBS. Samples were mounted with the antifading medium Vectashield (Vector Labs) containing DAPI for the cytochemical staining of DNA.

Immunohistochemistry staining against p62 and p‐ERK (Ventana Discovery XT, Roche) as well as Ki67 (Ventana Discovery ULTRA, Roche) was performed using a standardized automated protocol. Antigen retrieval was performed using cell conditioning solution (CC1 mild), followed by endogenous peroxides blocking with peroxide hydrogen at 3%. Slides were incubated with the corresponding primary antibodies (anti‐p62 Ick ligand clone 3/P62 LCK LIGAND, 1:50; 610832, BD Biosciences; phospho‐p44/42 MAPK (Erk1/2), 1:300; #9101, Cell Signaling; Ki67 (D3B5), 1:50; #12202, Cell Signaling). Slides were incubated with the corresponding secondary antibodies and visualization systems (OmniMap) conjugated with horseradish peroxidase. Immunohistochemical reaction was developed using Discovery Purple and ChromoMap DAB kits, and nuclei were counterstained with Carazzi’s hematoxylin. Finally, the slides were dehydrated, cleared, and mounted with a permanent mounting medium for microscopic evaluation.

### *MGMT* methylation‑specific PCR (MSP)

DNA from formalin‐fixed paraffin‐embedded tumor tissues was extracted using the QIAamp DNA FFPE Tissue Kit (Qiagen) following manufacturer’s instructions. A nested, two‐stage PCR approach to improve the sensitivity to detect methylated alleles was performed as previously described (Palmisano *et al*, 2000). Genomic DNA was subjected to bisulfite treatment using the Epitect Bisulfite Kit (Qiagen) and PCR was performed to amplify a 289 bp fragment of the MGMT promoter region. The primers recognize the bisulfite‐modified template but do not discriminate between methylated and unmethylated alleles. The stage 1 PCR products were diluted 50‐fold, and 5 μl was subjected to a stage 2 PCR in which primers specific to methylated or unmethylated template were used. Taq Gold polymerase (Thermo Fisher Scientific) in a 50 μl volume reaction was used in all PCRs. PCR amplification protocol for stage 1 was as follows: 95°C for 10 min, followed by denaturation at 95°C for 30 s, annealing at 52°C for 30 s and extension at 72°C for 30 s for 40 cycles followed by a final extension at 72°C for 10 min. PCR amplification protocol for stage 2 was as follows: 95°C for 15 min, followed by denaturation at 95°C for 30 s, annealing at 62°C for 30 s and extension at 72°C for 30 s for 2 cycles. Next, denaturation at 95°C for 30 s, annealing at 60°C for 30 s, and extension at 72°C for 30 s for 2 cycles was performed. Finally, denaturation at 95°C for 30 s, annealing at 58°C for 30 s, and extension at 72°C for 30 s for 36 cycles followed by a final extension at 72°C for 7 min was performed. Placental DNA treated with SssI methyltransferase (New England Biolabs) was used as a positive control for methylated alleles of MGMT, and DNA from normal lymphocytes was used as a negative control. Controls without DNA (blank) were used for each set of methylation‐specific PCR assays. 7 μl of each methylation‐specific PCR product was loaded directly into 3% agarose gel, stained with real safe (Durviz), and examined under ultraviolet illumination. Primers used to selectively amplify unmethylated or methylated MGMT gene in the stage 2 PCR were as previously described (Esteller *et al*, 2000; Hegi *et al*, 2005).

Primers (5′ > 3′, forward; reverse):

‐ *MGMT* (stage 1): (GGATATGTTGGGATAGTT; CCAAAAACCCCAAACCC)
‐ *MGMT* unmethylated (stage 2): (TTTGTGTTTTGATGTTTGTAGGTTTTTGT; AACTCCACACTCTTCCAAAAACAAAACA)
‐ *MGMT* methylated (stage 2): (TTTCGACGTTCGTAGGTTTTCGC; GCACTCTTCCGAAAACGAAACG)

### *EGFR* mutational analysis

DNA was extracted from FFPE tissue samples, and macrodissection was performed to ensure a content of at least 60% tumor cells. Samples were tested by real‐time PCR in a cobas z480 analyzer (Roche Diagnostics) using the cobas EGFR Mutation Test, which can detect mutations in exons 18, 19, 20, and 21 of the *EGFR* gene.

### qRT–PCR

Whole RNA was isolated using the RNAeasy Mini Kit (Qiagen) and was used (1,000 ng) to generate cDNA using iScript cDNA Synthesis Kit (1708891, Bio‐Rad) according to manufacturer’s instructions. RNA obtained from mouse brains included microdissected established metastases from human BrM cells. Gene expression in the tumor was analyzed by using human primers using SYBR green gene expression assays (GoTaq qPCR Master Mix, A6002, Promega).

Primers (5′ > 3′, forward; reverse):

‐ *HSP90AA1*: (AGATGACGACACATCACGCA; ACAGTGCACGTTACCCCAAT)
‐ *HSP90AB1*: (TGAGGAGGATGACAGCGGTA; TCAAAAAGGTCAAAGGGAGCC)
‐ *HSPA4* (HSP70): (GCAAGTGACTGCCATGCTTT; TAAGCAGAGTGGCCCATGTC)
‐ *HSPB2* (HSP27): (TAAACCTGGAAGCACCTCGG; ACATTGTGGACCATGCACCT)
‐ *HSF1*: (CCCTGATGCTGAACGACAGT; GGATAGGGGCCTCTCGTCTA)
‐ *AHR*: (ACAACCGATGGACTTGGGTC; TGGCAGGAAAAGGGTTGGTT)
‐ *UBE4B*: (GGTTGTGGTTGCCGAAATCC; CAGCCGCCATGTAACTGAGA)
‐ *DDA1*: (GCTACCTGCATCAGCAATGG; ACATCGGCTCCTATGAGGTTG)

### Transcriptomics of relapsed tumors

500 ng of total RNA samples were used. Sample RNA Integrity numbers were 8.6 on average (range 5.9–9.5) when assayed on an Agilent 2100 Bioanalyzer. Sequencing libraries were prepared with the QuantSeq 3‘mRNA‐Seq Library Prep Kit (FWD) for Illumina (Lexogen, Cat. No. 015) by following manufacturer’s instructions. Library generation is initiated by reverse transcription with oligodT priming, and a second strand synthesis is performed from random primers by a DNA polymerase. Primers from both steps contain Illumina‐compatible sequences. Libraries are completed by PCR {This kit generates directional libraries stranded in the sense orientation: the read1, the only read in single read format, has the sense orientation (‐‐library‐type fr‐secondstrand in TopHat, ‐‐stranded = yes in HTSeq)}. cDNA libraries are purified, applied to an Illumina flow cell for cluster generation and sequenced on an Illumina NextSeq 550 (with v2.5 reagent kits) by following manufacturer's protocols. Eighty‐five‐base‐pair single‐end sequenced reads followed adapter and polyA tail removal as indicated by Lexogen. The resulting reads were fed to Xenome (Conway *et al*, 2012) to separate the xenograft‐derived human and mouse reads. Human reads were analyzed with the nextpresso (Graña *et al*, 2018) pipeline as follows: sequencing quality was checked with (https://www.bioinformatics.babraham.ac.uk/projects/fastqc/) FastQC v0.11.0. Reads were aligned to the human genome (GRCh38) with TopHat‐2.0.10 (Trapnell *et al*, 2009) using Bowtie 1.0.0 (Langmead *et al*, 2009) and Samtools 0.1.19 (Li *et al*, 2009), allowing 3 mismatches and 20 multihits. The Gencode v29 gene annotation for GRCh38 was used. Read counts were obtained with HTSeq (Anders *et al*, 2015). Differential expression and normalization were performed with DESeq2 (Love *et al*, 2014), filtering out those genes where the normalized count value was lower than 2 in more than 50% of the samples. From the remaining genes, those that had an adjusted p value below 0.05 FDR were selected. GSEAPreranked (Subramanian *et al*, 2005) was used to perform gene set enrichment analysis for several gene signatures on a pre‐ranked gene list, setting 1,000 gene set permutations. Only those gene sets with significant enrichment levels (FDR *q*‐value < 0.25) were considered. Access to RNAseq data is provided from the Gene Expression Omnibus, under the ID GSE153173.

### *In situ* proteomics

Fixed organotypic cultures were embedded in paraffin. 10‐µm sections were placed on PET‐membrane slides (415190‐9051‐000, Zeiss) pretreated with UV light. Slides were stained for 5 min in hematoxylin solution and 30 s in eosin solution, and were left unmounted. Fully established brain metastases were isolated using the ArcturusXT™ Laser Capture Microdissection System (Thermo Scientific) and Arcturus^®^ CapSure^®^ Macro LCM Caps (Life Technologies) according to the manufacturer's protocol. Each dissection was validated by inspection of the cap and the sample. At least 12 brain metastases per biological sample were dissected. Dissected samples were processed using the commercially available in‐StageTip‐NHS kit (PreOmics GmbH) according to the manufacturer's protocol. Peptides were dissolved in HPLC‐grade water containing 0.1% formic acid and 2% acetonitrile. Randomization for sample run order was applied and the samples were individually analyzed using shot‐gun liquid chromatography tandem mass spectrometry (LC‐MS/MS) on a high accuracy Orbitrap Fusion™ Lumos™ Tribrid™ Mass Spectrometer (Thermo Fisher) coupled to an Acquity M nanoflow system (Waters GmbH). Samples were analyzed using 120 min gradient, top12 loop count, mass range 350–1,500 m/z, and an Acquity UPLC^®^ M class 250 mm × 75 µM column. All raw files from LC‐MS/MS were processed with MaxQuant (version 1.6.2.6) using the standard settings against a human protein database (UniProtKB/Swiss‐Prot, 20,373 sequences) supplemented with contaminants. Label‐free quantification was done with match between runs (match window of 0.7 min and alignment window of 20 min). Carbamidomethylation of cysteines was set as a fixed modification, whereas oxidation of methionines and protein N‐term acetylation as variable modifications. Minimal peptide length was set to 7 amino acids and a maximum of two tryptic missed‐cleavages were allowed. Data are available via ProteomeXchange with identifier PXD020092. Results were filtered at 0.01 FDR (peptide and protein level). Then, the “proteinGroups.txt” file was loaded in Prostar (v1.14) (Wieczorek *et al*, 2017) for further statistical analysis. Briefly, global normalization across samples was performed using the LOESS function and missing values were imputed using the algorithms slsa (for partially observed values) and detquantile (for values missing on an entire condition). Differential analysis was done using the empirical bayes statistics limma. Proteins with a *P* < 0.05 and a log_2_ ratio > 1 or < −1 were defined as deregulated. The FDR was estimated to be 14% by Benjamini‐Hochberg. Functional analysis was performed with the GSEApreranked function (biocarta, canonical pathways, GO, KEGG, OncogenicSignatures, Reactome, TFs) using the log_2_ ratios as the input file to identify top 25 upregulated and downregulated signatures defined by NES values, FDR < 25% and *P* < 0.01.

### Analysis of patient progression

After tumor resection, glioblastoma patients were treated by the standard of care, radiotherapy (Rx) plus temozolomide (TMZ), according to the Stupp protocol (Stupp *et al*, 2005, 2009). MRI with volumetric analysis of brain lesions was performed at the following time‐points: immediately post‐surgery (24–48 h after resection), 1–2 weeks before chemoradiotherapy (Rx+TMZ), 4 weeks after chemoradiotherapy and then every 2–3 months depending on the clinical evolution. Patients were followed‐up until decease or the end of the study. Tumor response to treatment was assessed according to RANO criteria (Wen *et al*, 2010).

### Analysis of prognosis

The previously published exome capture RNA‐sequencing dataset of 21 cases of breast cancer brain metastases with clinical annotation (Varešlija *et al*, 2019) has since been expanded to 45 cases (*N* = 90 patient‐matched samples; GSE184869). Gene expression of individual HSP90 target genes was assessed using log_2_ transformed trimmed M of means (TMM)‐normalized counts per million (log_2_(TMM‐CPM + 1)). Two groups of patients with low or high gene expression were delineated using the maximally selected rank statistics (Hothorn & Lausen, 2003), as implemented in the “survminer” R package (Kassambara *et al*, 2019) and Kaplan‐Meier survival curves were generated depicting survival post‐brain metastasis. P values were obtained with long‐rank (Mantel‐Cox) two‐sided tests. In order to obtain the HSP90 signature score, first the z‐score of each individual target gene was calculated for all patients ((x – μ)/σ). For each patient, z‐scores of the respective target genes were added up and the average was calculated, resulting in the individual HSP90 signature score. Division into two groups (high/low) according to the HSP90 signature score and generation of Kaplan‐Meier survival curves was performed using the above‐described approach. All samples were in compliance with protocols approved by the corresponding IRB (University of Pittsburgh IRB#PRO15050502, Royal College of Surgeons in Ireland IRB#13/09/ICORG09/07 and the Mayo Clinic Cancer Center Institutional Review Board).

### Pharmacokinetics assay

Plasma and brain samples were collected 6 h after oral administration of DEBIO‐0932 (160 mg/kg) to brain metastases‐bearing mice. Around 1 ml of blood was centrifuged at 1000 *g* for 10 min at 4°C immediately after the extraction. Brain samples were homogenized in 4 volumes of H_2_O and sonicated for 10 min followed by centrifugation at 11,200 *g* for 5 min. The supernatant was stored at −20°C until processing. The extraction of DEBIO‐0932 was achieved by solid‐phase extraction followed by high‐performance liquid chromatography/tandem mass spectrometry (Agilent 1100, Sciex QTRAP 5500 System) analysis. The amount of DEBIO‐0932 in each sample was quantified based on calibration curves generated using standards of known concentrations of DEBIO‐0932. For the conversion of brain concentrations in ng/g to ng/mL, a tissue density of 1 was assumed.

### Magnetic resonance imaging in mice

Magnetic resonance imaging studies were carried out in a Bruker Biospec 70/20 scanner using a combination of a linear coil (for transmission) with a mouse head phase array coil (for reception). Animals were anesthetized with sevoflurane (5% for induction and 3% for maintenance) and placed in an MRI‐adapted stereotaxic holder with a water circulating blanket to maintain body temperature. Respiration and body temperature were continuously monitored. As anatomical reference, a T2‐weighted sequence was acquired (TR = 4600 ms; TE, 65 ms; α = 90°; FOV = 1.5 × 1.5 cm; matrix = 192 × 192; slice thickness = 0.5 mm, number of slices = 30). Then, a T1 sequence was acquired (TR = 472.610 ms; TE, 3.648 ms; α = 30°; FOV = 1.5 × 1.5 cm; matrix = 192 × 192; slice thickness = 0.5 mm, number of slices = 30) before and after intravenous administration of 200 µl of Gadovist (1 mmol/ml, Bayer AG).

### Image acquisition and analysis

Immunofluorescence images were acquired with a Leica SP5 up‐right confocal microscope ×5, ×10, ×20, ×40, and ×63 objectives and analyzed with ImageJ software and Definiens developer XD 2.5. Immunohistochemistry images were captured with the Zen Blue Software v3.1 (Zeiss), and whole slides were acquired with a slide scanner (AxioScan Z1, Zeiss). For histological quantification of brain metastases at endpoint (5 weeks after intracardiac inoculation of cancer cells), only lesions showing solid and compact distribution of cancer cells were considered as established metastases.

### Statistical analysis

Data are represented as the mean ± s.e.m. Comparisons between two experimental groups were analyzed with unpaired, two‐tailed Student’s *t*‐test. Survival analysis was done with log‐rank (Mantel‐Cox) test. Analysis of relapse cohort include quantification of the staining levels of DDA1, UBE4B or AHR with an H‐score, and categorized in binary variables according to the H‐score (above or below the average level for each cohort). The individual role of each gene in the relapse risk was calculated with the Kaplan‐Meier method and the Log‐Rank test; the attributable time‐dependent relative risk for each gene was calculated with the Cox’s Proportional Hazards Model, adjusted by the traditional factors (T, N, Grade, age and Ki67). The cross‐correlation between the levels of (3 genes) was tested with R2 Pearson’s test, and dot‐plot charts were generated.

Comparison of the levels of DDA1, UBE4B or AHR among the different cohorts (relapsed vs non‐relapsed, primary vs metastasis) was performed with a parametric *T*‐test comparison of the average H‐stainings, assuming homogeneous variances across subgroups.

All statistical tests were two‐sided and the statistical significance level was set at 0.05. Calculations were performed with the SPSS Statistics V. 19.0 software.

## Author contributions

**Lucía Zhu:** Conceptualization; Data curation; Formal analysis; Validation; Investigation; Visualization; Methodology; Writing—original draft; Writing—review and editing. **Diana Retana:** Formal analysis; Validation; Investigation; Visualization; Methodology. **Pedro García‐Gómez:** Formal analysis; Validation; Investigation; Visualization; Methodology; Writing—review and editing. **Laura Álvaro‐Espinosa:** Formal analysis; Investigation; Methodology. **Neibla Priego:** Formal analysis; Investigation; Methodology. **Mariam Masmudi‐Martín:** Formal analysis; Investigation; Methodology. **Natalia Yebra:** Formal analysis; Visualization; Methodology. **Lauritz Miarka:** Data curation; Formal analysis; Validation; Writing—review and editing. **Elena Hernández‐Encinas:** Formal analysis; Methodology. **Carmen Blanco‐Aparicio:** Resources; Formal analysis; Validation; Investigation; Methodology. **Sonia Martínez:** Formal analysis; Validation; Investigation; Visualization; Methodology. **Cecilia Sobrino:** Investigation; Methodology. **Nuria Ajenjo:** Resources; Project administration. **Maria‐Jesus Artiga:** Supervision; Project administration. **Eva Ortega‐Paino:** Resources; Supervision; Project administration. **Raúl Torres‐Ruiz:** Investigation; Methodology. **Sandra Rodríguez‐Perales:** Methodology. **Adolfo de la Lama‐Zaragoza:** Human samples. **Lourdes Calero‐Felix:** Human samples. **Concepcion Fiaño‐Valverde:** Human samples. **Pedro, David Delgado‐López:** Human samples. **Antonio Montalvo‐Afonso:** Human samples. **Mar Pascual‐Llorente:** Human samples. **Ángela Díaz‐Piqueras:** Human samples. **SH Nam‐Cha:** Human samples. **Cristina Barrena López:** Human samples. **Gerard Plans Ahicart:** Human samples. **Elena Martinez‐Saez:** Project administration; Human samples. **Santiago Ramón y Cajal:** Supervision; Project administration; Human samples. **Pilar Nicolás:** Supervision; Project administration; Human samples. **Riccardo Soffietti:** Resources; Writing—review and editing. **Luca Bertero:** Formal analysis; Validation; Methodology. **Paola Cassoni:** Resources; Supervision. **Tobias Weiss:** Resources; Formal analysis; Validation; Investigation; Methodology; Writing—review and editing. **Javier Muñoz:** Resources; Formal analysis; Validation; Visualization; Methodology; Writing—review and editing. **Juan Manuel Sepúlveda:** Resources; Data curation; Formal analysis; Validation; Investigation; Visualization; Methodology; Writing—review and editing. **Pedro González‐León:** Resources; Investigation; Methodology. **Luis Jiménez‐Roldán:** Resources; Formal analysis; Investigation; Methodology. **Luis Miguel Moreno:** Resources; Validation; Methodology. **Olga Esteban:** Resources; Validation; Investigation; Methodology. **Ángel Pérez‐Núñez:** Resources; Formal analysis; Supervision; Investigation; Visualization; Writing—review and editing. **Aurelio Hernández‐Laín:** Data curation; Supervision; Validation; Investigation; Methodology; Writing—review and editing. **Oscar Toldos:** Validation; Methodology. **Yolanda Ruano:** Methodology. **Lucía Alcázar:** Human samples. **Guillermo Blasco:** Human samples. **José Fernández‐Alén:** Supervision; Project administration. **Eduardo Caleiras:** Data curation; Formal analysis; Validation; Investigation; Visualization; Methodology. **Miguel Lafarga:** Formal analysis; Supervision; Validation; Investigation; Visualization; Methodology; Writing—review and editing. **Diego Megías:** Validation; Investigation; Visualization; Methodology. **Osvaldo Graña‐Castro:** Data curation; Formal analysis; Validation; Methodology. **Carolina Nör:** Resources; Supervision; Validation; Investigation; Methodology. **Michael D Taylor:** Resources; Supervision. **Leonie S Young:** Resources; Data curation; Supervision; Methodology. **Damir Varešlija:** Data curation; Validation; Investigation; Visualization; Methodology; Writing—review and editing. **Nicola Cosgrove:** Resources; Data curation; Methodology; Writing—review and editing. **Fergus J Couch:** Resources; Data curation. **Lorena Cussó:** Formal analysis; Methodology. **Manuel Desco:** Formal analysis; Supervision; Visualization; Methodology. **Michael Weller:** Supervision; Validation; Methodology. **Joaquín Pastor:** Resources; Formal analysis; Supervision; Validation; Investigation; Visualization; Methodology. **Manuel Valiente:** Conceptualization; Resources; Data curation; Formal analysis; Supervision; Funding acquisition; Validation; Investigation; Visualization; Methodology; Writing—original draft; Project administration; Writing—review and editing. **Silvana Mouron:** Data curation; Formal analysis; Visualization; Methodology; Writing—review and editing. **Miguel Quintela‐Fandino:** Data curation; Formal analysis; Supervision; Visualization; Methodology; Writing—review and editing.

In addition to the CRediT author contributions listed above, the contributions in detail are:

LZ and MV designed and performed the experiments, analyzed the data, and wrote the manuscript. DR, PG‐G, LA‐E, NP, MM‐M, and NY performed the experiments and analyzed the data. EH‐E and LZ performed the pharmacokinetic experiments and analyzed the data. TW, JM, MW, and LZ performed the proteomic experiments and analyzed the data. PG‐G and ML performed and analyzed the squash preparations. DM quantified the immunofluorescence images. OG‐C performed the transcriptomic analysis. CN and MDT provided reagents and technical expertise with the neurosurgery experiments. RT‐R, SR‐P, and LZ performed and analyzed the genetic experiments. LC and MD performed the MRI experiments and analyzed the data. LB, PC, RS, JMS, PG‐L, LJ‐R, LMM, OE, AP‐N, AH‐L, YR, OT, LA, GB, JF‐A, NA, M‐JA, CS, EO‐P, and members of RENACER provided and processed the human samples and analyzed the clinical data. EC scored the clinical samples. LSY, DV, NC, and FJC provided RNAseq data of the matched breast cancer brain metastasis patient cohort. LM analyzed the correlation between gene expression and patient survival. JP selected and proposed DEBIO‐0932 and chlorpromazine as candidates for described experiments. CB‐A and SM performed the *in vitro* screening and provided the chemical inhibitors. MQ‐F, SiM, and LZ evaluated the relapse cohort.

## For more information

https://valientelab.com/.

### RENACER members and their affiliations

Adolfo de la Lama‐Zaragoza, Servicio de Neurocirugía, Hospital Alvaro Cunqueiro, Complejo Hospitalario de Vigo, Vigo, 36213, Spain.

Lourdes Calero‐Felix, Servicio de Neurocirugía, Hospital Alvaro Cunqueiro, Complejo Hospitalario de Vigo, Vigo, 36213, Spain.

Concepcion FiañoValverde, Servicio de Anatomía Patológica, Hospital Alvaro Cunqueiro, Complejo Hospitalario de Vigo, Vigo, 36213, Spain.

Pedro David Delgado‐López, Servicio de Neurocirugía, Hospital Universitario de Burgos, Burgos, 09006, Spain.

Antonio Montalvo‐Afonso, Servicio de Neurocirugía, Hospital Universitario de Burgos, Burgos, 09006, Spain.

Mar Pascual‐Llorente, Servicio de Anatomía Patológica, Hospital Universitario de Burgos, Burgos, 09006, Spain.

Ángela Díaz‐Piqueras, Biobanco, Unidad de Investigación y Complejo Hospitalario Universitario de Albacete, Albacete, 02006, Spain.

SH Nam‐Cha, Anatomía Patológica, Unidad de Investigación y Complejo Hospitalario Universitario de Albacete, Albacete, 02006, Spain.

Cristina Barrena López, Servicio de Neurocirugía, Unidad de Investigación y Complejo Hospitalario Universitario de Albacete, Albacete, 02006, Spain.

Gerard Plans Ahicart, Servicio de Neurocirugía, Hospital Universitario de Bellvitge, L´Hospitalet de Llobregat, 08907, Spain.

Elena Martínez‐Saez, (1) Pathology Department, Vall d’Hebron Hospital, Barcelona, 08035, Spain; (2) Spanish Biomedical Research Network Centre in Oncology (CIBERONC).

Santiago Ramón y Cajal, (1) Pathology Department, Vall d’Hebron Hospital, Barcelona, 08035, Spain; (2) Spanish Biomedical Research Network Centre in Oncology (CIBERONC).

Pilar Nicolás, Faculty of Law, University of the Basque Country, Leioa, 48940, Spain.

## Supporting information



Appendix

Expanded View Figures PDF

Table EV1

Table EV2

Table EV3

Table EV4

Table EV5

Table EV6

Table EV7

Table EV8

Table EV9

Table EV10

Dataset EV1

## Data Availability

The datasets produced in this study are available in the following databases: RNAseq data: Gene Expression Omnibus GSE153173 (https://www.ncbi.nlm.nih.gov/geo/query/acc.cgi?acc=GSE153173). RNAseq data: Gene Expression Omnibus GSE184869 (https://www.ncbi.nlm.nih.gov/geo/query/acc.cgi?acc=GSE184869). Proteomics data: ProteomeXchange PXD020092 http://proteomecentral.proteomexchange.org/cgi/GetDataset?ID=PXD020092).
