# Supplementary material for: A clinically compatible drug‐screening platform based on organotypic cultures identifies vulnerabilities to prevent and treat brain metastasis
Source: EMBO Mol Med. 2022 Feb 17;14(3):e14552. doi: 10.15252/emmm.202114552 (PMC8899920; doi:10.15252/emmm.202114552)
Supplement: Supplementary file 1 — Appendix [file EMMM-14-e14552-s005.pdf]

A clinically-compatible drug-screening platform based on organotypic cultures identifies vulnerabilities to prevent and treat brain metastasis.

Appendix

Table of contents

Appendix Figure S1
Appendix Figure S2
Appendix Figure S3

Appendix Figure S1 - Evaluation of DMSO concentration in organotypic cultures.

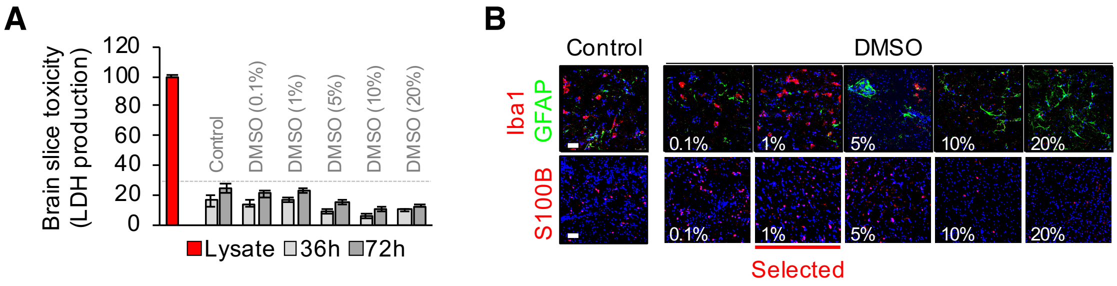

A Quantification of LDH levels in the conditioned media of wild type organotypic slices cultured during 1.5days and 3 days in the presence of different concentrations of DMSO. Values are shown as mean ± s.e.m. (n=3 organotypic cultures per experimental condition, 1 independent experiment) relative to a lysate of the same preparation.

B Representative images of the immunophenotyping of the resident brain microenvironment using Iba1 (microglia), GFAP (reactive astrocytes) and S100B (pan-astrocyte marker) upon 3 days culture with various concentrations of DMSO. The DMSO concentration selected it is underlined in red. Scale bar: 50 µm.

Appendix Figure S2 - Monogenic loss of function of *UBE4B* and *DDA1*.

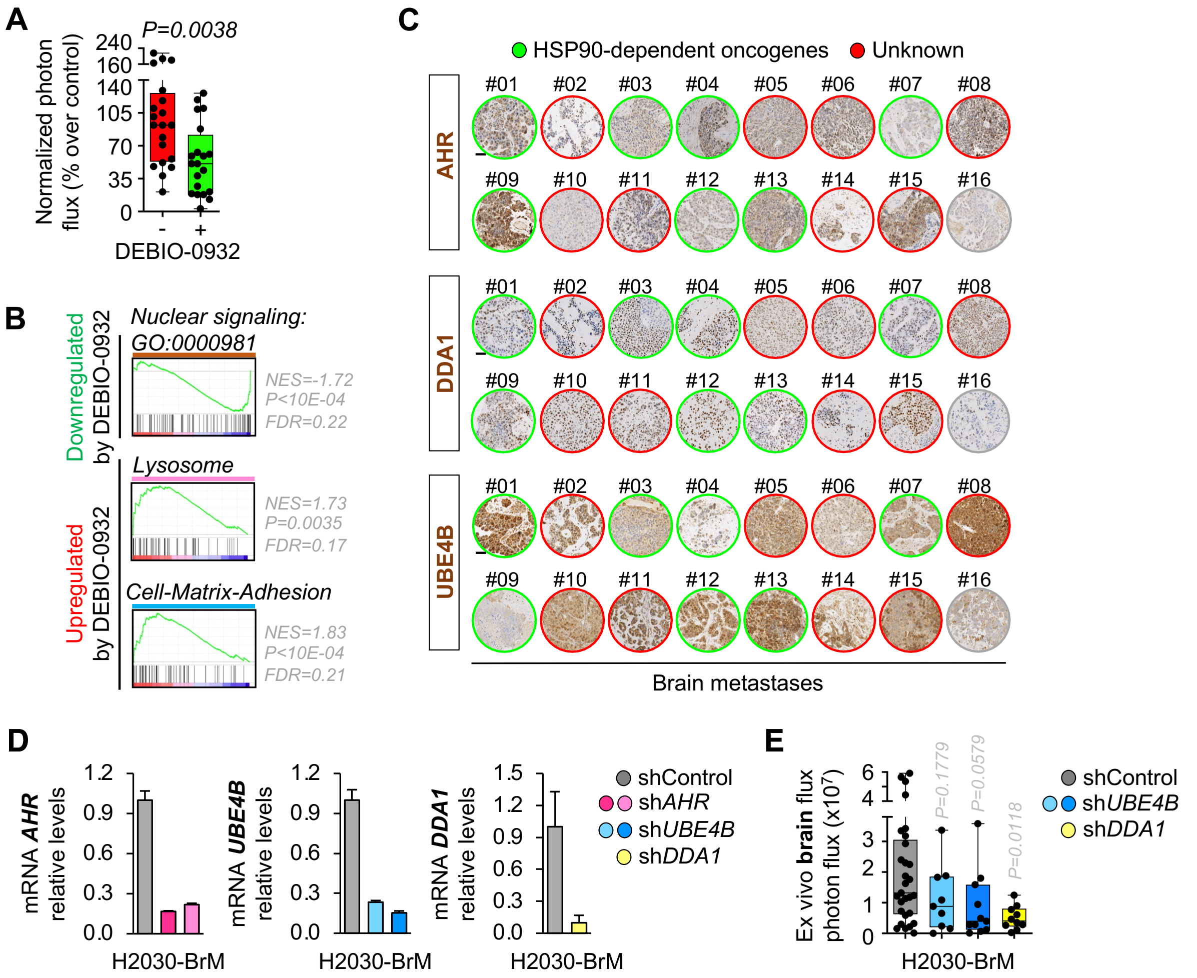

A Quantification of bioluminescence emitted by H2030-BrM established metastases in brain organotypic cultures. BLI values were obtained 6h after the addition of DEBIO-0932 or DMSO normalized by the values of each culture before any treatment. Data is shown as relative to DMSO BLI values in box-and-whisker plots where every dot represents an organotypic culture and the line in the box corresponds to the median. The boxes go from the upper to the lower quartiles and the whiskers go from the minimum to the maximum value (n=20 organotypic cultures per experimental condition, 3 independent experiments). *P* value was calculated using two-tailed t-test.
B Examples of signatures included in the main biological processes represented in the proteomic analysis. GO:0000981 corresponds to the Gene Ontology signature “DNA binding, Transcription factor activity, RNA polymerase II specific”.
C Immunohistochemistry against AHR, DDA1 and UBE4B in 16 human brain metastases with (green) and without (red) HSP90-dependent oncogenes. No molecular information available for the sample depicted in gray. Some representative images are reused in Fig 6H Scale bar: 50 µm.
D qRT-PCR of H2030-BrM cell lines with the indicated shRNA showing mean values ± s.e.m of three technical replicas for each cell line. Knockdowns were checked after generating the cell line and before injecting them into mice.
E Quantification of *ex vivo* BLI of brains at the endpoint of the experiment after intracardiac injection of H2030-BrM cells with indicated knockdowns. Values are

shown in box-and-whisker plots where every dot represents a different animal and the line in the box corresponds to the median. Whiskers go from the minimum to the maximum value (n=29 shControl mice, n=9 sh*UBE4B#1* (light blue), n=11 sh*UBE4B#2* (dark blue), and n=10 sh*DDA1*). *P* value was calculated using two-tailed t-test.

Appendix Figure S3 - METPlatform facilitates unbiased identification of synergistic drug combinations against brain metastasis.

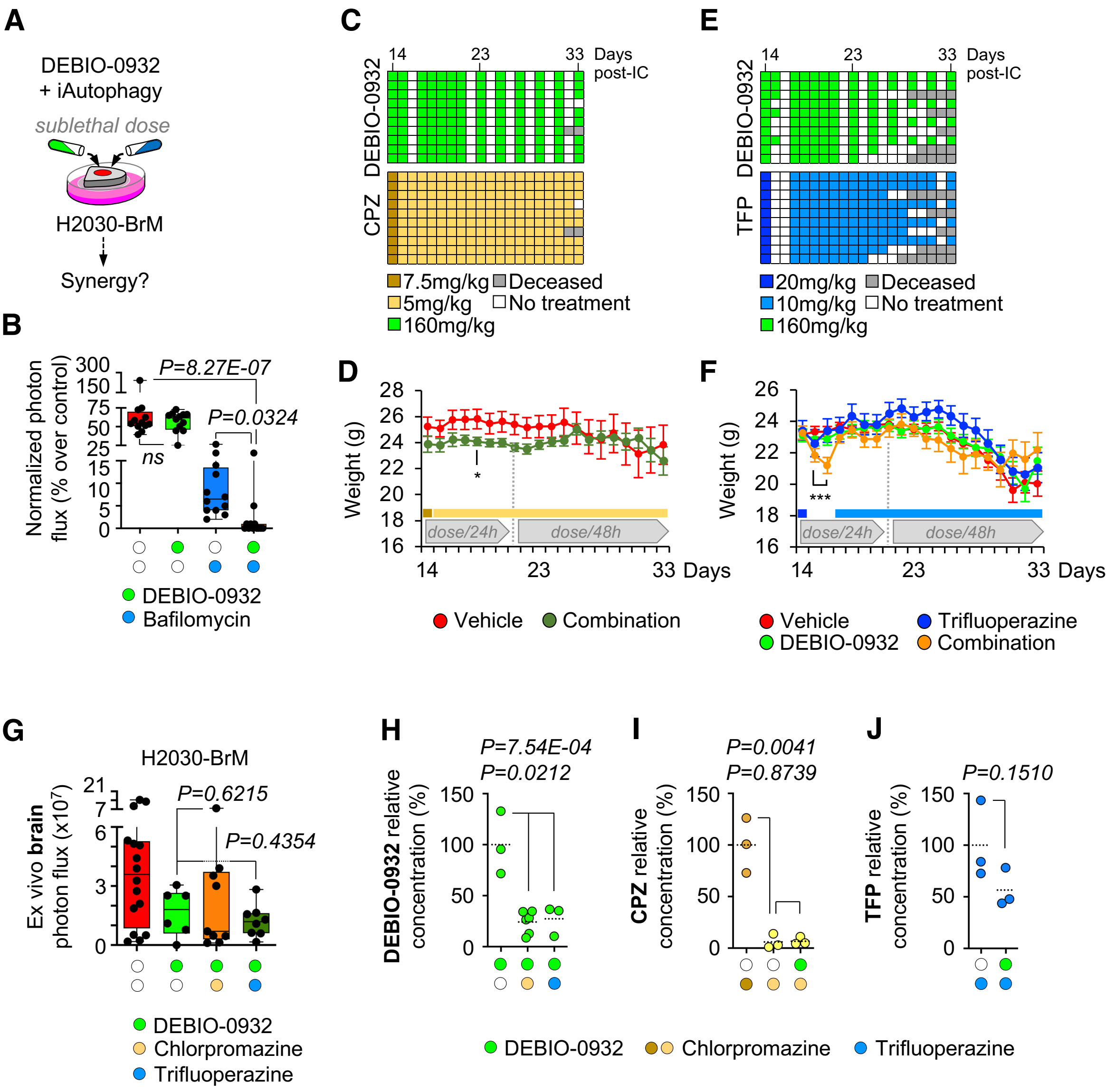

A Schema of experimental design. Organotypic cultures with established brain metastases from H2030-BrM cells were treated with DEBIO-0932 and autophagy inhibitors at sublethal doses.
B Quantification of the bioluminescence signal emitted by H2030-BrM cells in each organotypic culture with established brain metastases at day 7 normalized by the initial value at day 0 (before the addition any treatment; both DEBIO-0932 and bafilomycin were added at 100 nM) and normalized to the organotypic cultures treated with DMSO. Values are shown in box-and-whisker plots where every dot represents an organotypic culture and the line in the box corresponds to the median. Whiskers go from the minimum to the maximum value (n=12 organotypic cultures per experimental condition, 2 independent experiments). *P* value was calculated using two-tailed t-test.
C Schema showing the individualized therapy of mice receiving DEBIO-0932 and chlorpromazine (CPZ) during the treatment period starting 14 days after intracardiac injection of H2030-BrM cell line. Each row represents a mouse receiving DEBIO-0932

(green) and CPZ (yellow) or not (white) (n=10 mice treated with the combination therapy, n=10 mice treated with vehicle, 1 independent experiments). Dark yellow indicates initially selected dose of CPZ, which had to be reduced to 5mg/kg. Gray squares indicate decease of the corresponding animal. Although DEBIO-0932 was expected to be given daily during the first week, high dose of CPZ required adapting this schedule.

D Animal weight from vehicle and DEBIO-0932/ CPZ treated mice from (C). Values are shown as mean ± s.e.m. (n=10 vehicle and n=10 DEBIO-0932/ CPZ treated mice). *P* value was calculated using two-tailed t-test (*P* values: **P*<0.05)

E Schema showing the individualized therapy of mice receiving DEBIO-0932 and trifluoperazine (TFP) during the treatment period starting 14 days after intracardiac injection of H2030-BrM cell line. Each row represents a mouse receiving DEBIO-0932 (green) and TFP (yellow) or not (white) (n=10 mice treated with the combination therapy, n=10 mice treated with vehicle, 1 independent experiments). Dark blue indicates initially selected dose of TFP, which had to be reduced to 10mg/kg after mice recovery. Gray squares indicate decease of the corresponding animal. Although DEBIO-0932 was expected to be given daily during the first week, high dose of TFP required adapting this schedule.

F Animal weight from vehicle, monotherapy and combination therapy with DEBIO-0932/ TFP treated mice from (E). Values are shown as mean ± s.e.m. (n=16 vehicle, n=16 DEBIO-0932, n=10 TFP, n=10 DEBIO-0932/ TFP treated mice). *P* value was calculated using two-tailed t-test (*P* values: ****P*<0.001)

G Quantification of *ex vivo* BLI at the endpoint of the experiment (5 weeks after injection of cancer cells) of brains of mice inoculated with H2030-BrM and treated with the indicated drugs. Values are shown in box-and-whisker plots where every dot represents a different animal and the line in the box corresponds to the median. Whiskers go from the minimum to the maximum value (n=16 vehicle, n=6 DEBIO-0932, n=9 DEBIO-0932/ CPZ, n=8 DEBIO-0932/ TFP treated mice). *P* value was calculated using two-tailed t-test.

H-J LC-MS/ MS quantification of DEBIO-0932 (H), CPZ (I) and TFP (J) from brains at the endpoint of the experiment described in (C, E). Values are normalized to the respective control. Brains were processed 6 hours after the last dose of the respective inhibitor. (n=3 brains per treatment were used with the exception of the analysis of DEBIO-0932 in combination with CPZ where 5 brains were used). Dark yellow correspond to high dose CPZ (7.5mg/kg). *P* value was calculated using two-tailed t-test.
