# Supplementary material for: A clinically compatible drug‐screening platform based on organotypic cultures identifies vulnerabilities to prevent and treat brain metastasis
Source: EMBO Mol Med. 2022 Feb 17;14(3):e14552. doi: 10.15252/emmm.202114552 (PMC8899920; doi:10.15252/emmm.202114552)
Supplement: Supplementary file 2 — Expanded View Figures PDF [file EMMM-14-e14552-s001.pdf]

Expanded View Figures

Figure EV1. A chemical library applied to METPlatform identifies potential vulnerabilities of brain metastasis.

A Quantification of the proliferation of H2030-BrM cells at day 3 normalized to the cells treated with DMSO measured with CellTiter-Glo®. Green: hits, compounds with ≤ 20% proliferation; gray: compounds with > 20% proliferation. Values are shown in box-and-whisker plots where the line in the box corresponds to the mean. Each experimental compound of the library was assayed by duplicate. Hits highlighted in bold were common to the *ex vivo* screening (Fig 1B).

B Quantification of the bioluminescence signal from MDA231-BrM established brain metastases in organotypic culture after 3 days in culture. Values were normalized by the level of bioluminescence at Day 0 for each culture (before the addition of DMSO or any compound). Final data is shown in percentage respect to reference, the organotypic cultures treated with DMSO. Blue: DMSO-treated organotypic cultures; red: hits, compounds with normalized BLI ≤ 20% (dashed line); green: BKM120; gray: compounds with normalized BLI > 20%. Values are shown in box-and-whisker plots where the line in the box corresponds to the mean. Boxes extend from the minimum to the maximum value (n = 14 DMSO; n = 13 BKM120-treated organotypic cultures; each experimental compound was assayed by duplicate, 4 independent experiments).

C Detailed representation of the data shown in Figs 1B, EV1A and Table EV1 indicating relative viability using bioluminescence generated by H2030-BrM cells *ex vivo* (established brain metastases, light red), *in vitro* 2D (green) and *in vitro* 3D (spheroids, yellow) treated with compounds of the anti-tumoral library (compounds were assayed by duplicate in each assay). All hits for any condition are shown. The rectangles of the top indicate whether a given compound was effective (< 20% luminescence respect to control) *ex vivo* (light red rectangle), *in vitro* 2D (green rectangle), *in vitro* 3D (yellow rectangle).

D Representative wild-type brain slices treated with DMSO or the HSP90 inhibitor geldanamycin stained with anti-Col.IV (endothelial cells) and anti-NeuN (neurons). Scale bar: 50 µm.

E Representative wild-type liver slices treated with DMSO or the HSP90 inhibitor geldanamycin and stained with anti-Ki67 to score proliferation. BB: bisbenzamide. Scale bar: 50 µm.

F Quantification of GI_{50} values of geldanamycin in a panel of BrM cell lines *in vitro* from various primary origins and oncogenomic profiles. Nine serial concentrations of geldanamycin were assayed by duplicate and GI_{50} was calculated from a viability curve normalized to DMSO-treated cells of the corresponding cell line. Values are shown as mean + s.e.m. (each concentration was assayed by technical duplicates for each cell line and the experiment was performed twice).

G Quantification of the bioluminescence signal emitted by MDA231-BrM established metastases in organotypic cultures incubated in the presence of DEBIO-0932 (1 µM) during 3 days. Bioluminescence at Day 3 is normalized by the initial value obtained at day D and quantified relative to the organotypic cultures treated with DMSO. Day 0 is considered right before addition of the treatment or DMSO. Values are shown in box-and-whisker plots where each dot is an organotypic culture and the line in the box corresponds to the median. The boxes go from the upper to the lower quartiles and the whiskers go from the minimum to the maximum value (n = 6 organotypic cultures per experimental condition, 1 experiment). P value was calculated using two-tailed t-test.

 © 2022 The Authors

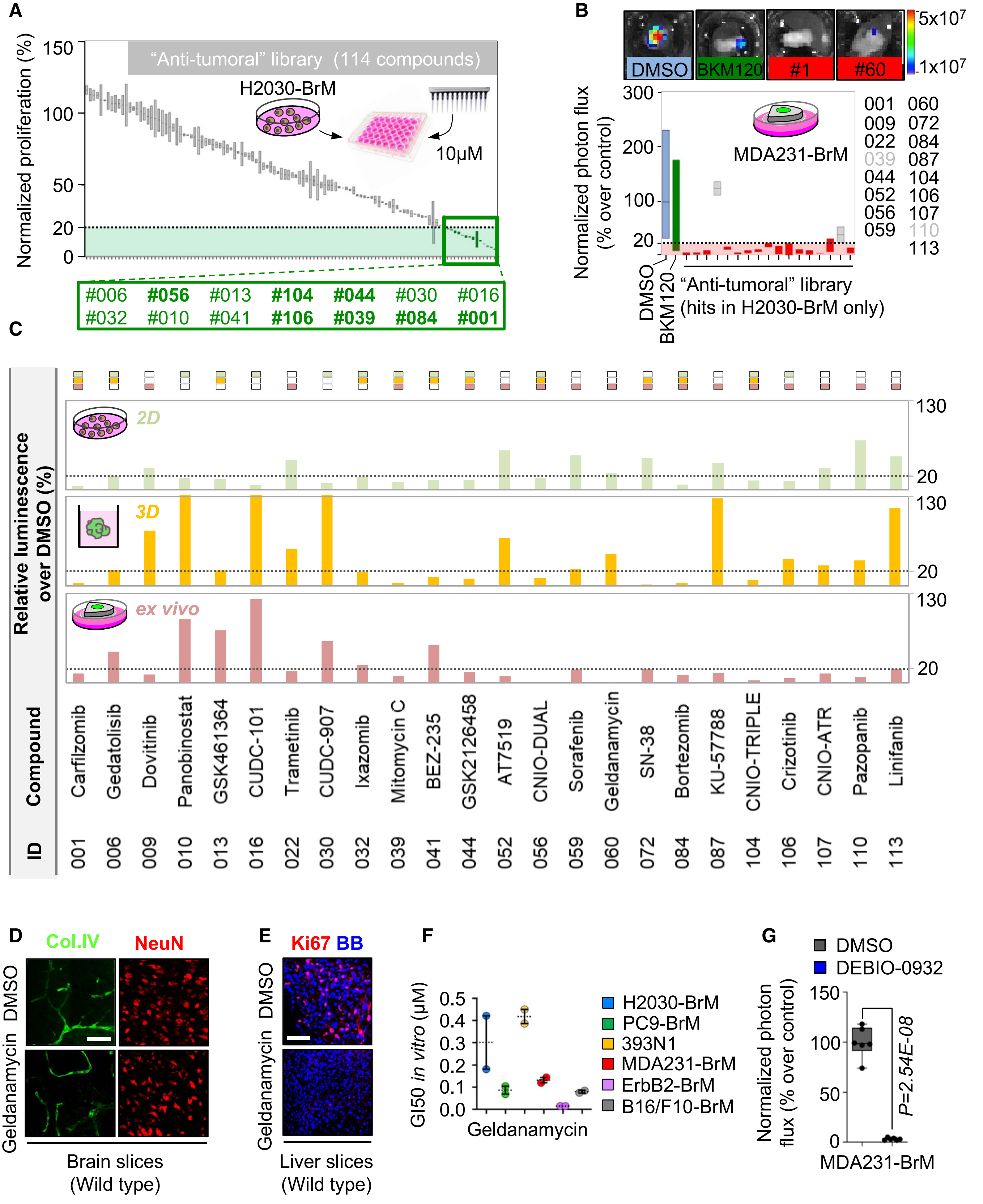
A
Normalized proliferation (%)
"Anti-tumoral" library (114 compounds)
H2030-BrM
10µM
#006 #056 #013 #104 #044 #030 #016
#032 #010 #041 #106 #039 #084 #001
B
DMSO BKM120 #1 #60
5x10^7
1x10^7
Normalized photon flux (% over control)
MDA231-BrM
001 060
009 072
022 084
039 087
044 104
052 106
056 107
059 110
113
DMSO
BKM120
"Anti-tumoral" library (hits in H2030-BrM only)
C
Relative luminescence over DMSO (%)
2D
3D
ex vivo
Compound
ID
Carfilzomib 001
Gedatolisib 006
Dovitinib 009
Panobinostat 010
GSK461364 013
CUDC-101 016
Trametinib 022
CUDC-907 030
Ixazomib 032
Mitomycin C 039
BEZ-235 041
GSK2126458 044
AT7519 052
CNIO-DUAL 056
Sorafenib 059
Geldanamycin 060
SN-38 072
Bortezomib 084
KU-57788 087
CNIO-TRIPLE 104
Crizotinib 106
CNIO-ATR 107
Pazopanib 110
Linifanib 113
D
Col.IV NeuN
DMSO
Geldanamycin
Brain slices (Wild type)
E
Ki67 BB
DMSO
Geldanamycin
Liver slices (Wild type)
F
GI50 in vitro (µM)
Geldanamycin
H2030-BrM
PC9-BrM
393N1
MDA231-BrM
ErbB2-BrM
B16/F10-BrM
G
DMSO
DEBIO-0932
Normalized photon flux (% over control)
P=2.54E-08
MDA231-BrM

Figure EV1.

Figure EV2. Inhibition of HSP90 is effective to treat established brain metastasis.

A Representative images showing HSP70 levels in brain metastases (generated by intracardiac inoculation of H2030-BrM) found at endpoint of vehicle and DEBIO-0932-treated animals. Scale bar: 75 μm.

B Quantification of HSP70 levels shown in (A) in arbitrary fluorescent units (A.F.U.). Values are shown in box-and-whisker plots where each dot is a metastatic lesion and the line in the box corresponds to the median. The boxes go from the upper to the lower quartiles, and the whiskers go from the minimum to the maximum value (n = 6–12 metastatic lesions from 3 to 6 brains per condition). P value was calculated using two-tailed t-test.

C, D *HSP90AA1* (C) and *HSPB2* (D) expression levels obtained by qRT–PCR of H2030-BrM brain metastases obtained at endpoint of vehicle and DEBIO-0932-treated animals. Values are shown in box-and-whisker plots where every dot represents a different animal and the line in the box corresponds to the median. The boxes go from the upper to the lower quartiles, and the whiskers go from the minimum to the maximum value (n = 4 mice per experimental condition). P value was calculated using two-tailed t-test.

E Representative images of $HSP90^{+}$ non-cancer cell compartments including the medial habenula and the $Iba1^{+}$ microglia/macrophages in the metastasis-associated microenvironment from vehicle and DEBIO-0932-treated brains at the endpoint of the experiment (Fig 4A). Scale bars: Medial habenula low magnification (nucleus): 50 μm; Medial habenula high magnification (cells): 12.5 μm; Metastasis: 32 μm.

F Quantification of metastatic progression as measured by *in vivo* BLI of extracranial region of animals. Values are shown as mean ± s.e.m. (n = 23 vehicle and n = 25 DEBIO-0932-treated mice, 3 independent experiments). P value was calculated using two-tailed t-test (P values: $^{**}P < 0.01$).

G Representative images of thorax from vehicle and DEBIO-0932-treated mice at the endpoint of the experiment.

H Quantification of *ex vivo* BLI of thoracic regions at the endpoint of the experiment. Values are shown in box-and-whisker plots where every dot represents a different animal and the line in the box corresponds to the median. The boxes go from the upper to the lower quartiles, and the whiskers go from the minimum to the maximum value. (n = 21 vehicle and n = 24 DEBIO-0932-treated mice, three independent experiments). P value was calculated using two-tailed t-test.

I Animal weight from vehicle and DEBIO-0932-treated mice during the treatment period. DEBIO-0932 treatment started 2 weeks (day 14) after inoculation of cancer cells and was maintained for 3 weeks, once every 24 h during the first week and once every 48 h during the two following weeks. Values are shown as mean ± s.e.m. (n = 9 vehicle and n = 10 DEBIO-0932-treated mice).

J Quantification of mean food consumption during the interval of time between 21 and 32 days in both vehicle and DEBIO-0932-treated mice. Values are shown as mean ± s.e.m. (n = 6 mice per experimental condition. Mice were divided in two individual cages per experimental condition with 3 mice each). P value was calculated using two-tailed t-test.

K Hematoxylin eosin staining of three organs from vehicle and DEBIO-0932-treated mice at experimental endpoint. (n = 3 mice per experimental condition were evaluated for each organ). Scale bar: 50 μm.

L Kaplan-Meier curve comparing overall survival of vehicle and DEBIO-0932-treated mice following the schedule depicted in Fig 4A. (n = 9 mice treated with vehicle and n = 10 mice treated with DEBIO-0932). P value was calculated using log-rank (Mantel-Cox) test. The arrow indicates when the treatment was initiated.

M Schema of experimental design. The brain metastatic melanoma cell line B16/F10-BrM was intracranially injected to generate an established tumor so the treatment could start 3 days post-injection.

N Representative image of an established tumor 3 days post-injection. The interface between the metastasis and the associated microenvironment is well-defined. Scale bar: 50 μm (low magnification); 25 μm (high magnification).

O Representative images of slices with the brain tumor at the end of the experiment. BB: Bisbenzamide. Scale bar: 1 mm.

P Quantification of the tumor area at experimental endpoint. Values are shown in box-and-whisker plots where every dot represents a different brain and the line in the box corresponds to the median. The boxes go from the upper to the lower quartiles and the whiskers go from the minimum to the maximum value (n = 6 mice per experimental condition). P value was calculated using two-tailed t-test.

Q Representative images of human brain metastases from which BrM-PDOC were generated and evaluated as responders (Fig 4H) that showed no correlation with HSP90-dependent oncogenic drivers ALK and ROS1. Scale bar: 50 μm.

R Representative images of human brain metastases from which BrM-PDOC were generated and evaluated as responders (Fig 4H) that showed positive correlation with HSP90-dependent oncogenic drivers HER2 and BRAF. Scale bar: 50 μm. Targeting sequencing of the *EGFR* locus of a lung cancer brain metastasis patient showing a deletion in exon 19 is also shown.

S Pie chart showing the distribution of the ten BrM-PDOCs with oncogenic drivers sensitive to HSP90 inhibition (Non-HSP90 client: n = 4; *EGFR* mutant lung cancer: n = 2; $HER2^{+}$ breast cancer: n = 3; *BRAF* mutant melanoma: n = 1).

© 2022 The Authors

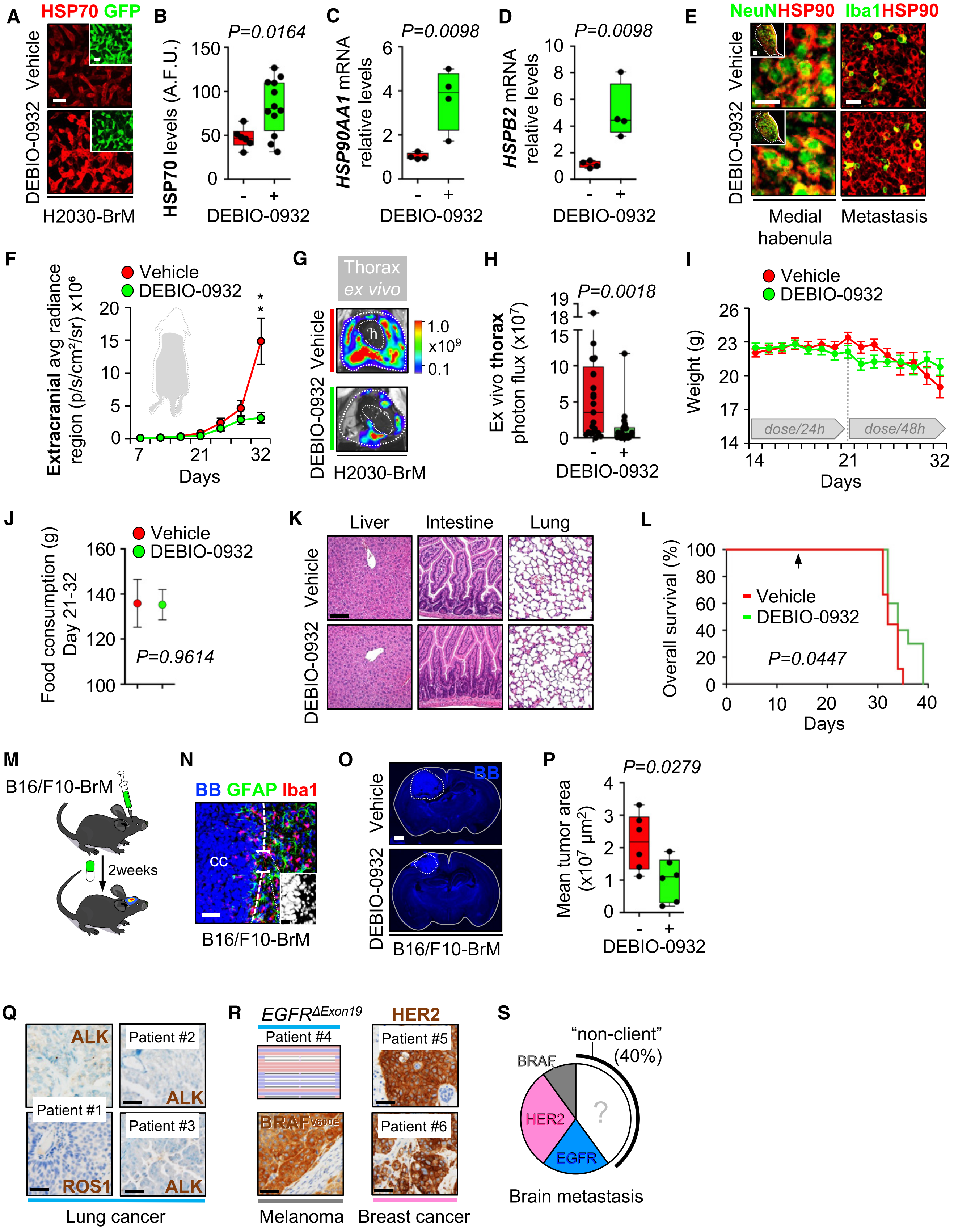
A
HSP70 GFP
Vehicle
DEBIO-0932
H2030-BrM
B
P=0.0164
HSP70 levels (A.F.U.)
DEBIO-0932
C
P=0.0098
HSP90AA1 mRNA relative levels
DEBIO-0932
D
P=0.0098
HSPB2 mRNA relative levels
DEBIO-0932
E
NeuN HSP90
Iba1 HSP90
Medial habenula
Metastasis
F
Vehicle
DEBIO-0932
Extracranial avg radiance region (p/s/cm²/sr) x10⁶
Days
G
Thorax ex vivo
h
H2030-BrM
H
P=0.0018
Ex vivo thorax photon flux (x10⁷)
I
dose/24h
dose/48h
Weight (g)
Days
J
Food consumption (g) Day 21-32
P=0.9614
K
Liver
Intestine
Lung
L
Overall survival (%)
P=0.0447
Days
M
B16/F10-BrM
2weeks
N
BB GFAP Iba1
CC
B16/F10-BrM
O
BB
B16/F10-BrM
P
P=0.0279
Mean tumor area (x10⁷ μm²)
DEBIO-0932
Q
ALK
Patient #1
Patient #2
Patient #3
ROS1
Lung cancer
R
EGFR ΔExon19
HER2
Patient #4
Patient #5
Patient #6
BRAF V600E
Melanoma
Breast cancer
S
"non-client" (40%)
BRAF
HER2
EGFR
?
Brain metastasis

Figure EV2.

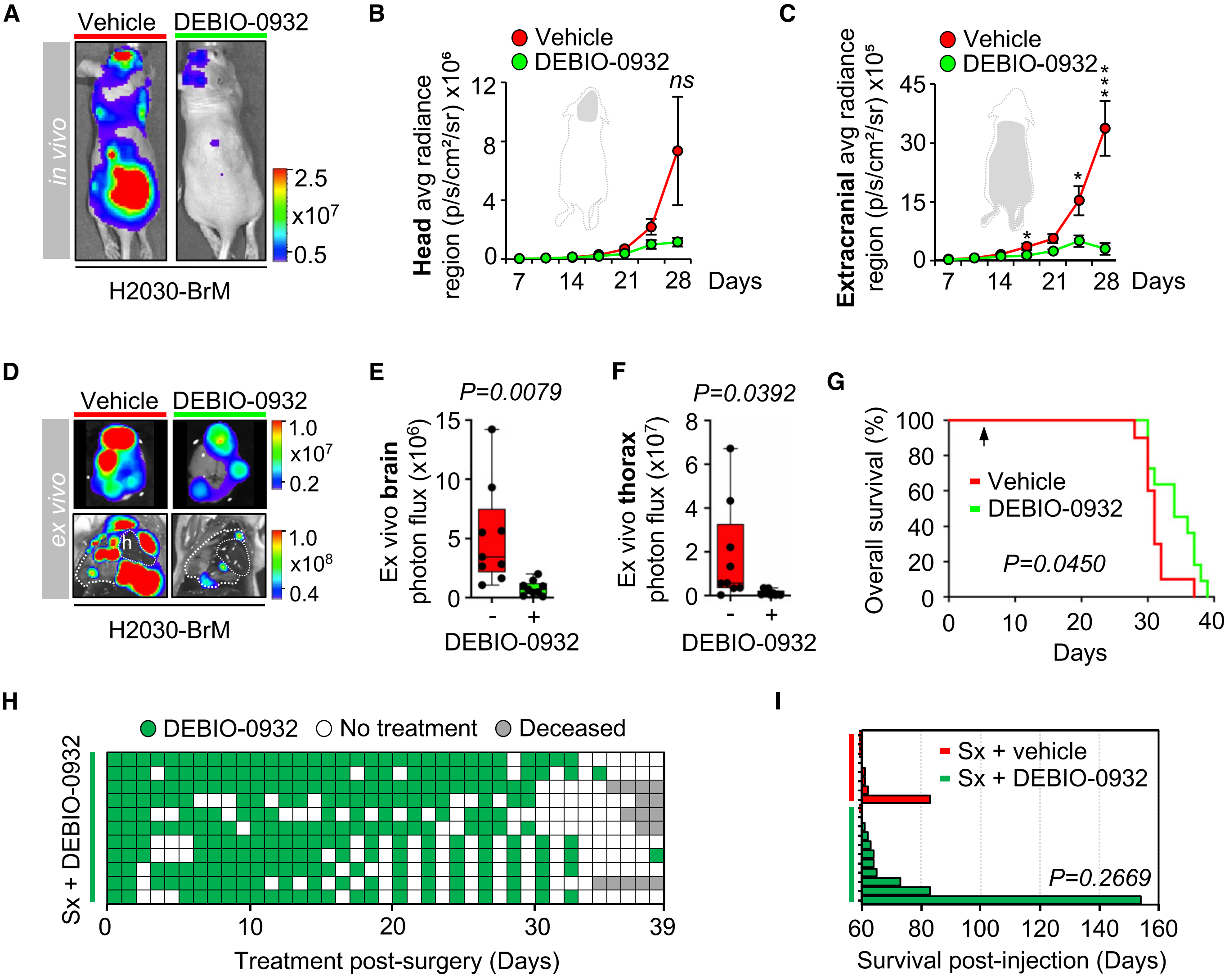

Figure EV3. Inhibition of HSP90 prevents brain metastasis initiation as well as local relapse post-surgery.

A Representative images of mice treated with DEBIO-0932 (160 mg/kg, o.g.) starting at 7 days after intracardiac inoculation of H2030-BrM cells. Treatment was given daily during the first week and every 48 h during the two following weeks.

B, C Quantification of metastatic progression as measured by *in vivo* BLI of head (B) and extracranial region (C) of animals. Values are shown as mean ± s.e.m. (n = 9 vehicle and n = 9 DEBIO-0932-treated mice, 2 independent experiments). P value was calculated using two-tailed t-test (P values: *$P < 0.05$, ***$P < 0.001$).

D Representative images of brains and thorax from vehicle and DEBIO-0932-treated mice at the endpoint of the experiment.

E, F Quantification of *ex vivo* BLI of brains (E) and thoracic regions (F) at the endpoint of the experiment. Values are shown in box-and-whisker plots where every dot represents a different animal and the line in the box corresponds to the median. The boxes go from the upper to the lower quartiles, and the whiskers go from the minimum to the maximum value (n = 9 vehicle and n = 9 DEBIO-0932-treated mice, 2 independent experiments). P value was calculated using two-tailed t-test.

G Kaplan-Meier curve comparing overall survival of vehicle and DEBIO-0932-treated mice starting 7 days post-intracardiac injection (n = 10 mice treated with vehicle and n = 11 mice treated with DEBIO-0932). P value was calculated using log-rank (Mantel-Cox) test. The arrow indicates when the treatment was initiated.

H Schema showing the individualized therapy of mice receiving surgery + DEBIO-0932 during the treatment period starting 3 days after surgery. Each row represents a mouse receiving DEBIO-0932 (green) o not (white) (n = 11 surgery + DEBIO-0932-treated mice, 2 independent experiments). Gray squares indicate decease of the corresponding animal.

I Graph showing survival of mice treated with surgery + vehicle or surgery + DEBIO-0932. The graph represents each mouse with a bar only if the survival is above the median of the group receiving surgery + vehicle (60.5 days) (n = 8 surgery + vehicle and n = 11 surgery + DEBIO-0932-treated mice, 2 independent experiments). P value was calculated using two-tailed t-test.

 © 2022 The Authors

Figure EV4. *In situ* proteomics uncovers HSP90-dependent brain metastasis mediators.

A Representative images showing RPLP1 levels in brain metastases (generated by intracardiac inoculation of H2030-BrM) found at endpoint of vehicle and DEBIO-0932-treated animals. This result was reproduced in two independent staining with different brains. Scale bars: 50 μm.

B Quantification of RPLP1 and AHR levels shown in (Figs 6E and EV4A) in arbitrary fluorescent units (A.F.U.). Values are shown in box-and-whisker plots where each dot is a metastatic lesion and the line in the box corresponds to the median. The boxes go from the upper to the lower quartiles, and the whiskers go from the minimum to the maximum value (n = 8–16 metastatic lesions from 2 to 4 brains per condition, two independent staining with different brains were performed). P value was calculated using two-tailed t-test.

C Quantification of percentage of nuclear $DDA1^+$ BB^+ cells shown in (Fig 6E). Values are shown in box-and-whisker plots where each dot is a metastatic lesion, and the line in the box corresponds to the median. The boxes go from the upper to the lower quartiles, and the whiskers go from the minimum to the maximum value (n = 16 metastatic lesions from 4 brains per condition, 2 independent staining with different brains were performed). P value was calculated using two-tailed t-test.

D Representative images showing p-ERK levels in organotypic cultures from (Fig 6A). This result was reproduced in three independent staining with organotypic cultures from different mice. Scale bar: 20 μm.

E–H Kaplan-Meier curves showing significant correlation between worse survival post-brain metastasis and high gene expression levels of *AHR* (E), *DDA1* (F), *UBE4B* (G), and *GPATCH8* (H) in a cohort of 45 breast cancer brain metastasis patients.

I, J Distribution of poor prognosis breast cancer subtypes $HER2^+$ and TNBC within the low and high gene expression level cohorts considering the signature (I) or individual genes (J).

K, L Quantification of *ex vivo* BLI of brains (K) and thoracic regions (L) of mice inoculated with H2030-BrM cells carrying shControl or sh*AHR*#2 at the endpoint of the experiment (5 weeks after injection of cancer cells). Values are shown in box-and-whisker plots where every dot represents a different animal and the line in the box corresponds to the median. The boxes go from the upper to the lower quartiles and the whiskers go from the minimum to the maximum value (n = 10 shControl mice and n = 10 sh*AHR*#2 mice). P value was calculated using two-tailed t-test.

M Representative sections of brains from shControl and sh*AHR*#1 mice 5 weeks (experimental endpoint) after intracardiac inoculation of cancer cells. The dotted lines surround the metastases (GFP^+). Scale bar: 1 mm.

N Quantification of metastases found in brains inoculated with H2030-BrM cells with sh*AHR*. Relative metastatic load was normalized to the respective control. Values are shown in dot plots where every dot represents a different brain and the dotted line corresponds to the mean ± s.e.m. (n = 8 shControl; n = 5 sh*AHR*#1; n = 3 sh*AHR*#2 mice). P value was calculated using two-tailed t-test.

O, Q H-score analysis of AHR (O) and DDA1 (R) in primary tumors with (red) or without (gray) associated relapse. Values are shown in a scattered plot where each dot is a primary tumor and the line corresponds to the median (n = 100/103 primary tumors with relapse; n = 101/103 primary tumors without relapse, respectively). P value was calculated using two-tailed t-test.

P, R Kaplan-Meier curve comparing relapse-free survival of primary tumors with high and low values of AHR (P) and DDA1 (R). P value was calculated using log-rank (Mantel-Cox) test.

© 2022 The Authors

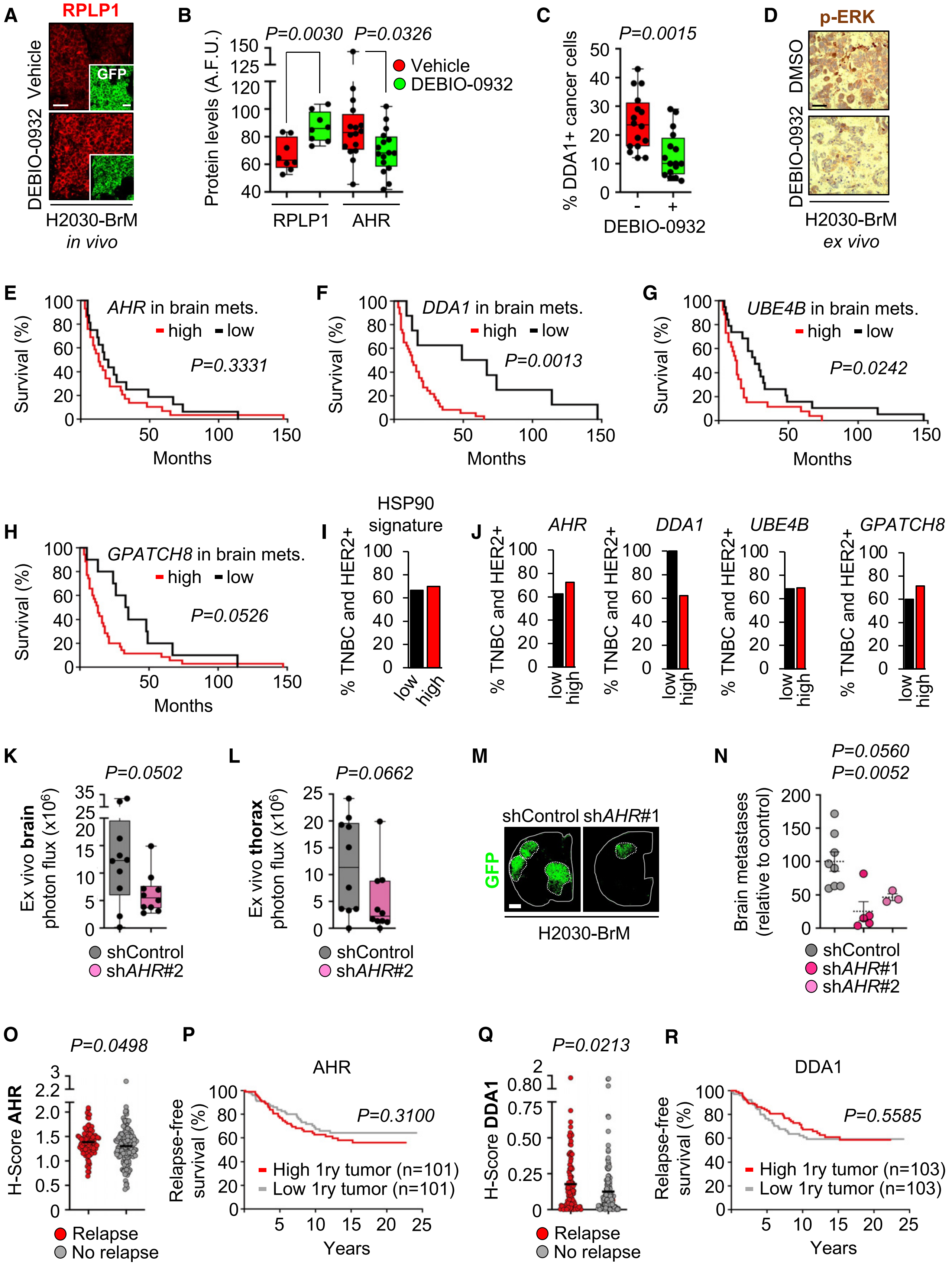
A
RPLP1
Vehicle
DEBIO-0932
GFP
H2030-BrM
in vivo
B
P=0.0030 P=0.0326
Protein levels (A.F.U.)
Vehicle
DEBIO-0932
RPLP1 AHR
C
P=0.0015
% DDA1+ cancer cells
DEBIO-0932
D
p-ERK
DMSO
DEBIO-0932
H2030-BrM
ex vivo
E
AHR in brain mets.
high low
P=0.3331
Survival (%)
Months
F
DDA1 in brain mets.
P=0.0013
G
UBE4B in brain mets.
P=0.0242
H
GPATCH8 in brain mets.
P=0.0526
I
HSP90 signature
% TNBC and HER2+
low high
J
AHR
DDA1
UBE4B
GPATCH8
K
P=0.0502
Ex vivo brain photon flux (x10⁶)
shControl
shAHR#2
L
P=0.0662
Ex vivo thorax photon flux (x10⁶)
M
GFP
shControl shAHR#1
H2030-BrM
N
P=0.0560
P=0.0052
Brain metastases (relative to control)
shControl
shAHR#1
shAHR#2
O
P=0.0498
H-Score AHR
Relapse
No relapse
P
AHR
P=0.3100
Relapse-free survival (%)
High 1ry tumor (n=101)
Low 1ry tumor (n=101)
Years
Q
P=0.0213
H-Score DDA1
R
DDA1
P=0.5585
High 1ry tumor (n=103)
Low 1ry tumor (n=103)

Figure EV4.

Figure EV5. METPlatform as a clinically compatible "avatar".

A Pie chart categorizing the 17 GB-PDOC treated with radiation (Rx) and temozolomide (TMZ) into responders (R) and non-responders (NR) independently of the dose of TMZ given (gray). Red area is labeling the single PDOC with a non-homogeneous response to the two different doses of TMZ (D).

B–D Quantification of the impact of Rx + TMZ on the proliferation of cancer cells from GB-PDOC. Values are normalized to the respective control without treatment. Each dot represents an organotypic culture from each GB-PDOC. GB-PDOC was classified as responder when the mean (line in each dot cluster) is below 40%. *P* value was calculated using two-tailed *t*-test.

E Representative images of a responder GB-PDOC stained with a DNA-damage marker, γ-H2AX (red). BB: bisbenzamide. Scale bar: 25 μm.

F Quantification of the number of γ-H2AX$^+$ cancer cells in the GB-PDOC shown in (E). Each dot represents and individual organotypic culture where the mean number of cells positive for the DNA-damage marker was measured. The dotted line represents the mean ± s.e.m. (n = 3, uncultured organotypic slices from the tumor; n = 4 GB-PDOC treated with DMSO (1%); n = 5 GB-PDOC treated with Rx + TMZ (25 μM); n = 5 GB-PDOC treated with Rx + TMZ (250 μM)). *P* value was calculated using two-tailed *t*-test.

G Quantification of LDH levels in the conditioned media of organotypic slices cultured during 3 and 7 days relative to a lysate of the same GB-PDOC. Values are shown as mean + s.e.m. (n = 3 organotypic cultures per experimental condition, each graph correspond to an individual GB-PDOC). LDH levels at 7 days measure the accumulation of the enzyme in the media from day 3 on.

H *Post hoc* quantification of the ratio between the number of months with stable disease in each patient (at cut off September 2021) and the corresponding % of proliferation after GB-PDOC treatment in METPlatform. The dotted line indicates the median. Each dot corresponds to a patient. Green dots: patient with stable disease; white dots: patients with progressive disease. The red line labels those patients with a reduction above 40% in cancer cell proliferation in the corresponding GB-PDOC.

© 2022 The Authors

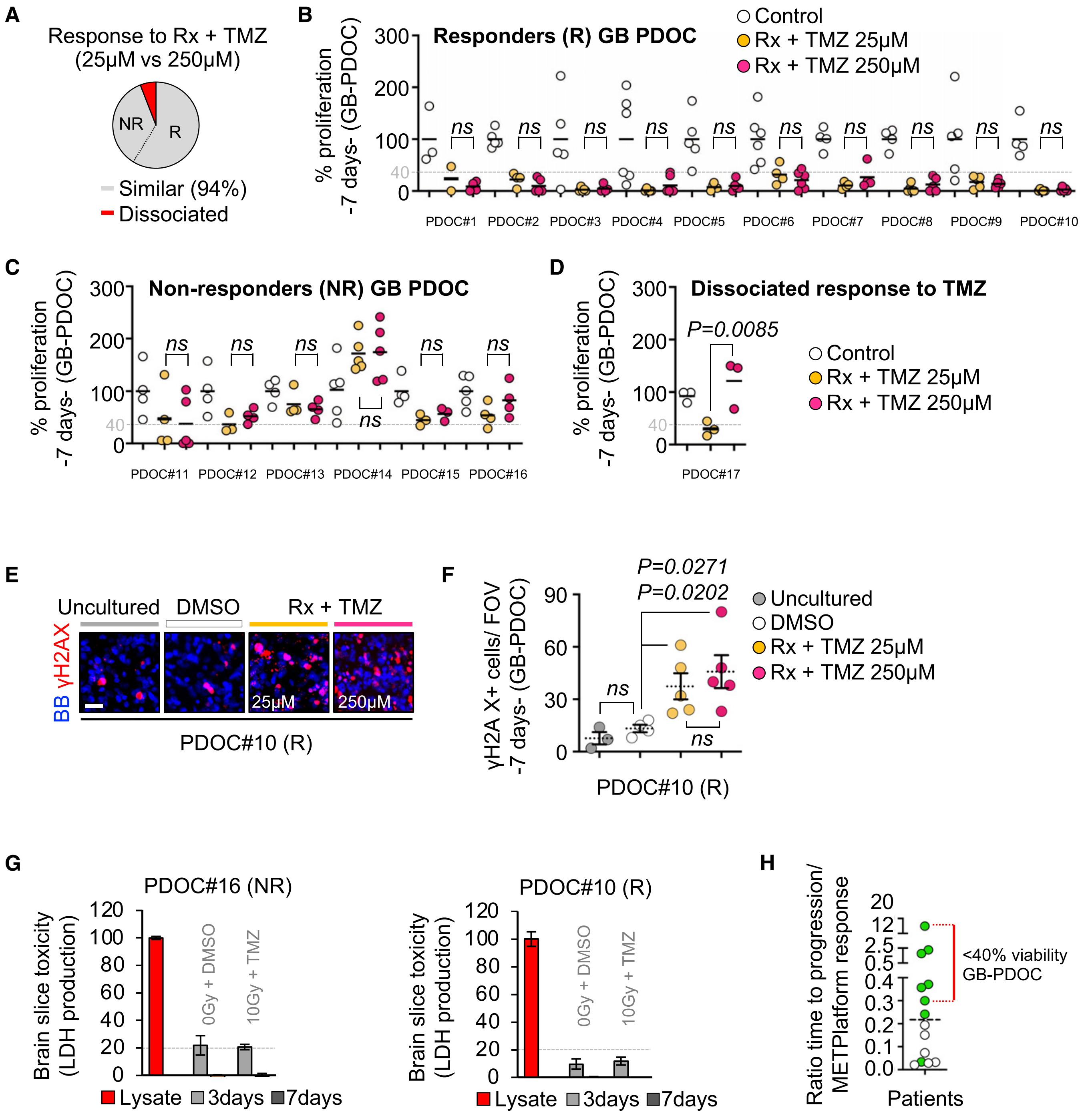

Figure EV5.

 © 2022 The Authors
