# Supplementary material for: A clinically compatible drug‐screening platform based on organotypic cultures identifies vulnerabilities to prevent and treat brain metastasis
Source: EMBO Mol Med. 2022 Feb 17;14(3):e14552. doi: 10.15252/emmm.202114552 (PMC8899920; doi:10.15252/emmm.202114552)
Supplement: Supplementary file 14 [file EMMM-14-e14552-s010.pdf]

EMBO Molecular Medicine

# A clinically-compatible drug-screening platform to prioritize anti-metastasis compounds

Lucia Zhu, Diana Retana, Pedro García-Gómez, Laura Álvaro-Espinosa, Neibla Priego, Mariam Masmudi-Martín, Natalia Yebra, Lauritz Miarka, Elena Hernández-Encinas, Carmen Blanco-Aparicio, Sonia Martinez, Cecilia Sobrino, Nuria Ajenjo, Maria-Jesus Artiga, Eva Ortega-Paino, Raul Torres, Sandra Rodríguez-Perales, Adolfo de la Lama-Zaragoza, Lourdes Calero-Felix, Concepcion Fiaño-Valverde, Pedro Delgado-López, Antonio Montalvo-Afonso, Mar Pascual-Llorente, Ángela Díaz-Piqueras, SH Nam-Cha, Cristina Barrena López, Gerard Plans Ahicart, Elena Martinez-Saez, Santiago Ramón y Cajal, Pilar Nicolás, Riccardo Soffietti, Luca Bertero, Paola Cassoni, Tobias Weiss, Javier Munoz, Juan Manuel Sepúlveda, Pedro González-León, Luis Jiménez-Roldán, Luis Miguel Moreno, Olga Esteban, Ángel Pérez-Núñez, Aurelio Hernández-Laín, Oscar Toldos, Yolanda Ruano, Lucía Alcázar, Guillermo Blasco, José Fernández-Alén, Eduardo Caleiras, Miguel Lafarga, Diego Megías, Osvaldo Graña Castro, Carolina Nör, Michael Taylor, Leonie Young, Damir Vareslija, Nicola Cosgrove, Fergus Couch, Lorena Cusso, Manuel Desco, Michael Weller, Joaquín Pastor, Manuel Valiente, Silvana Mouron, and Miguel Quintela-Fandino
**DOI: 10.15252/emmm.202114552**

*Corresponding author: Manuel Valiente (mvaliente@cnio.es)*

## Review Timeline:


| | |
|---|---|
| Submission Date: | 18th May 21 |
| Editorial Decision: | 3rd Jun 21 |
| Revision Received: | 23rd Oct 21 |
| Editorial Decision: | 1st Dec 21 |
| Author Correspondence: | 2nd Dec 21 |
| Editor Correspondence: | 10th Dec 21 |
| Revision Received: | 22nd Dec 21 |
| Accepted: | 7th Jan 22 |


*Editor: Lise Roth*

## Transaction Report:

(Note: With the exception of the correction of typographical or spelling errors that could be a source of ambiguity, letters and reports are not edited. Depending on transfer agreements, referee reports obtained elsewhere may or may not be included in this compilation. Referee reports are anonymous unless the Referee chooses to sign their reports.)

3rd Jun 2021

Dear Dr. Valiente,

Thank you for the submission of your manuscript to EMBO Molecular Medicine. We have now received feedback from the three reviewers who agreed to evaluate your manuscript. As you will see below, the reviewers raise substantial concerns on your work, which unfortunately preclude its publication in EMBO Molecular Medicine in its current form.

The reviewers find that the platform developed in this study is potentially innovative, however referee #2 in particular finds the value of the presented method unclear in comparison with other existing models. Moreover, the confusion between glioblastoma and brain metastasis should be addressed.

If you feel you can satisfactorily address these points and those listed by the referees, you may wish to submit a revised version of your manuscript. Please attach a covering letter giving details of the way in which you have handled each of the points raised by the referees. A revised manuscript will once again be subject to review and we cannot guarantee at this stage that the eventual outcome will be favorable.

Revised manuscripts should be submitted within three months of a request for revision; they will otherwise be treated as new submissions, except under exceptional circumstances in which a short extension is obtained from the editor.

When submitting your revised manuscript, please carefully review the instructions that follow below. We perform an initial quality control of all revised manuscripts before re-review; failure to include requested items will delay the evaluation of your revision.

We require:

1) A .docx formatted version of the manuscript text (including legends for main figures, EV figures and tables). Please make sure that the changes are highlighted to be clearly visible.

2) Individual production quality figure files as .eps, .tif, .jpg (one file per figure). For guidance, download the 'Figure Guide PDF' (https://www.embopress.org/page/journal/17574684/authorguide#figureformat).

3) A .docx formatted letter INCLUDING the reviewers' reports and your detailed point-by-point responses to their comments. As part of the EMBO Press transparent editorial process, the point-by-point response is part of the Review Process File (RPF), which will be published alongside your paper.

4) A complete author checklist, which you can download from our author guidelines (https://www.embopress.org/page/journal/17574684/authorguide#submissionofrevisions). Please insert information in the checklist that is also reflected in the manuscript. The completed author checklist will also be part of the RPF.

5) It is mandatory to include a 'Data Availability' section after the Materials and Methods. Before submitting your revision, primary datasets produced in this study need to be deposited in an appropriate public database, and the accession numbers and database listed under 'Data Availability'. Please remember to provide a reviewer password if the datasets are not yet public (see https://www.embopress.org/page/journal/17574684/authorguide#dataavailability).

In case you have no data that requires deposition in a public database, please state so in this section. Note that the Data Availability Section is restricted to new primary data that are part of this study.

6) For data quantification: please specify the name of the statistical test used to generate error bars and P values, the number (n) of independent experiments (specify technical or biological replicates) underlying each data point and the test used to calculate p-values in each figure legend. The figure legends should contain a basic description of n, P and the test applied. Graphs must include a description of the bars and the error bars (s.d., s.e.m.).

7) We would also encourage you to include the source data for figure panels that show essential data. Numerical data should be provided as individual .xls or .csv files (including a tab describing the data). For blots or microscopy, uncropped images should be submitted (using a zip archive if multiple images need to be supplied for one panel). Additional information on source data and instruction on how to label the files are available at
.

8) Our journal encourages inclusion of *data citations in the reference list* to directly cite datasets that were re-used and obtained from public databases. Data citations in the article text are distinct from normal bibliographical citations and should directly link to the database records from which the data can be accessed. In the main text, data citations are formatted as follows: "Data ref: Smith et al, 2001" or "Data ref: NCBI Sequence Read Archive PRJNA342805, 2017". In the Reference list,

data citations must be labeled with "[DATASET]". A data reference must provide the database name, accession number/identifiers and a resolvable link to the landing page from which the data can be accessed at the end of the reference. Further instructions are available at .

9) We replaced Supplementary Information with Expanded View (EV) Figures and Tables that are collapsible/expandable online. A maximum of 5 EV Figures can be typeset. EV Figures should be cited as 'Figure EV1, Figure EV2" etc... in the text and their respective legends should be included in the main text after the legends of regular figures.
- For the figures that you do NOT wish to display as Expanded View figures, they should be bundled together with their legends in a single PDF file called *Appendix*, which should start with a short Table of Content. Appendix figures should be referred to in the main text as: "Appendix Figure S1, Appendix Figure S2" etc.
- Additional Tables/Datasets should be labeled and referred to as Table EV1, Dataset EV1, etc. Legends have to be provided in a separate tab in case of .xls files. Alternatively, the legend can be supplied as a separate text file (README) and zipped together with the Table/Dataset file.

See detailed instructions here:
.

10) The paper explained: EMBO Molecular Medicine articles are accompanied by a summary of the articles to emphasize the major findings in the paper and their medical implications for the non-specialist reader. Please provide a draft summary of your article highlighting
- the medical issue you are addressing,
- the results obtained and
- their clinical impact.

This may be edited to ensure that readers understand the significance and context of the research. Please refer to any of our published articles for an example.

11) For more information: There is space at the end of each article to list relevant web links for further consultation by our readers. Could you identify some relevant ones and provide such information as well? Some examples are patient associations, relevant databases, OMIM/proteins/genes links, author's websites, etc...

12) Every published paper now includes a 'Synopsis' to further enhance discoverability. Synopses are displayed on the journal webpage and are freely accessible to all readers. They include a short stand first (maximum of 300 characters, including space) as well as 2-5 one-sentences bullet points that summarizes the paper. Please write the bullet points to summarize the key NEW findings. They should be designed to be complementary to the abstract - i.e. not repeat the same text. We encourage inclusion of key acronyms and quantitative information (maximum of 30 words / bullet point). Please use the passive voice. Please attach these in a separate file or send them by email, we will incorporate them accordingly.

Please also suggest a striking image or visual abstract to illustrate your article as a PNG file 550 px wide x 300-600 px high.

EMBO Molecular Medicine has a "scooping protection" policy, whereby similar findings that are published by others during review or revision are not a criterion for rejection. Should you decide to submit a revised version, I do ask that you get in touch after three months if you have not completed it, to update us on the status.

Looking forward to receiving your revised manuscript,

With kind regards,

Lise Roth

Lise Roth, PhD
Editor
EMBO Molecular Medicine

***** Reviewer's comments *****

Referee #1 (Comments on Novelty/Model System for Author):

The manuscript describes an outstanding novel platform.

Referee #1 (Remarks for Author):

This manuscript provides a new state-of-the-art model so called MET platform which is capable to measure various response

factors and to test both drug and surgical therapies. This application in personalized medicine can be realized and provide new opportunities in treatment. The manuscript is written in a clear and structured way, even if there is some confusion due to the many different methods used. It should be made clear to the reader in advance what the primary objective of the manuscript is.

Is the development of the platform in the first place (which is certainly the greatest achievement) or the development and discovery of a new agent, in which case the platform is only the means to an end. With regard to metastases research, the model already seems to be very mature, the glioma models are groundbreaking, but still require further analysis.

My comments and concerns are as follows:

The authors mention in the introduction the limitation due to the experience with in vivo mice in terms of cost. They should also consider the time factor, especially if this model is to be used for a personalized treatment approach where the patient material is tested before treatment. (extra plus for this platform)
Interspecies bias still exists due to the use of animal (murine) slices. Although availability is limited, the use of human cortex, perhaps even from the same patient, could be a considerable option in the platform for the future. In the manuscript, tissue sections were taken directly from tumors, which are relatively unstable due to the high metabolic rate. Have the authors done any experiments to monitor the "health" of the sections, such as LDH monitoring? What are the effects of the microenvironment, were glial/myeloid cells stained?
Through the platform, the ability to screen other brain cells (glia/myeloid cells) for toxicity needs to be explored more. This is because toxic effects from chemotherapy induce myeloid activation and inflammatory transformation of glial cells, leading to potential neurological impairment. The MET platform would be able to screen for this side effect as well.
See: Gibson et al., "Methotrexate Chemotherapy Induces Persistent Tri-glial Dysregulation that Underlies Chemotherapy-Related Cognitive Impairment" and "Microglia in Cancer Therapy-Related Cognitive Impairment"
The manuscript report that: "In sharp contrast, the tumor-associated microenvironment (TME) had some positive cells with lower intensity than metastases", Which cells of the microenvironment are expressing the HSP90? Even if the expression is lower, potentially a defined cell type will be targeted leading to downstream problems. See comment above.

This model is a major step into personalized medicine of primary brain tumors and an outstanding achievement. It describes an ex-vivo model to potentially predict outcome and responses within 7 days. Unfortunately, compared to metastasis models, this application is less extensively researched and lacks substantial investigation:
GBM Model: There are different limitations on the GBM model that need to be discussed:
1. Which method was used to monitor the health status of the slices, see above. GBM slices in particular form highly proliferating islands that immediately form necrotic regions in the slice culture and bias the results.
3. The removed tumor (KM-enriched regions) is usually resected, while the recurrence arises from infiltrating tumor cells. The model does not account for spatial differences between infiltrative and cellular regions of GBM, see also: Brocks at al, "The white matter is a pro-differentiative niche for glioblastoma".
5. The measure of proliferation is not an adequate readout of response to therapy.
6. 250 µmol is not an adequate concentration because only about 50 µmol TMZ can be achieved in the patient.
7. Due to the lack of follow-up data (all responders have no event), no correlation can be claimed. Even though the results are promising and the model is outstanding, the claims made from this model are over-interpreted.

Referee #2 (Comments on Novelty/Model System for Author):

There are some technical concerns. One about the in vitro comparison of H2030-BrM and MDA 231 BrM. Here they compare a 2D-system with a 3D-system (ex vivo slice). However, the current gold standard would be organoids or spheroids (3D-systems). However, the comparison is performed with a simple 2D method. Thus, the added value of the METPlatform should be confirmed in comparison with a 3D-system like spheroids or organoids. Otherwise, the statements would be an overestimation: ...Thus, METPlatform selected hits that would not have been considered as such in an in vitro approach... Altogether, our results support METPlatform as a comprehensive and more informative drug-screening platform in the context of metastasis compared to conventional cell-based assays (Fig. 1 D, Table S1).

The second and most important concern is about toxicity versus specific drug effecst. The authors write in their manuscript: We extended our ex vivo drug-screen to a triple negative breast cancer brain metastasis model, MDA231-BrM (30), to identify vulnerabilities regardless the primary tumor origin.
However, the vulnerabilities of the brain parenchyma has to be elucidated in more detail, too. Recently, Blazquez et al. (Glia, 2018) investigated BKM120 in 3D-organotypic brain slice coculture model with breast cancer cells. In this publication the concentration of 10 µM of BKM120 is significant above the IC 50 of all tested parenchymal cell types, including astrocytes (IC 50 1.51 µM) and microglia (IC 50 0.49 µM). Interestingly, this paper is not discussed or even cited in the current manuscript. In the current manuscript there are no viability tests with astrocytes and microglia (MG) at all neither for BKM120 nor for the HSP inhibitors included. In contrast, stainings for blood vessels and neurons are performed in the brain slices but not for MG and astrocytes.

Moreover, there are some concerns about the controls used in this manuscript. In the methods section they described that they normalized the results to the DMSO control which was up to 1% DMSO. This is a high DMSO concentration for the brain slice coculture model. Thus, the untreated control (without DMSO) should be added at least in the experiments for BKM120 and DEBIO-0932 (without DMSO), to the DMSO control. Moreover, the IC 50 for the whole brain slice should be tested. This could be performed by simple viability tests. These experiments are important to discriminate between unspecific additional toxicity together with DMSO and specific drug effects. This is in particular important, because they use these concentrations also in the BrM-PDOC models. Interestingly all Br-PDOC models are responsive to the single agent treatment which is amazing. Thus unspecific toxicity has to be excluded.
...The final concentration of DMSO in the tissue culture media should not exceed 1%. 2 µL of the compounds were added automatically (Beckman FX 96 tip) to 200 µL media to make it up to the final concentration for each drug. Each concentration was assayed in duplicate. Cells were exposed to the compounds for 72h and then processed for CellTiter-Glo® Luminescent Cell Viability Assay (Promega) readout according to manufacturer's instructions and read on EndVision (Perkin Elmer). Proliferation rate (%) was calculated by normalizing luminescent values obtained for each compound to values obtained with DMSO (100%). ...Remarkably, DEBIO-0932 treatment, at doses compatible with levels detected in mouse brains with metastases (Fig. 1 J), blunted tumor proliferation in all BrM-PDOC independently of their primary origin (Fig. 3, K and L).

Thrid, brain metastases reveal different histological growth pattern (HGP) like liver metastases and lung metastases (see Blazquez et al, Seminars in Cancer 2020). The authors does not describe the HGP of the Xenograft in vivo model. Thus they should weaken there statement, because not every brain metastasis infiltrate angiocentric/angio-cooptive. Moreover, it is unclear why the authors used Xenograft and not syngeneic models.

Referee #2 (Remarks for Author):

The METPlatform is besides PDX models, organoids, neurospheres, tumor initiating cells a further approach to test drug interventions derived from metastatic tissues. However, one weakness of the manuscript is that this method was not compared with one of these established models. Thus, it is not clear, if for example organoids or other 3D methods would give the same results. The next issue is a concern about toxicity versus drug-specific effects. This is described in more detal in the technical part. In particular, the used BKM120 concentrations are higher than in a previous publication (Blazquez et al, Glia 2018). In the not even cited publication there the IC 50 in particular for microglia is 10 x lower than the used concentration in this manuscript. Additionally, all experiments are always tested against the DMSO control, the completely untreated controls are not demonstrated (see also the technical concern section).
Moreover, the experiments are mainly performed in Xenografts where the histological growth pattern (HGP) could differ in comparison to immune competent models.
Finally, the findings and the method was tested in glioblastoma (GBM) to predict the response and not in brain metastases tissues, why? For example, EGFR or ALK mutated patients plus TKI therapy (Alectinib or Osimertinib) would be a perfect study cohort to demonstrate the value in BM and not in GBM.
Taken together, the manuscript is a basket of brain mets and GBM, a new drug-screening platform versus treatment prediction. However, the real added value of this new method is not so clear at this stage, in particular in comparison with other existing models.

Referee #3 (Comments on Novelty/Model System for Author):

Overall the model system is quite innovative and thoroughly tested with the exceptions noted in the remarks to the authors.

Referee #3 (Remarks for Author):

In their manuscript Zhu and colleagues develop and characterize an organotypic brain metastasis culture system as a drug-screening platform (METPlatform). Based on their initial screen which identified the HSP90 inhibitor geldanamycin as a hit, they demonstrate that a blood-brain barrier permeable HSP90 inhibitor DEBIO-0932 has activity in organotypic cultures of brain metastases from mice and humans and in murine models, including a recurrence model. They go on to show that HSP90 is frequently expressed in human brain metastases, identify functionally relevant targets of HSP90 that may contribute to its clinical activity, and demonstrate synergy of autophagy inhibitors with HSP90 inhibitors in their organotypic model. Finally, they show that the response of human GBM organotypic cultures to radiation and temozolomide correlates with time-to-progression in treated patients. The authors conclude that their drug-screening platform enables identification of personalized anti-metastatic therapies.

This is an important and timely topic with broad therapeutic implications, and the authors provide strong data to support key elements of their work. However, there are a number of deficiencies that should be addressed, including

1. It is unclear if DEBIO-0932 provides a survival benefit in murine brain metastasis models. Does the drug induce apoptosis or simply have anti-proliferative effects as demonstrated? Additionally, there is little data exploring in vivo toxicity. These are key issues for clinical translation.
2. The recurrence model (Fig. 4) does not include a drug alone arm.

3. It is unclear why AHR was selected to assess functional effects of knockdown when the other 3 targets characterized were associated with survival in patients while AHR was not.
4. The potential synergy of HSP90 and autophagy inhibition in vivo is not explored so it is unclear if the model predicts response in this case.

***** Reviewer's comments *****

## REFEREE #1

Referee #1 (Comments on Novelty/Model System for Author):

The manuscript describes an outstanding novel platform.

Referee #1 (Remarks for Author):

**1. This manuscript provides a new state-of-the-art model so called MET platform which is capable to measure various response factors and to test both drug and surgical therapies. This application in personalized medicine can be realized and provide new opportunities in treatment. The manuscript is written in a clear and structured way, even if there is some confusion due to the many different methods used. It should be made clear to the reader in advance what the primary objective of the manuscript is.**

We thank the reviewer for this comment that has allowed us to explain more clearly and earlier in the manuscript our main goal, which is precisely the potential of a new drug-screening platform with potential clinical implications. See page 4, second paragraph where we indicate time as an important aspect. In addition, we have focused the conclusions from each chapter on the platform rather than on the hit used, when it was possible.

**2. Is the development of the platform in the first place (which is certainly the greatest achievement) or the development and discovery of a new agent, in which case the platform is only the means to an end. With regard to metastases research, the model already seems to be very mature, the glioma models are groundbreaking, but still require further analysis.**

We thank the reviewer for this comment. Although we are excited about the potential of the inhibitor validated (DEBIO-0932), the key aspect of the publication is the validation of a novel platform for drug discovery, which we tried to exploit in different directions. In this sense, we have worked in the glioma more extensively so the readers could appreciate the enormous possibilities of METPLatform not only for metastasis but for brain tumors in general. See page 14 and 15, for text updates. In particular, we are extremely excited that the expansion of the previous figure 7 (now Fig 8) from 8 samples to 14 samples in the reviewed version has confirmed the potential predictive value of METPlatform as a patient “avatar”. However, we are very aware that even though we have almost duplicated the number of samples, in order to claim a correlation further validation is required (see updated text in the discussion, page 17 and 18). In this sense, we are planning follow-up clinical studies in the context of our recently established National Brain Metastasis Network (RENACER).

**My comments and concerns are as follows:**

**3. The authors mention in the introduction the limitation due to the experience with in vivo mice in terms of cost. They should also consider the time factor, especially if this model is to be used for a personalized treatment approach where the patient material is tested before treatment. (extra plus for this platform)**

This is indeed a very valuable point that we have expanded to the introduction (page 4, second paragraph) (in the initial version we only mentioned it in the discussion) so we can clearly state

one of the major advantages regarding the translation of METPlatform into the clinic or at least a major advantage to consider this possibility in the future.

**4. Interspecies bias still exists due to the use of animal (murine) slices. Although availability is limited, the use of human cortex, perhaps even from the same patient, could be a considerable option in the platform for the future. In the manuscript, tissue sections were taken directly from tumors, which are relatively unstable due to the high metabolic rate.**

Others have used human brain slices in both Neuroscience and Oncology. In our reviewed version of the manuscript, we have incorporated the analysis of the proper brain surrounding the tumor both in experimental and human tissue (see previous data on Fig EV1D-E and new data on Fig 2, Fig 4I and Appendix Fig S1). In addition, the brain without tumor has been evaluated in experimental models (Appendix Fig S1). Unfortunately, we did not have the possibility to explore healthy human brains. Overall, the analysis on the non-cancer compartment or brain tissue expands the possibilities for using METPlatform (see specific text added on page 7 and 8). We hope that, although we agree on the great interest that these findings might have, the reviewer will agree that a specific follow up is required for exploring this compartment in detail, as we have discussed in page 16, at the end of the first paragraph, page 17, at the end of the first paragraph and at the end of the second paragraph.

**5. Have the authors done any experiments to monitor the "health" of the sections, such as LDH monitoring? What are the effects of the microenvironment, were glial/myeloid cells stained?**
**Through the platform, the ability to screen other brain cells (glia/myeloid cells) for toxicity needs to be explored more. This is because toxic effects from chemotherapy induce myeloid activation and inflammatory transformation of glial cells, leading to potential neurological impairment. The MET platform would be able to screen for this side effect as well.**
**See: Gibson et al., "Methotrexate Chemotherapy Induces Persistent Tri-glial Dysregulation that Underlies Chemotherapy-Related Cognitive Impairment" and "Microglia in Cancer Therapy-Related Cognitive Impairment"**

We now provide a comprehensive evaluation that goes beyond the cancer cells. By staining the main cell types of the myeloid and glial compartments of the brain we conclude that METPlatform is also a great strategy to analyze the microenvironment. In particular, our panel includes cultured and uncultured brain organotypic cultures (Appendix Fig S1). Cultured brain slices have been incubated with DMSO, as the vehicle, BKM120, as the positive control in the drug screen, methotrexate (MTX), as an inducer of inflammatory transformation of glial cells, as suggested by the reviewer, and our top hit (Fig 2). We found different responses in the microenvironment. For instance, MTX is clearly inducing a massive activation of the tumor-associated microenvironment, (Fig 2B). A similar trend is observed with BKM120, mainly at lower concentration and on astrocytes and microglia/ macrophages specifically (Fig 2B). In contrast, DEBIO-0932 does not seem to affect the tumor-associated microenvironment in comparison with its major effect on cancer cells (Fig 2B-C). Additionally, this analysis suggested by the reviewer is allowing us to confirm that 10μM is a good starting point for METPlatform drug screening strategies (taking as reference inhibitors with IC50 in the nM range, thus with good potency and specificity respect to their targets), since those drugs not scoring at this concentration could be immediately discarded as this dose seems to be close to the maximum tolerated one based on the apparent impact in the brain microenvironment of both BKM120 and DEBIO-0932 (Fig 2B, see similarities in Olig2 reduction with these two inhibitors). In this sense, it is also crucial to mention that hits selected

with METPLatform at this dose require a subsequent titration lowering their concentration to confirm that the inhibitor is still effective (as we show in Fig 1G-H, Fig 2C). Ideally, we propose that the same analysis should be expanded to patient derived organotypic cultures, where no major differences were observed between 1-10µM regarding the microenviroment (Fig 4I) while significant effects were found in the cancer cells (Fig 4I-K, note that we have expanded human samples from 10 in the previous version to 19 in the current version, Fig 4K). Finally, even though no clear effects in the microenvironment could be observed at the concentration closer to the one that was reached in the brain in vivo (see Fig 1J), this ex vivo evaluation does not preclude the need to evaluate additional toxic effects, as we added in the discussion (see page 17, end of the second paragraph). In this sense, we have expanded the toxicity evaluation to additional aspects including weight (Fig EV2I), food-consumption (Fig EV2J) and detailed multi-organ histology (Fig EV2K)
As an additional evaluation of our drug screening platform, we have monitored LDH. Our conclusion with both organotypic cultures with and without metastases is that this method, although valid for assessing the degree of viability for the whole slice during culturing (Appendix Fig S1A, Fig 2D), it is not sensitive enough to mimic the phenotypes observed with the bioluminescence of cancer cells or the immunofluorescence (compare Fig 2C with Fig 2D for DEBIO-0932 or BKM120). In spite of this consideration, LDH quantification has allowed us to discard a major impact of culturing or the inhibitors (at any concentration tested) in the viability of brain slices (Appendix Fig S1A, Fig 2D; see text in page 6 first paragraph). Indeed, thanks to the suggestion to use LDH, we now know that this experimental approach involves a consistent 20-30% decrease in organ viability associated with the initial manipulation and establishment of cultures (Appendix Fig S1A, Fig 2D), which is stable over the incubation time (Appendix Fig S1A and Fig 2D at 3 days). Interestingly, this is in good agreement with a published reference (PMID: 31249133). Given the extensive use of brain organotypic cultures by us and many others in the field of Neuroscience and Oncology and the extensive validation of our ex vivo findings in vivo, we are confident that this expected decrease in viability intrinsic to sample processing and initial culture establishment is important to be reported but has no impact on any of our conclusions.
In summary, we are really grateful for the comment raised by the reviewers since it has allowed us to improve the characterization of METPlatform and expand its potential applications.

**6. The manuscript report that: "In sharp contrast, the tumor-associated microenvironment (TME) had some positive cells with lower intensity than metastases", Which cells of the microenvironment are expressing the HSP90? Even if the expression is lower, potentially a defined cell type will be targeted leading to downstream problems. See comment above.**

We now provide a detailed characterization of the brain without tumor respect to HSP90 levels. We have found a brain nucleus that has positive neurons as confirmed by double immunofluorescence between HSP90 and the pan-neuronal marker NeuN (Fig 3A). Although we have not found any cellular changes in the medial habenular nucleus (MHb) of the epithalamus comparing vehicle and DEBIO-0932-treated mice (Fig EV2E) or observed any behavioral changes in DEBIO-0932-treated mice, which we have substantially expanded into survival experiments (Fig EV2L, Fig EV3G) and syngeneic models (Fig EV2M-P), we have added to the discussion of the reviewed manuscript that a more thorough analysis of the potential secondary effect of DEBIO-0932 regarding the presence of HSP90 positive nucleus in the brain should be taken into account (see text page 17, end of the second paragraph) given the involvement of the habenula in complex behaviours such as mood, feeling of motivation, and reward recognition as well as addiction and withdrawal behaviours. Additionally, and thanks to the comment raised by the reviewer, we have been able to find a component of the metastasis-associated microenvironment highly positive to HSP90. In contrast to the neurons that are present in the brain nucleus independently of the presence of a tumor, Iba1+ microglia/macrophages infiltrating

the tumor showed high HSP90 levels (Fig 3C), an aspect that we did not appreciate before due to the dilution of this cell type among the abundant HSP90+ cancer cells. Given that no phenotype could be detected within the Iba1+ cells (Fig EV2E) in contrast to the evident one assigned to cancer cells (Fig 4 A-G, Fig EV2F-H, Fig EV2O-P, Fig 6, Fig 7 A-I), that the amount of HSP90+ cancer cells outnumber non-cancer cells (Fig 3C), that cancer cell-derived HSP90 is the principal contribution to the levels detected in human samples (Fig 4 H-K), and that, consequently, the risk that our proteomic and subsequent clinical correlation of identified biomarkers could be assigned to the presence of HSP90+/ Iba1+ cells instead of HSP90+/ GFP+ cancer cells is very low, we conclude that our data interpretation is correct regarding a major contribution of HSP90 from the cancer cells. However, we also consider that a project specifically focused on the potential role of HSP90 in Iba1+ cells must be developed (see text in the discussion on page 17, end of the first paragraph). As an additional piece of data confirming the importance of HSP90 signalling in metastatic cells, the functional validation of an established HSP90-dependent molecule (AHR) within cancer cells (Fig 6L-O, Fig EV4N-Q) gives us full confidence on our main conclusions.

**7. This model is a major step into personalized medicine of primary brain tumors and an outstanding achievement. It describes an ex-vivo model to potentially predict outcome and responses within 7 days. Unfortunately, compared to metastasis models, this application is less extensively researched and lacks substantial investigation:**
**GBM Model: There are different limitations on the GBM model that need to be discussed:**
**1. Which method was used to monitor the health status of the slices, see above. GBM slices in particular form highly proliferating islands that immediately form necrotic regions in the slice culture and bias the results.**
**3. The removed tumor (KM-enriched regions) is usually resected, while the recurrence arises from infiltrating tumor cells. The model does not account for spatial differences between infiltrative and cellular regions of GBM, see also: Brocks at al, "The white matter is a pro-differentiative niche for glioblastoma".**
**5. The measure of proliferation is not an adequate readout of response to therapy.**
**6. 250 µmol is not an adequate concentration because only about 50 µmol TMZ can be achieved in the patient.**
**7. Due to the lack of follow-up data (all responders have no event), no correlation can be claimed. Even though the results are promising and the model is outstanding, the claims made from this model are over-interpreted.**

7.1. In the reviewed version we have performed organotypic cultures with 17 human GBM. Given that we randomize the slices after processing the sample in the vibratome, discarding evident necrotic areas, we are very confident that our results cannot be biased by a consistent selection of this variable. Having said this we provide LDH analysis of various GBM that we have obtained during the four months we had for completing the revision. As it can be seen, although different patient-derived organotypic cultures have different LDH values, there are no major differences since values indicate <20% of decreased viability (Fig EV5G).
7. 3. Although we hoped to have the possibility of testing a relapse from any of the GBM cases that we initially used, we have not been able to get access to any within the 4 months period for the revision. However, we want to mention that partial/ subtotal resections involve that parts of the tumor left behind is detected by MRI. The detection of tumor left behind with this imaging method involves a macroscopic tumor rather than infiltrating cancer cells beyond the tumor margin. Indeed, relapses in our cohort corresponded to the outgrowth of the tumor piece detected post-surgery (Table EV6). In addition, although applied to a different tumor entity, we demonstrated experimentally that infiltrating cancer cells left behind after a complete resection could be sensitive to the same drug that tested efficacious against the established brain tumor pre-surgery (Fig 4D, 4H-I).

7. 5. We have complemented the proliferation assay performed in GBM samples with the analysis of sustained DNA damage in a responder GBM (Fig EV5E-F). However, we want to emphasize that the effect of radiation and temozolomide in GBM has been validated by its effect on proliferation (see, for instance, Fig 4c from PMID: 32753598).
7. 6. We provide an extensive analysis of two different concentrations of temozolomide (25μM and 250μM) (Fig EV5A-D) concluding that 94% of the GBM human samples did not show any difference, thus confirming that our approach is valid for scoring the sensitivity of the patient-derived organotypic culture to the therapeutic combination.
7. 7. We thank very much the comment from the reviewer. We fully agree that our results are encouraging but not enough to conclude about any definitive correlation. However, our expanded effort on characterizing GBM in the reviewed version (Fig 8) constitutes a very exciting proof-of-concept demonstrating the many potential applications of METPlatform, among which we refer to the possibility to be used as patient "avatars". Following the advice of the reviewer we have adapted the tone and conclusions on this aspect avoiding the over-interpretation of our data (Page 17 and 18; we have added "potential" and the need to "further characterize" METPlatform).

## REFEREE #2

Referee #2 (Comments on Novelty/Model System for Author):

**1. There are some technical concerns. One about the in vitro comparison of H2030-BrM and MDA 231 BrM. Here they compare a 2D-system with a 3D-system (ex vivo slice). However, the current gold standard would be organoids or spheroids (3D-systems). However, the comparison is performed with a simple 2D method. Thus, the added value of the METPlatform should be confirmed in comparison with a 3D-system like spheroids or organoids. Otherwise, the statements would be an overestimation: ...Thus, METPlatform selected hits that would not have been considered as such in an in vitro approach... Altogether, our results support METPlatform as a comprehensive and more informative drug-screening platform in the context of metastasis compared to conventional cell-based assays (Fig. 1 D, Table S1).**

We are particularly excited to share with the reviewer that, thanks to his/her suggestion, we ran an additional screen (Fig 1D, Table EV1) aimed to specifically test whether the ex vivo hits would equally score in a 3D system using spheroids. The results cannot be more interesting because evident differences are present between METPlatform and this 3D system (Fig EV1). Indeed, although not entirely similar to the results obtained in 2D, the screen ran with the spheroids is more similar to regular in vitro conditions that to the organotypic cultures (Fig 1C, Table EV1, see text page 6 second paragraph). We think that with the addition of this key comparison our platform gains the credibility to represent an additional and complementary approach to those in place for drug-screening purposes.

**2. The second and most important concern is about toxicity versus specific drug effecst. The authors write in their manuscript: We extended our ex vivo drug-screen to a triple negative breast cancer brain metastasis model, MDA231-BrM (30), to identify vulnerabilities regardless the primary tumor origin.**
**However, the vulnerabilities of the brain parenchyma has to be elucidated in more detail, too. Recently, Blazquez et al. (Glia, 2018) investigated BKM120 in 3D-organotypic brain slice coculture model with breast cancer cells. In this publication the concentration of 10 µM of BKM120 is significant above the IC 50 of all tested parenchymal cell types, including astrocytes (IC 50 1.51 µM) and microglia (IC 50 0.49 µM). Interestingly, this paper is not discussed or even cited in the current manuscript. In the current manuscript there are no viability tests with astrocytes and microglia (MG) at all neither for BKM120 nor for the HSP inhibitors included. In contrast, stainings for blood vessels and neurons are performed in the brain slices but not for MG and astrocytes.**

We want to apologize to the reviewer for having missed this relevant publication for our work. Given that the results from this publication have been used to guide us in the manuscript revision, we have now included it in the text. In addition, we fully agree on the need to expand the critical aspect of toxicity by evaluating the impact that the inhibitors might also have in the non-cancer compartment besides neurons and endothelial cells. In particular we have performed a characterization including markers for astrocytes, microglia/macrophages and oligodendrocytes (Fig 2). We have included DMSO, as the vehicle, BKM120, as the positive control in the drug screen, methotrexate (MTX), as an inducer of inflammatory transformation of glial cells, as suggested by a reviewer, and our top hit (Fig 2). We find different responses in the microenvironment. For instance, MTX is clearly inducing a massive activation of the tumor-associated microenvironment (Fig 2B). A similar trend is observed with BKM120, mainly at lower concentration and on astrocytes and microglia/ macrophages specifically (Fig 2B). Given that the

BKM120 effect we observed on the microenvironment at the lowest concentration (1uM) (Fig 2B) does not mimic the one reported on primary astrocytes and microglia under in vitro conditions, unless a higher concentration is used (10uM), we interpret this discrepancy as potentially to be explained by differences in the methodologies used (i.e., in vitro versus ex vivo). Indeed, similar to Blazquez et al. (Glia 2018), we conclude that the sensitivity of the microenvironment to BKM120 in brain slices is not evident regarding their cell viability at concentrations when cancer cells are compromised, as measured by different techniques (Fig 1B-C, Fig 2B-C). However, we want to stress out that the work by Blazquez et al. (Glia 2018) is fully focused on BKM120 offering a greater detail of analysis regarding this specific inhibitor, which we have only used as an establish inhibitor of brain metastases in order to select more potent hits out of our screen.
In this sense, focused on our leading hit, DEBIO-0932, we could not clearly detect a similar impact on the tumor-associated microenvironment as the one detected on cancer cells at the lowest concentration tested (Fig 2B-C).
Additionally, the analysis suggested by the reviewer has allowed us to confirm that 10μM is a good starting point for METPlatform drug-screening strategies (taking as reference inhibitors with IC50 in the nM range, thus with good potency and specificity respect to their targets), since those drugs not scoring at this concentration could be immediately discarded as this dose seems to be close to the maximum tolerated one based on the apparent impact on the brain microenvironment of both BKM120 and DEBIO-0932 (Fig 2B, see similarities in Olig2 reduction with these two inhibitors). In this sense, it is also crucial to mention that hits selected with METPlatform at this dose require a subsequent titration lowering their concentration to confirm that the inhibitor is still effective (as we show in Fig 1G-H, Fig 2C). Ideally, we propose that the same analysis should be expanded to patient derived organotypic cultures, where no major differences were observed between 1-10μM regarding the microenvironment (Fig 4I) while we found significant effects in the cancer cells (Fig 4I-K, note that we have expanded human samples from 10 in the previous version to 19 in the current version, Fig 4K). Finally, even though no clear effects in the microenvironment could be observed at the concentration closer to the one that was reached in the brain in vivo (see Fig 1J), this ex vivo evaluation does not preclude the need to evaluate additional toxic effects, as we mentioned in the discussion (see page 17, end of the second paragraph). In this sense, we have expanded the toxicity evaluation to additional aspects including weight (Fig EV2I), food-consumption (Fig EV2J) and detailed multi-organ histology (Fig EV2J). Similarly, given the presence of HSP90+/ Iba1+ metastasis-associated microglia/ macrophages that we are reporting in the revised version (Fig 3C), thanks to the comments raised by the reviewers, we consider mandatory to develop a follow-up project addressing the potential role of HSP90 in metastasis-associated microglia/ macrophages (see text on page 17, end of the first paragraph), in spite of the lack of any phenotype on this cell type (Fig EV2E) similar to the one we report in cancer cells (Fig 4 A-G, Fig EV2F-H, Fig EV2O-P, Fig 6, Fig 7 A-I),
As an additional evaluation of our drug screening platform, we have monitored LDH. Our conclusion with both organotypic cultures with and without metastases is that this method, although valid for assessing the degree of viability for the whole slice during culturing (Appendix Fig S1A, Fig 2D), it is not sensitive enough to mimic the phenotypes observed with the bioluminescence of cancer cells or the immunofluorescence (compare Fig 2C with Fig 2D for DEBIO-0932 or BKM120). In spite of this consideration, LDH quantification has allowed us to discard a major impact of culturing or the inhibitors (at any concentration tested) in the viability of brain slices (Appendix Fig S1A, Fig 2D; see text in page 6 first paragraph). Indeed, thanks to the suggestion of using LDH, we now know that this experimental approach involves a consistent 20-30% decrease in organ viability associated with the initial manipulation and establishment of cultures (Appendix Fig S1A, Fig 2D), which is stable over the incubation time (Appendix Fig S1A and Fig 2D at 3 days). Interestingly, this is in good agreement with a published reference (PMID: 31249133). Given the extensive use of brain organotypic cultures by us and many others in the field of Neuroscience and Oncology and the extensive validation of our ex vivo findings in vivo,

we are confident that this expected decrease in viability intrinsic to sample processing and initial culture establishment is important to be reported but has no impact on any of our conclusions.
In summary, we are really grateful for the comment raised by the reviewers since it has allowed us to improve the characterization of METPlatform and expand its potential applications.

**3. Moreover, there are some concerns about the controls used in this manuscript. In the methods section they described that they normalized the results to the DMSO control which was up to 1% DMSO. This is a high DMSO concentration for the brain slice coculture model. Thus, the untreated control (without DMSO) should be added at least in the experiments for BKM120 and DEBIO-0932 (without DMSO), to the DMSO control. Moreover, the IC 50 for the whole brain slice should be tested. This could be performed by simple viability tests. These experiments are important to discriminate between unspecific additional toxicity together with DMSO and specific drug effects. This is in particular important, because they use these concentrations also in the BrM-PDOC models. Interestingly all Br-PDOC models are responsive to the single agent treatment which is amazing. Thus unspecific toxicity has to be excluded.**
**...The final concentration of DMSO in the tissue culture media should not exceed 1%. 2 Î¼L of the compounds were added automatically (Beckman FX 96 tip) to 200 Î¼L media to make it up to the final concentration for each drug. Each concentration was assayed in duplicate. Cells were exposed to the compounds for 72h and then processed for CellTiter-GloÂ® Luminescent Cell Viability Assay (Promega) readout according to manufacturer's instructions and read on EndVision (Perkin Elmer). Proliferation rate (%) was calculated by normalizing luminescent values obtained for each compound to values obtained with DMSO (100%). ...Remarkably, DEBIO-0932 treatment, at doses compatible with levels detected in mouse brains with metastases (Fig. 1 J), blunted tumor proliferation in all BrM-PDOC independently of their primary origin (Fig. 3, K and L).**

We thank the reviewer for his/her suggestion. We have now added the DMSO IC50 and included no DMSO as an additional control in our characterization related to the microenvironment as well as LDH (Appendix Fig S1). As the reviewer can appreciate, we have not observed signs of toxicity within the dose range used, suggesting that the concentration of DMSO used in our manuscript is safe.
We are extremely grateful for the comment on PDOC. Although we initially estimated that 10 PDOC were a representative number, after duplicating the human samples we have realized that the expanded cohort offers a better estimation of the expected responses among brain metastases. While the highest dose of DEBIO-0932 is still linked to a homogeneous response, reducing it correlated with the emergence of a variable drug sensitivity, which does not correlate with the source of the brain metastasis (Fig 4H-J). Indeed, the observed heterogeneity related to DEBIO-0932 treatment at lower doses fits with the possibility that there are different degrees of dependency on the target among brain metastases. Whether the heterogeneity of therapeutic response correlates with the HSP90-dependent signature reported is an open possibility that we have now included in the discussion (page 17, at the middle of the first paragraph).

**4. Thrid, brain metastases reveal different histological growth pattern (HGP) like liver metastases and lung metastases (see Blazquez et al, Seminars in Cancer 2020). The authors does not describe the HGP of the Xenograft in vivo model. Thus they should weaken there statement, because not every brain metastasis infiltrate angiocentric/angio-cooptive. Moreover, it is unclear why the authors used Xenograft and not syngeneic models.**

We now provide a reference to the established histological growth pattern of the model initially evaluated (H2030-BrM) (Valiente et al. Cell 2014; see page 11), which is angio-cooptive. In addition, we have also expanded our pharmacological in vivo approach with an additional syngeneic model we recently reported (Priego et al. Nat Med 2018). In order to recapitulate the clinically relevant interventive strategy followed with the H2030-BrM model, where the sensitivity to a drug was measured in an established tumor, and given the limited time available to treat syngeneic models in general (this model lasts 2 weeks from inoculation of cancer cells) (Priego et al. Nat Med 2018), we opted for intracranially injecting the B16/F10-BrM melanoma cell line, which we previously characterized to generate an established tumor by day 3 post-injection so we can have at least approximately 11 days to treat the mice (Priego et al. Nat Med 2018). Even under this restrictive condition, we provide clear evidence that DEBIO-0932 is also effective against established brain metastases in vivo in a syngeneic model (Fig EV2M-P). Interestingly, the evaluation of the histological growth pattern in this model reflects an alternative strategy described as displacing macro-metastasis/organ parenchyma interface (PMID: 31647982), which suggest that DEBIO-0932 is effective against brain metastasis independently of this variable.

**Referee #2 (Remarks for Author):**

**The METPlatform is besides PDX models, organoids, neurospheres, tumor initiating cells a further approach to test drug interventions derived from metastatic tissues. However, one weakness of the manuscript is that this method was not compared with one of these established models. Thus, it is not clear, if for example organoids or other 3D methods would give the same results. The next issue is a concern about toxicity versus drug-specific effects. This is described in more detal in the technical part. In particular, the used BKM120 concentrations are higher than in a previous publication (Blazquez et al, Glia 2018). In the not even cited publication there the IC 50 in particular for microglia is 10 x lower than the used concentration in this manuscript.**
**Additionally, all experiments are always tested against the DMSO control, the completely untreated controls are not demonstrated (see also the technical concern section).**
**Moreover, the experiments are mainly performed in Xenografts where the histological growth pattern (HGP) could differ in comparison to immune competent models.**
**Finally, the findings and the method was tested in glioblastoma (GBM) to predict the response and not in brain metastases tissues, why? For example, EGFR or ALK mutated patients plus TKI therapy (Alectinib or Osimertinib) would be a perfect study cohort to demonstrate the value in BM and not in GBM.**

While we have addressed all points raised by the reviewer above, I would like to focus now on the last comment. The reviewer is suggesting an experimental plan to validate the concept of avatars in brain metastases instead of glioblastoma. I want to explain the rationale behind this apparently inconsistency since we have been considering this strategy in great detail and discarded it because of logistic reasons. The standard of care for glioblastoma is homogeneous while patients undergoing surgery for brain metastasis could receive a large variety of treatment lines after local therapy, which in part is defined by the source of the primary tumor, the clinical status of the patient and the previous lines of therapy already used. Thus, it would have been impossible to obtain a homogeneous population of brain metastases to provide a proof-of-concept experiment as the one we have been able to generate with glioma patients, which we have now expanded almost duplicating the number of samples (Fig 8). However, we are very interested in applying METPlatform to patients with brain metastases, which is indeed our final goal. In order to do this, we have created the National Network of Brain Metastasis (RENACER) (*), which includes a larger number of hospitals (12), in order to obtain enough number of samples to allocate a homogenous group of patients that could be incorporated to this type of

study. This is an ongoing effort since RENACER was officially announced last January 2021 and started operations gradually during June/July 2021. Given the additional logistic efforts required to be fully active it has not been possible address the reviewer comments given the short existence of the Network.
Having said this, our collaboration with the two main hospitals that provided us with the majority of fresh brain metastases samples used in this study has been active during the revision period in addition to RENACER. Unfortunately, no patients harboring any of the two molecular alterations suggested by the reviewer (ALK translocation, EGFR mutation) had been obtained during this period of 4 months. Indeed, we have reviewed the clinical information generated during the six-year experience collaborating with one of the main hospitals in the country (Hospital 12 de Octubre, Madrid) to provide a more specific answer so the reviewer could realize on the difficulty to complete her/ his request. The frequency of ALK mutated patients is 5-7%. However, ALK evaluation is only performed in stage III/IV patients. Given the 200 lung cancer cases diagnosed in stage III/IV at Hospital 12 de Octubre per year, the total ALK mutated ones correspond to approximately 10 cases per year. Although the incidence of brain metastases among these patients is high, not all of them will be affected. If an estimation of 70% is assumed for brain metastasis at diagnosis or post-diagnosis of the primary tumor, potentially 7 ALK mutated brain metastases per year could be expected. However, the reality is that these patients usually have multiple brain metastases, which do not qualify them for neurosurgery, and, in addition, there are systemic treatments available that are effective also in the brain. Taking these last two aspects into consideration, looking at our 6-year collaboration we only could find 1 case of ALK mutated brain metastasis that received neurosurgery. Respect to EGFR the situation is very similar: 8-9% incidence among lung cancer, we can find 15 cases with brain metastases (since the incidence of brain metastasis is lower than in ALK mutant patients) but, given that the same considerations applied (multiple brain metastases and available systemic options for treatment), the reality is that we could only find two patients of EGFR positive lung cancer that underwent neurosurgery in 6 years, which have been incorporated in our study (Fig EV2R).
In addition to the fact that these numbers are incompatible with the experiment suggested by the reviewer, the treatment that these patients would receive post-surgery is not homogeneous because both naïve and, more frequently, resistant brain metastases to targeted therapies might be operated, thus, the availability of multiple additional targeted therapies will complicate, even more, the chance to mimic in the laboratory the treatment given to the patient in real life.
As it could be easily appreciated by these comments, the efforts to obtain a homogeneous population of patients with brain metastasis requires a larger effort, which is more compatible with the Network (*) we have recently created. However, we feel that in the context of the current manuscript, the proof-of-concept generated with another brain tumor, which has been improved in the revised version (Fig 8, Fig EV5, Table EV6), should be sufficient to encourage us and others to continue working towards establishing METPlatform as a potential clinically relevant drug-screening platform.
(*) RENACER:
https://www.cnio.es/en/news/cnio-news/renacer-national-brain-metastasis-network/

**Taken together, the manuscript is a basket of brain mets and GBM, a new drug-screening platform versus treatment prediction. However, the real added value of this new method is not so clear at this stage, in particular in comparison with other existing models.**

We agree that our findings are not sufficient to establish a definitive correlation with the predictive value of METPlatform in patients but just to suggest it. However, we think that its potential transformative nature for poorly treated tumors together with our vast validation in established and new models of brain metastasis and human-derived tissues for primary and secondary brain tumors is a comprehensive characterization of a novel type of drug-screening platform. In

addition, we have made a significant effort to include very relevant aspects suggested by the reviewers such as toxicity, further in vivo validation of the molecular mechanisms and potential benefits of the top hit selected using METPlatform, expand the analysis from patient-derived material, incorporate alternative 3D drug-screening platforms for comparison and the use of syngeneic models. Thus, we hope that the reviewer could now appreciate the potential of METPlatform.

## REFEREE #3

Referee #3 (Comments on Novelty/Model System for Author):

Overall the model system is quite innovative and thoroughly tested with the exceptions noted in the remarks to the authors.

Referee #3 (Remarks for Author):

In their manuscript Zhu and colleagues develop and characterize an organotypic brain metastasis culture system as a drug-screening platform (METPlatform). Based on their initial screen which identified the HSP90 inhibitor geldanamycin as a hit, they demonstrate that a blood-brain barrier permeable HSP90 inhibitor DEBIO-0932 has activity in organotypic cultures of brain metastases from mice and humans and in murine models, including a recurrence model. They go on to show that HSP90 is frequently expressed in human brain metastases, identify functionally relevant targets of HSP90 that may contribute to its clinical activity, and demonstrate synergy of autophagy inhibitors with HSP90 inhibitors in their organotypic model. Finally, they show that the response of human GBM organotypic cultures to radiation and temozolomide correlates with time-to-progression in treated patients. The authors conclude that their drug-screening platform enables identification of personalized anti-metastatic therapies.

This is an important and timely topic with broad therapeutic implications, and the authors provide strong data to support key elements of their work. However, there are a number of deficiencies that should be addressed, including

**1. It is unclear if DEBIO-0932 provides a survival benefit in murine brain metastasis models. Does the drug induce apoptosis or simply have anti-proliferative effects as demonstrated? Additionally, there is little data exploring in vivo toxicity. These are key issues for clinical translation.**

DEBIO-0932 and survival benefit: We provide the results from the survival evaluation of novel experiments performed both with the preventive (Fig EV3G) and interventive strategies (Fig EV2L). Although DEBIO-0932 consistently increased the survival of treated mice, we consider that the benefit is limited and consequently these results should rather be interpreted as the need for additional combination strategies, as we have followed up on (Fig 7, Appendix Fig S3).

DEBIO-0932 and apoptosis: Thanks to the reviewer we now know that DEBIO-0932 induces a significant increase in apoptosis in vivo (Fig 4E, 4G).

DEBIO-0932 and in vivo toxicity: Besides the characterization of DEBIO-0932 effects in the non-tumor compartment locally in the brain (Fig EV2E), we have extended the analysis to more general signs of toxicity including weight loss (Fig EV2I), food consumption (Fig EV2J) and multi-organ evaluation by an expert pathologist (blinded to the vehicle or DEBIO-0932) groups (Fig EV2K). The results are very clear suggesting no sign of toxicity. However, given our finding describing brain nuclei with neurons expressing high levels of HSP90 (Fig 4A, Fig EV2E) we cannot discard that additional evaluation of neuronal circuit function (i.e., behavioural tests) might report potential secondary effects (see text in page 17, end of the second paragraph).

**2. The recurrence model (Fig. 4) does not include a drug alone arm.**

The experiment proposed by the reviewer cannot be performed because we can only add DEBIO-0932 post-surgery since the purpose is to evaluate its efficacy on those cancer cells left behind this local therapy. We provided vast evidences that the monotherapy with DEBIO-0932 is efficacious either in early stages (Fig EV3A-G) as well as in advanced stages (Fig 4B-G), now also expanded with a limited but consistent benefit in survival (Fig EV2L, Fig EV3G).

**3. It is unclear why AHR was selected to assess functional effects of knockdown when the other 3 targets characterized were associated with survival in patients while AHR was not.**

The rationale to pick AHR was practical since this is the only candidate that could be targeted pharmacologically. Indeed, we now provide evidence that such pharmacological approach should be exploited further given its ability to mimic the in vivo loss of function approach (Fig 6O). However, thanks to the comment raised by the reviewer we have developed loss of function approaches for the rest of the candidates, with the exception of *GPATCH8* since no loss of function strategies were available. This experiment shows a trend for a reduction in brain metastasis when other genes are targeted although it is not sufficient to reach statistical significance or it is in need of finding additional shRNA (Appendix Fig S2). Thus, although the monogenic strategies targeting the members of the HSP90 signature are not conclusive besides *AHR*, we suggest in the discussion the need to exploit them further using combined genetic approaches (see text page 13, first paragraph) given their potential clinical value.

**4. The potential synergy of HSP90 and autophagy inhibition in vivo is not explored so it is unclear if the model predicts response in this case.**

We have worked on this experiment first with chlorpromazine and later with another member from the same family of inhibitors, trifluoperazine. The results with both inhibitors have been very consistent: mice treated with these inhibitors suffered from secondary effects making not possible a regime of daily treatment at the dose initially selected (Appendix Fig S3C-F). Although we managed to find a dose that allowed us to continue the treatment at suboptimal therapeutical levels, not surprisingly, we found no benefit over the monotherapy at experimental endpoint (Appendix Fig S3G). To interpret the results appropriately we used HLPC/MS to evaluate the concentration of inhibitors reaching the brain (Appendix Fig S3H-J). This analysis unequivocally showed that the combination with any of the autophagy inhibitors compromised the amount of DEBIO-0932 reached in the brain (Appendix Fig S3H). In conclusion, translating the benefit of the combination therapy between DEBIO-0932 and autophagy inhibitors validated ex vivo has not been possible due to unexpected complications in vivo, which emphasize another limitation of METPlatform and the clear need to perform in vivo PK validation from results obtained with this platform (see text in page 14, page 16, first paragraph). However, we consider that our findings to increase the therapeutic benefits of DEBIO-0932 potency by combining with inhibitors of autophagy is still a valid strategy that must be explored further through alternative inhibitors taking into account additional aspects influencing the PK of such combination. Even more important, with the suggestion of the reviewer we got to learn/ confirm the importance of using METPlatform to reduce the use of mice for initial drug-screening but the impossibility of considering it as a replacement of a complex organism to study drug efficacy.

1st Dec 2021

Dear Manuel,

Thank you for the submission of your revised manuscript to EMBO Molecular Medicine. We have now received the enclosed reports from the three referees who had originally reviewed your manuscript. As you will see, they are now fully supportive of publication, and I am therefore please to inform you that we will be able to accept your manuscript, once the following minor points will be addressed:

1/ Main manuscript text
- Please accept the changes and only keep in track changes any new modification.
- Material and methods:
o Animal studies: please indicate the gender of the mice, as well as housing and husbandry conditions.
o For PDOC and clinical samples, include a statement that informed consent was obtained from all subjects and that the experiments conformed to the principles set out in the WMA Declaration of Helsinki and the Department of Health and Human Services Belmont Report. Please update the checklist accordingly.
o Cell culture: please indicate the origin of the cells. Kindly also indicate whether the cells were authenticated (if applicable) and tested for mycoplasma contamination.
- Thank you for providing a Data Availability section with token for reviewers. Please note that only new generated datasets must be listed here. Please also note that all datasets must be publicly available before online publication.

2/ Figures:
- Please make sure that exact p values are indicated, including for ns (not significant). Some people found that to keep the figures clear, providing a supplemental table with all exact p-values was preferable. You are welcome to do this if you want to.
- Fig. EV1F contains error bars based on n=2. Please use scatter blots showing the individual datapoints (2 points representing each the mean of the technical duplicates for each experiment?) in this case. The use of statistical test needs to be justified.

3/ Thank you for providing The Paper Explained. I added minor modifications, please let me know if you agree with the following:

PROBLEM: Brain metastasis is an unmet clinical need that currently affects up to 25% of cancer patients. A major issue remains the lack of knowledge on the vulnerabilities that, if properly exploited, could generate therapeutic opportunities.

RESULT: We report a novel drug-screening platform to study vulnerabilities of metastasis during their growth in the organ being colonized. This platform, based on organotypic cultures, effectively identified compounds that were later validated in vivo, is compatible with unbiased omics approaches, and is fully applicable to human samples.

IMPACT: Our results offer a novel therapeutic strategy that could be applicable to prevent brain metastasis in a clinically-relevant context. Furthermore, we demonstrate that METPlatform should be considered as a potential approach to facilitate the management of metastatic disease in the context of personalized cancer care.

4/ Thank you for providing a nice synopsis image. Please resize it as a TIFF/JPEG/PNG file 550 px wide x 300-600 px high.

Please also provide a synopsis text: Synopses include a short stand first (maximum of 300 characters, including space) as well as 2-5 one-sentences bullet points that summarizes the paper (maximum of 30 words / bullet point). Please write the bullet points to summarize the key NEW findings. They should be designed to be complementary to the abstract - i.e. not repeat the same text. Please use the passive voice.

5/ As part of the EMBO Publications transparent editorial process initiative (see our Editorial at http://embomolmed.embopress.org/content/2/9/329), EMBO Molecular Medicine will publish online a Review Process File (RPF) to accompany accepted manuscripts.
This file will be published in conjunction with your paper and will include the anonymous referee reports, your point-by-point response and all pertinent correspondence relating to the manuscript. Let us know whether you agree with the publication of the RPF and as here.
Please note that the Authors checklist will be published at the end of the RPF.

I look forward to receiving your revised manuscript.

With kind regards,

Lise

Lise Roth, PhD
Editor
EMBO Molecular Medicine

***** Reviewer's comments *****

Referee #1 (Remarks for Author):

Thank you for the detailed elaboration of the raised concerns. I am looking forward to see the manuscript in press.

Referee #2 (Comments on Novelty/Model System for Author):

In my previous review, I raised some critical points related to technical and methodical issues of the METPlatform. These questions were all addressed in the new manuscript or answered in the point by point response.

Referee #2 (Remarks for Author):

In my previous review, I raised some critical points related to technical and methodical issues of the METPlatform. All these questions were addressed in the new manuscript or answered in the point by point response. For example new 3D spheroid data, are included in the manuscript. Moreover, my concerns about the toxicity have been addressed by testing more concentrations with 1 -10 µm. Furthermore, in vivo data of an additional syngeneic model are also newly included and the number of patients are increased. Taken together, the authors took my criticisms very seriously and included additional data and tests, which in my opinion improved and support the main message of the manuscript.

Referee #3 (Comments on Novelty/Model System for Author):

The model system is innovative and highly disease relevant. The deficiencies have been adequately addressed in the revised manuscript.

Referee #3 (Remarks for Author):

Each of this reviewer's concerns have been addressed in the revised manuscript.

Dear Lise,

I would like to discuss with you the possibility to add a relevant piece of data we have generated during the month the paper was being reviewed. The reason why I am asking this now is because it is a piece of clinical info that reinforces our findings, not changing anything. I have prepared what it would be the updated figure and have also the updated text ready to share. I would really appreciate if you could consider my request after looking at the data because it really belongs to this publication (meaning that if we do not add it here, it cannot be used for another follow up story).

Many thanks
Best
Manuel

Dear Manuel,

We have discussed this dataset within the team, and I have also received the feedback from referee #1, who stated:
"I have looked at the new revised version in detail and find it convincing and important to add this piece of data. It strengthens the statement of the results and again validates the in vitro/vivo findings. Although it is unusual for the authors to add new data at this stage, it generally improves the manuscript."

We will therefore welcome the addition of this new results in your manuscript.

With my best wishes,

Lise

The authors performed the requested editorial changes.

7th Jan 2022

Dear Manuel,

Thank you for submitting your revised files. I am pleased to inform you that your manuscript is accepted for publication and will now be sent to our publisher to be included in the next available issue of EMBO Molecular Medicine.

As discussed previously, the datasets have to be publicly available before online publication. We included a placeholder in the manuscript for the missing DOI, please let us know as soon as you receive it.

We would like to remind you that as part of the EMBO Publications transparent editorial process initiative, EMBO Molecular Medicine will publish a Review Process File online to accompany accepted manuscripts. If you do NOT want the file to be published, please immediately inform the editorial office via e-mail.

Please read below for additional IMPORTANT information regarding your article, its publication and the production process.

Congratulations on your interesting work,

With my best wishes for 2022!

Lise

Lise Roth, Ph.D
Scientific Editor
EMBO Molecular Medicine

Follow us on Twitter @EmboMolMed
Sign up for eTOCs at embopress.org/alertsfeeds

EMBO PRESS

YOU MUST COMPLETE ALL CELLS WITH A PINK BACKGROUND

PLEASE NOTE THAT THIS CHECKLIST WILL BE PUBLISHED ALONGSIDE YOUR PAPER

| |
|---|
| Corresponding Author Name: Manuel Valiente |
| Journal Submitted to: EMBO Molecular Medicine |
| Manuscript Number: EMM-2021-14552 |

## Reporting Checklist For Life Sciences Articles (Rev. June 2017)

This checklist is used to ensure good reporting standards and to improve the reproducibility of published results. These guidelines are consistent with the Principles and Guidelines for Reporting Preclinical Research issued by the NIH in 2014. Please follow the journal's authorship guidelines in preparing your manuscript.

### A- Figures
### 1. Data

**The data shown in figures should satisfy the following conditions:**

- the data were obtained and processed according to the field's best practice and are presented to reflect the results of the experiments in an accurate and unbiased manner.
- figure panels include only data points, measurements or observations that can be compared to each other in a scientifically meaningful way.
- graphs include clearly labeled error bars for independent experiments and sample sizes. Unless justified, error bars should not be shown for technical replicates.
- if n< 5, the individual data points from each experiment should be plotted and any statistical test employed should be justified
- Source Data should be included to report the data underlying graphs. Please follow the guidelines set out in the author ship guidelines on Data Presentation.

### 2. Captions

**Each figure caption should contain the following information, for each panel where they are relevant:**

- a specification of the experimental system investigated (eg cell line, species name).
- the assay(s) and method(s) used to carry out the reported observations and measurements
- an explicit mention of the biological and chemical entity(ies) that are being measured.
- an explicit mention of the biological and chemical entity(ies) that are altered/varied/perturbed in a controlled manner.

- the exact sample size (n) for each experimental group/condition, given as a number, not a range;
- a description of the sample collection allowing the reader to understand whether the samples represent technical or biological replicates (including how many animals, litters, cultures, etc.).
- a statement of how many times the experiment shown was independently replicated in the laboratory.
- definitions of statistical methods and measures:
  - common tests, such as t-test (please specify whether paired vs. unpaired), simple $\chi^2$ tests, Wilcoxon and Mann-Whitney tests, can be unambiguously identified by name only, but more complex techniques should be described in the methods section;
  - are tests one-sided or two-sided?
  - are there adjustments for multiple comparisons?
  - exact statistical test results, e.g., P values = x but not P values < x;
  - definition of 'center values' as median or average;
  - definition of error bars as s.d. or s.e.m.

Any descriptions too long for the figure legend should be included in the methods section and/or with the source data.

**In the pink boxes below, please ensure that the answers to the following questions are reported in the manuscript itself. Every question should be answered. If the question is not relevant to your research, please write NA (non applicable). We encourage you to include a specific subsection in the methods section for statistics, reagents, animal models and human subjects.**

## USEFUL LINKS FOR COMPLETING THIS FORM

| | |
|---|---|
| http://www.antibodypedia.com | Antibodypedia |
| http://1degreebio.org | 1DegreeBio |
| http://www.equator-network.org/reporting-guidelines/improving-bioscience-research-repor | ARRIVE Guidelines |
| http://grants.nih.gov/grants/olaw/olaw.htm | NIH Guidelines in animal use |
| http://www.mrc.ac.uk/Ourresearch/Ethicsresearchguidance/Useofanimals/index.htm | MRC Guidelines on animal use |
| http://ClinicalTrials.gov | Clinical Trial registration |
| http://www.consort-statement.org | CONSORT Flow Diagram |
| http://www.consort-statement.org/checklists/view/32-consort/66-title | CONSORT Check List |
| http://www.equator-network.org/reporting-guidelines/reporting-recommendations-for-tumo | REMARK Reporting Guidelines (marker prognostic studies) |
| http://datadryad.org | Dryad |
| http://figshare.com | Figshare |
| http://www.ncbi.nlm.nih.gov/gap | dbGAP |
| http://www.ebi.ac.uk/ega | EGA |
| http://biomodels.net/ | Biomodels Database |
| http://biomodels.net/miriam/ | MIRIAM Guidelines |
| http://jjj.biochem.sun.ac.za | JWS Online |
| https://osp.od.nih.gov/biosafety-biosecurity-and-emerging-biotechnology/ | Biosecurity Documents from NIH |
| http://www.selectagents.gov/ | List of Select Agents |

### B- Statistics and general methods

Please fill out these boxes (Do not worry if you cannot see all your text once you press return)

| | |
|---|---|
| 1.a. How was the sample size chosen to ensure adequate power to detect a pre-specified effect size? | Sample size was determined according to our previous experience with both brain organotypic cultures (Martini, Valiente et al. Development. 2009; Valiente et al. Journal of Neuroscience. 2011; Valiente et al. Cell. 2014; Chen et al. Nature. 2016, Priego et al. Nature Medicine 2018) and in vivo models of brain metastasis (Valiente et al. Cell. 2014; Chen et al. Nature. 2016). Organotypic culture includes, at least, 3 individual replicas measured with bioluminescence and histological validation. In vivo brain metastasis models requires, at least, 8 individual animals measured with bioluminescence ex vivo and histological validation. This sample size requirements were achieved in each individual experiment presented in the manuscript. |
| 1.b. For animal studies, include a statement about sample size estimate even if no statistical methods were used. | In vivo brain metastasis models requires, at least, 8 individual animals per experimental condition. |
| 2. Describe inclusion/exclusion criteria if samples or animals were excluded from the analysis. Were the criteria pre-established? | No data has been excluded from any of the experiments presented in the manuscript. |
| 3. Were any steps taken to minimize the effects of subjective bias when allocating animals/samples to treatment (e.g. randomization procedure)? If yes, please describe. | Brain cultures or animals receiving treatments were randomized into control or experimental arms according to:<br>-Brain organotypic cultures: Bioluminiscence imaging at day 0 allowed to generate two groups with homogeneous values so no biased in any<br>of them could interfere with the hypothesis being tested through the use of the inhibitor.<br>-When mice were treated with inhibitors, mice were randomized according to their bioluminescent signal before treatment was started, so both treatment and vehicle group had comparable tumor-size at starting point. |
| For animal studies, include a statement about randomization even if no randomization was used. | When mice were treated with inhibitors, mice were randomized according to their bioluminescent signal before treatment was started, so both treatment and vehicle group had comparable tumor-size at starting point. |
| 4.a. Were any steps taken to minimize the effects of subjective bias during group allocation or/and when assessing results (e.g. blinding of the investigator)? If yes please describe. | For evaluation of human samples stainings, pathologists were blinded to interpret the staining of these samples. Investigators were not blinded during data collection, however data collection occurs simultaneously to both control and experimental group in the case of organotypic brain cultures, given that both are located within the same plate that was imaged, so exactly the same settings were applied to both groups. Data collection was blinded in the case of in vivo brain metastasis experiments. |
| 4.b. For animal studies, include a statement about blinding even if no blinding was done | Data collection was blinded in the case of in vivo brain metastasis experiments. |
| 5. For every figure, are statistical tests justified as appropriate? | For all statistical analyses, the statistical test(s) used are present in the figure legend, table legend, main text, or Methods section. |
| Do the data meet the assumptions of the tests (e.g., normal distribution)? Describe any methods used to assess it. | Yes. We use box plots to accurately reflect the distribution of the values for each experimental condition. |
| Is there an estimate of variation within each group of data? | A full description of the statistical parameters including central tendency (e.g. means) and variation (e.g. standard deviation or standard error of the mean) are present in the figure legend, table legend, main text, or Methods section. |

| | |
|---|---|
| Is the variance similar between the groups that are being statistically compared? | Yes |

## C- Reagents

| | |
|---|---|
| 6. To show that antibodies were profiled for use in the system under study (assay and species), provide a citation, catalog number and/or clone number, supplementary information or reference to an antibody validation profile. e.g., Antibodypedia (see link list at top right), 1DegreeBio (see link list at top right). | Primary antibodies: GFP (1:1,000; GFP-1020, Aves Labs), BrdU (1:500; ab6326, Abcam), Ki67 (1:500; ab15580, Abcam), Cleaved Caspase-3 (Asp175) (1:500; #9661, Cell Signaling), HSP90α/β F-8 (1:500; sc-13119; Santa Cruz Biotechnology), HSP70/ HSC70 W27 (1:500; sc-24; Santa Cruz Biotechnology), AHR (1:300; 31.714.200, US Biological), RPLP1 (1:100; HPA003368, Sigma-Aldrich), DDA1 (1:100; 14995-1-AP; ProteinTech), NeuN (1:500; MAB377, Millipore), NeuN (1:500; ab177487, Abcam), collagen IV (1:1000; AB756P, Millipore), GFAP (1:700; ab4674, Abcam), S100β EP1576Y (1:100; ab52642, Abcam), Iba1 (1:500; 019-19741, Wako), Olig2 (1:500; AB9610, Millipore), p-Histone H2A.X (Ser139) (1:100; 05-636, Millipore). Secondary antibodies: Alexa-Fluor anti-chicken 488, anti-chicken 555, anti-rabbit 488, anti-rat 555, anti-mouse 555, anti-rabbit 555, anti-rabbit 594, anti-rabbit 633, anti-mouse 647 (dilution 1:300; Invitrogen). All antibodies used have been used based on its previous validation as provided by the vendor and/or previous publications. |
| 7. Identify the source of cell lines and report if they were recently authenticated (e.g., by STR profiling) and tested for mycoplasma contamination. | MDA231-BrM2, ErbB2-BrM2, 393N1, H2030-BrM3 and PC9-BrM3 were obtained from MSKCC (Joan Massagué lab). B16/F10-BrM3 was generated in the Valiente Lab. Cancer cell lines used have been obtained from the same batches that were previously published (Valiente et al. Cell 2014). Cell lines were validated by morphological analysis and their behavior in vivo. To discard any cell line specific mechanism we have incorporated to our study a variety of brain metastatic models. All cell lines were tested to be free from Mycoplasma contamination. |

* for all hyperlinks, please see the table at the top right of the document

## D- Animal Models

| | |
|---|---|
| 8. Report species, strain, gender, age of animals and genetic modification status where applicable. Please detail housing and husbandry conditions and the source of animals. | We have used nude animals (Hsd:Athymic Nude-Foxn1nu) bought from Envigo and C57BL/6 animals obtained from the inbred colony at CNIO (This colony was established from C57BL/6JOlaHsd from Envigo and it is renewed every two years with new animals from the original colony). Mice used in experiments have 4-10 weeks of age. Nude mice are females. C57BL/6 mice are males and females, with an equal distribution between control and experimental cohorts. Housing and husbandry conditions were according to protocols approved by the CNIO (IACUC.030-2015), Instituto de Salud Carlos III (CBA35_2015-v2) and Comunidad de Madrid Institutional Animal Care and Use Committee (PROEX250/15 and PROEX135/19). |
| 9. For experiments involving live vertebrates, include a statement of compliance with ethical regulations and identify the committee(s) approving the experiments. | All animal experiments were performed in accordance with a protocol approved by the CNIO (IACUC.030-2015), Instituto de Salud Carlos III (CBA35_2015-v2) and Comunidad de Madrid Institutional Animal Care and Use Committee (PROEX250/15 and PROEX135/19). |
| 10. We recommend consulting the ARRIVE guidelines (see link list at top right) (PLoS Biol. 8(6), e1000412, 2010) to ensure that other relevant aspects of animal studies are adequately reported. See author guidelines, under 'Reporting Guidelines'. See also: NIH (see link list at top right) and MRC (see link list at top right) recommendations. Please confirm compliance. | All animal experiments are adequately reported in compliance with the ARRIVE guidelines. |

## E- Human Subjects

| | |
|---|---|
| 11. Identify the committee(s) approving the study protocol. | All samples were in compliance with protocols approved by their respective Institutional Review Board (IRB): University of Turin 1-18-01/2016, RENACER CEI.PI.64_2016-v3, CEI PI 25_2020-v2, CEI PI 50_2021, University of Pittsburgh IRB#PRO15050502, Royal College of Surgeons in Ireland IRB#13/09/ICORG09/07 and the Mayo Clinic Cancer Center Institutional Review Board. |
| 12. Include a statement confirming that informed consent was obtained from all subjects and that the experiments conformed to the principles set out in the WMA Declaration of Helsinki and the Department of Health and Human Services Belmont Report. | All samples were obtained under informed consent of all patients participating in the respective studies. |
| 13. For publication of patient photos, include a statement confirming that consent to publish was obtained. | N/A |
| 14. Report any restrictions on the availability (and/or on the use) of human data or samples. | No restrictions apply. |
| 15. Report the clinical trial registration number (at ClinicalTrials.gov or equivalent), where applicable. | N/A |
| 16. For phase II and III randomized controlled trials, please refer to the CONSORT flow diagram (see link list at top right) and submit the CONSORT checklist (see link list at top right) with your submission. See author guidelines, under 'Reporting Guidelines'. Please confirm you have submitted this list. | N/A |
| 17. For tumor marker prognostic studies, we recommend that you follow the REMARK reporting guidelines (see link list at top right). See author guidelines, under 'Reporting Guidelines'. Please confirm you have followed these guidelines. | We have followed the REMARK reporting guidelines. |

## F- Data Accessibility

| | |
|---|---|
| 18: Provide a "Data Availability" section at the end of the Materials & Methods, listing the accession codes for data generated in this study and deposited in a public database (e.g. RNA-Seq data: Gene Expression Omnibus GSE39462, Proteomics data: PRIDE PXD000208 etc.) Please refer to our author guidelines for 'Data Deposition'.<br><br>Data deposition in a public repository is mandatory for:<br>a. Protein, DNA and RNA sequences<br>b. Macromolecular structures<br>c. Crystallographic data for small molecules<br>d. Functional genomics data<br>e. Proteomics and molecular interactions | The datasets produced in this study are available in the following databases:<br>RNAseq data: Gene Expression Omnibus GSE153173.<br>https://www.ncbi.nlm.nih.gov/geo/query/acc.cgi?acc=GSE153173.<br>RNAseq data: Gene Expression Omnibus GSE184869.<br>https://www.ncbi.nlm.nih.gov/geo/query/acc.cgi?acc=GSE184869<br>Proteomics data: ProteomeXchange PXD020092.<br>http://proteomecentral.proteomexchange.org/cgi/GetDataset?ID=PXD020092 |
| 19. Deposition is strongly recommended for any datasets that are central and integral to the study; please consider the journal's data policy. If no structured public repository exists for a given data type, we encourage the provision of datasets in the manuscript as a Supplementary Document (see author guidelines under 'Expanded View' or in unstructured repositories such as Dryad (see link list at top right) or Figshare (see link list at top right). | All sequencing data have been deposited in public repositories. |
| 20. Access to human clinical and genomic datasets should be provided with as few restrictions as possible while respecting ethical obligations to the patients and relevant medical and legal issues. If practically possible and compatible with the individual consent agreement used in the study, such data should be deposited in one of the major public access-controlled repositories such as dbGAP (see link list at top right) or EGA (see link list at top right). | Human samples sequencing data have been deposited in public repositories. |
| 21. Computational models that are central and integral to a study should be shared without restrictions and provided in a machine-readable form. The relevant accession numbers or links should be provided. When possible, standardized format (SBML, CellML) should be used instead of scripts (e.g. MATLAB). Authors are strongly encouraged to follow the MIRIAM guidelines (see link list at top right) and deposit their model in a public database such as Biomodels (see link list at top right) or JWS Online (see link list at top right). If computer source code is provided with the paper, it should be deposited in a public repository or included in supplementary information. | N/A |

## G- Dual use research of concern

| | |
|---|---|
| 22. Could your study fall under dual use research restrictions? Please check biosecurity documents (see link list at top right) and list of select agents and toxins (APHIS/CDC) (see link list at top right). According to our biosecurity guidelines, provide a statement only if it could. | N/A |
